# Hypoxia is fine-tuned by Hif-1α and regulates mesendoderm differentiation through the Wnt/β-Catenin pathway

**DOI:** 10.1186/s12915-022-01423-y

**Published:** 2022-10-05

**Authors:** Xiaopeng Shen, Meng Li, Chunguang Wang, Zhongxian Liu, Kun Wu, Ao Wang, Chao Bi, Shan Lu, Hongan Long, Guoping Zhu

**Affiliations:** 1 Anhui Provincial Key Laboratory of Molecular Enzymology and Mechanism of Major Diseases, College of Life Sciences, Anhui Normal University, Wuhu, 241000 Anhui China; 2 Anhui Provincial Key Laboratory of the Conservation and Exploitation of Biological Resources, College of Life Sciences, Anhui Normal University, Wuhu, 241000 Anhui China; 3 Key Laboratory of Biomedicine in Gene Diseases and Health of Anhui Higher Education Institutes, College of Life Sciences, Anhui Normal University, Wuhu, 241000 Anhui China; 4 Institute of Evolution and Marine Biodiversity, KLMME, Ocean University of China, Qingdao, 266003 Shandong China

**Keywords:** Hypoxia, Hif-1α, Wnt/β-Catenin, Mesendoderm differentiation, Ectoderm differentiation, Mouse embryonic stem cells, Akt/Gsk3β axis

## Abstract

**Background:**

Hypoxia naturally happens in embryogenesis and thus serves as an important environmental factor affecting embryo development. Hif-1α, an essential hypoxia response factor, was mostly considered to mediate or synergistically regulate the effect of hypoxia on stem cells. However, the function and relationship of hypoxia and Hif-1α in regulating mesendoderm differentiation remains controversial.

**Results:**

We here discovered that hypoxia dramatically suppressed the mesendoderm differentiation and promoted the ectoderm differentiation of mouse embryonic stem cells (mESCs). However, hypoxia treatment after mesendoderm was established promoted the downstream differentiation of mesendoderm-derived lineages. These effects of hypoxia were mediated by the repression of the Wnt/β-Catenin pathway and the Wnt/β-Catenin pathway was at least partially regulated by the Akt/Gsk3β axis. Blocking the Wnt/β-Catenin pathway under normoxia using IWP2 mimicked the effects of hypoxia while activating the Wnt/β-Catenin pathway with CHIR99021 fully rescued the mesendoderm differentiation suppression caused by hypoxia. Unexpectedly, Hif-1α overexpression, in contrast to hypoxia, promoted mesendoderm differentiation and suppressed ectoderm differentiation. Knockdown of Hif-1α under normoxia and hypoxia both inhibited the mesendoderm differentiation. Moreover, hypoxia even suppressed the mesendoderm differentiation of Hif-1α knockdown mESCs, further implying that the effects of hypoxia on the mesendoderm differentiation were Hif-1α independent. Consistently, the Wnt/β-Catenin pathway was enhanced by Hif-1α overexpression and inhibited by Hif-1α knockdown. As shown by RNA-seq, unlike hypoxia, the effect of Hif-1α was relatively mild and selectively regulated part of hypoxia response genes, which fine-tuned the effect of hypoxia on mESC differentiation.

**Conclusions:**

This study revealed that hypoxia is fine-tuned by Hif-1α and regulates the mesendoderm and ectoderm differentiation by manipulating the Wnt/β-Catenin pathway, which contributed to the understanding of hypoxia-mediated regulation of development.

**Supplementary Information:**

The online version contains supplementary material available at 10.1186/s12915-022-01423-y.

## Background

Embryos experience hypoxia (typically ≤2% O_2_), which was termed relative to the ambient oxygen concentration, at specific stages and regions in the uterus [1]. When embryos become large as a result of cell proliferation, the oxygen supply by passive diffusion cannot meet the consumption demand. At that time, cell-intrinsic mechanisms that facilitate adaptation to hypoxia are activated. Among these mechanisms, the hypoxia-inducible factor 1 (HIF-1) pathway is the most well-known one. The HIF-1 pathway is mainly regulated by Hif-1α, a factor that is stabilized under hypoxia [2, 3]. Previous studies mostly demonstrated a synergetic relationship between hypoxia and the HIF-1 pathway in regulating stem cells. Pluripotency and proliferation are crucial for stem cell self-renew and differentiation potentials. However, the effects of hypoxia and the HIF-1 pathway on the pluripotency and proliferation of embryonic/pluripotent stem cells (ESCs/PSCs) are highly discrepant probably due to variable cell types, culture conditions, and treatment time in different studies [4–9]. As to stem cell differentiation, the roles of hypoxia and the HIF-1 pathway are also highly lineage-specific. Hypoxia treatment promotes the mouse ESC (mESC) differentiation towards definitive endoderm [10, 11], osteoblasts [12], hepatoblasts [13], etc. Unlike these, hypoxia impedes early ectoderm differentiation but promotes late neural differentiation partially by upregulating Sox1 [14, 15]. For the mesendoderm differentiation, the effects of hypoxia are still uncertain: hypoxia was shown to repress the expression of T-brachyury (T), an essential mesendoderm marker [10], while another report revealed that hypoxia facilitated the expression of T and FLK1 when the hypoxia treatment was administrated at a relative late differentiation stage [16]. In contrast to the conflicting effects of hypoxia on mesendoderm differentiation, the HIF-1 pathway was mostly promotive and required for the mesendoderm differentiation [3, 17, 18]. Moreover, hypoxia also regulates the differentiation of mesendoderm-derived lineages. The cardiac differentiation was enhanced by short-term hypoxia through crypto-1 [19]. The effect of hypoxia on myogenesis is controversial: two studies showed that hypoxia inhibited myogenesis through Hif-1α and Bhlhe40 [20, 21], while one reported that hypoxia promoted myogenesis via the miR-26a/HDAC6 axis [22]. In summary, hypoxia and the HIF-1 pathway regulates almost all aspects of stem cells, while some are still controversial and warrants further investigations, for example the mesendoderm differentiation.

The Wnt/β-Catenin pathway is activated primarily during development and cancer. Upon Wnt ligands binding or other stimulus treatments, the pathway is activated and ultimately leads to β-Catenin stabilization and nuclear localization. Nuclear β-Catenin cooperates with other transcriptional cofactors regulating the transcription of target genes. The role of the Wnt/β-Catenin pathway in regulating embryonic stem cell pluripotency and specification has been extensively studied. For naïve ESCs, the Wnt/β-Catenin pathway promotes their pluripotency [23–25]. However, as to primed ESCs, the Wnt/β-Catenin pathway impairs their pluripotency and promotes their differentiation towards mesendoderm rather than ectoderm [26–28]. Although the Wnt/β-Catenin pathway is essential in the mesendoderm differentiation, it should be suppressed immediately after mesendoderm is formed to facilitate the generation of mesendoderm-derived lineages, such as cardiomyocytes and smooth muscle [29–31]. The correlation between hypoxia and the Wnt/β-Catenin pathway has been studied a lot and the results are conflicting. On the one hand, hypoxia was reported to inhibit the Wnt/β-Catenin pathway via the direct repression of Wnts expression [32] and β-Catenin acetylation [33], competing binding of β-Catenin by Hif-1α and TCF-4 [34], etc. On the other hand, some studies showed that hypoxia cooperates with the Wnt/β-Catenin pathway to regulate many biological processes, such as the epithelial-mesenchymal transition [35–37] and cell proliferation [38, 39].

In this study, we determined to investigate the effects and relationship of hypoxia and Hif-1α on the mesendoderm differentiation and Wnt/β-Catenin pathway in mESCs since these remained unclear and conflicted reports existed. As a result, we discovered unexpected opposite effects of hypoxia and Hif-1α on the mESC differentiation and revealed the Wnt/β-Catenin pathway as their mutual downstream reaction mechanism. The “hypoxia” used in this study referred to 2% O_2_, which was termed relative to ambient oxygen concentration (termed “normoxia” in this study) and resembled the oxygen concentration in the uterus.

## Results

### Hypoxia dramatically suppressed the mesendoderm differentiation of mESCs

**Fig. 1.**
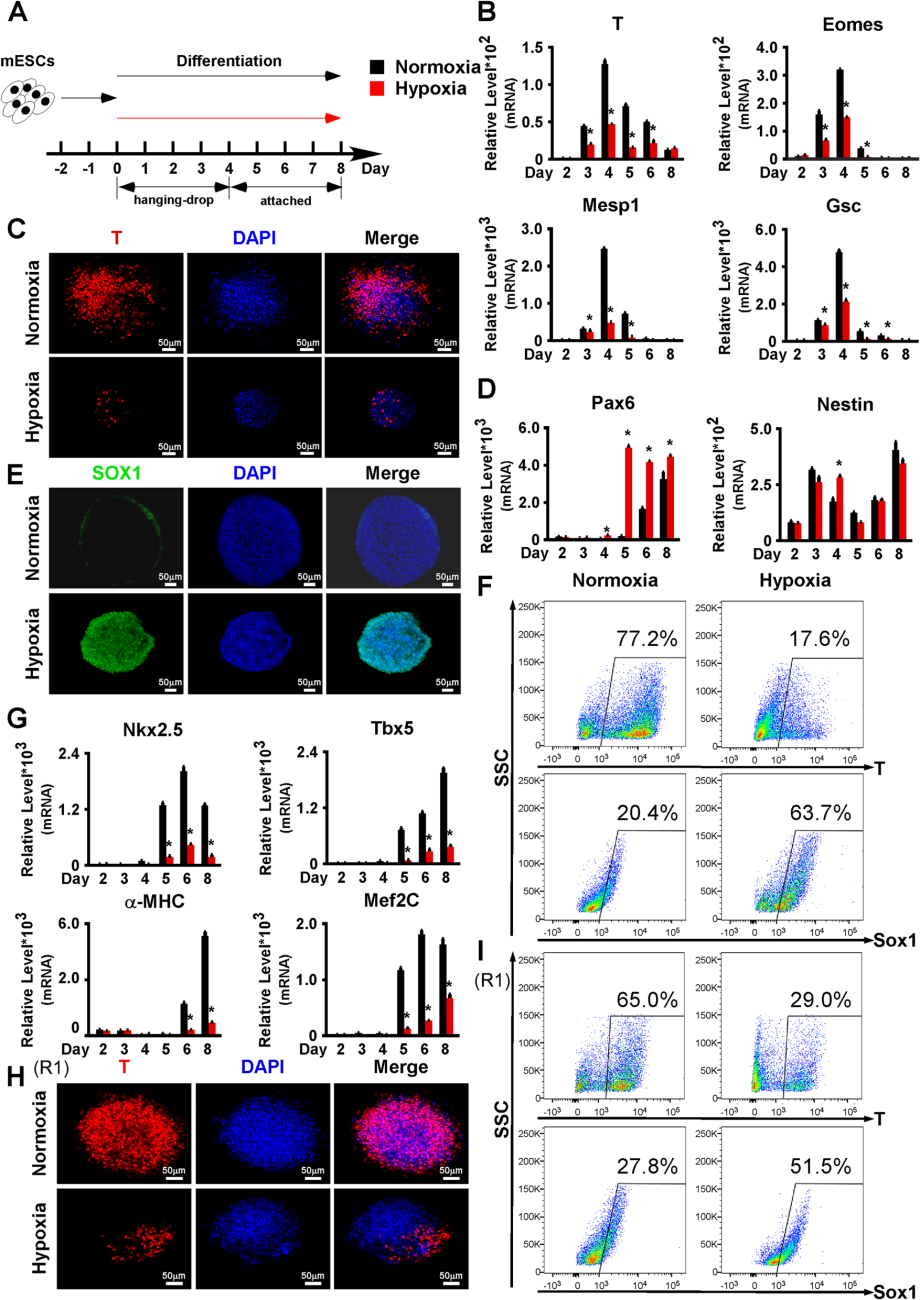
Hypoxia dramatically suppressed mesendoderm differentiation of mESCs. **A** Schematic diagram of mESC differentiation under normoxia or hypoxia. **B** Hypoxia significantly repressed the mRNA expression of mesendoderm markers (T, Eomes, Mesp1, and Gsc) in differentiating AB2.2 mESCs. **C** Hypoxia repressed T protein expression in differentiating AB2.2 mESCs. T (red) was stained on differentiation day 4. Nuclei were stained with DPAI (blue). **D** Hypoxia significantly upregulated the mRNA expression of ectoderm markers (Pax6 and Nestin) in differentiating AB2.2 mESCs. **E** Hypoxia promoted Sox1 protein expression in differentiating AB2.2 mESCs. Sox1 (green) was stained on differentiation day 4. Nuclei were stained with DPAI (blue). **F** The ratios of T+ and Sox1+ cells on differentiation day 4 ofAB2.2 mESCs were detected by flow cytometry. **G** Hypoxia significantly repressed the mRNA expression of cardiac markers (Tbx5, Mef2C, Nkx2.5, and α-MHC) in differentiating AB2.2 mESCs. **H** Hypoxia inhibited T protein expression in R1 mESCs. T (red) was stained on differentiation day 4. Nuclei were stained with DPAI (blue). **I** The ratios of T+ and Sox1+ cells on differentiation day 4 of R1 mESCs were detected by flow cytometry. *, significant (*P*<0.05)

Previous studies have revealed variable effects of hypoxia on mESCs. However, the role of hypoxia in mesendoderm differentiation continues to be debated. To further investigate the effect of hypoxia, we first performed in vitro hanging-drop differentiation on AB2.2 mESCs under normoxia and hypoxia, respectively (Fig. 1A). The results showed that the expression of mesendoderm markers (T, Eomes, Mesp1, and Gsc) was significantly inhibited by hypoxia (Fig. 1B). Consistently, T expression was negligible in the hypoxia group but robust in the normoxia group on differentiation day 4 as indicated by immunostaining (Fig. 1C). Similarly, differentiation towards the endoderm was inhibited by hypoxia, as suggested by Sox17 and Cxcr4 downregulations (Additional file 1: Fig. S1A). However, differentiation towards the ectoderm seemed to be promoted by hypoxia, which was suggested by the significant upregulation of Pax6 and Nestin (Fig. 1D). Sox1, another ectoderm marker, was also upregulated by hypoxia (Fig. 1E). Consistently, by flow cytometry on differentiation day 4, the T+ and Sox1+ cell ratios were significantly downregulated and upregulated, respectively (Fig. 1F). Particularly, the T+ cell ratio was 77.20±1.40% for the normoxia group, suggesting the differentiation protocol shown in Fig. 1A was able to efficiently trigger mesendoderm differentiation and thus will be used for most subsequent studies if not otherwise specified. Next, we tested the effects of hypoxia on mesendoderm downstream lineages, such as cardiomyocytes, endothelium, and smooth muscle. The expression of cardiac lineage markers (Tbx5, Mef2C, Nkx2-5, and α-MHC), an endothelial marker (Pecam1), and a smooth muscle marker (Acta2) was significantly decreased (Fig. 1G, S1B). To exclude the possibility that the above findings were specific to AB2.2 mESCs, we conducted the same differentiation assays on R1 mESCs as well. Similarly, the mesendoderm differentiation of R1 mESCs was significantly repressed by hypoxia as indicated by the expression of mesendoderm markers (Additional file 1: Fig. S1C, Fig. 1H), while the ectoderm differentiation was enhanced, as characterized by Pax6 and Nestin upregulations (Additional file 1: Fig. S1D). In line with AB2.2 cells, T+ and Sox1+ cell ratios were significantly downregulated and upregulated on differentiation day 4 for R1 mESCs, respectively (Fig. 1I).

Furthermore, we also validated the effects of hypoxia on mesendoderm differentiation using two different differentiation protocols. First, we performed differentiation of AB2.2 cells and supplied CHIR99021 (CHIR) on differentiation days 3–4 to boost the mesendoderm differentiation as previously reported [40, 41] (Additional file 1: Fig. S1E). Likewise, the expression of mesendoderm markers (T, Eomes, Mesp1, and Gsc) was significantly repressed by hypoxia (Additional file 1: Fig. S1F). With the CHIR treatment on days 3–4, the T+ cell ratio was 94.30±0.40% for normoxia but 73.50±2.60% for hypoxia on differentiation day 4. On the contrary, the Sox1+ cell ratio was only 3.83±0.62% for normoxia but 20.40±4.30% for hypoxia (Additional file 1: Fig. S1G). Apart from this, we examined the effects of hypoxia on the mesendoderm differentiation from epiblast like cells (EpiLCs). We induced EpiLCs from AB2.2 mESCs as reported previously [42, 43] and then triggered the mesendoderm differentiation of these cells for 24 h under normoxia and hypoxia, respectively [44]. As a result, the mesendoderm markers (T, Eomes, Mesp1, and Gsc) were significantly repressed by hypoxia (Additional file 1: Fig. S1H). Flow cytometry results also showed a lower T+ cell ratio under hypoxia, suggesting a suppressive effect of hypoxia on the mesendoderm differentiation following epiblast formation. Intriguingly, the Sox1+ cell ratio was little affected, indicating that hypoxia regulating ectoderm formation might be earlier than the epiblast (Additional file 1: Fig. S1I). Since the above studies all used mesendoderm favorable differentiation conditions, it is possible that the differentiation outcomes, especially the ectoderm markers, were biased. To exclude such possibilities, we performed a hanging-drop differentiation using a high-serum differentiation medium, which was not lineage-prone. By RT-qPCR, we found the effects of hypoxia on both the mesendoderm and ectoderm differentiations were similar to the above-described differentiation assays (Additional file 2: Fig. S2A-B).

### Hypoxia regulated the mesendoderm differentiation likely through inhibiting the Wnt/β-Catenin pathway

**Fig. 2.**
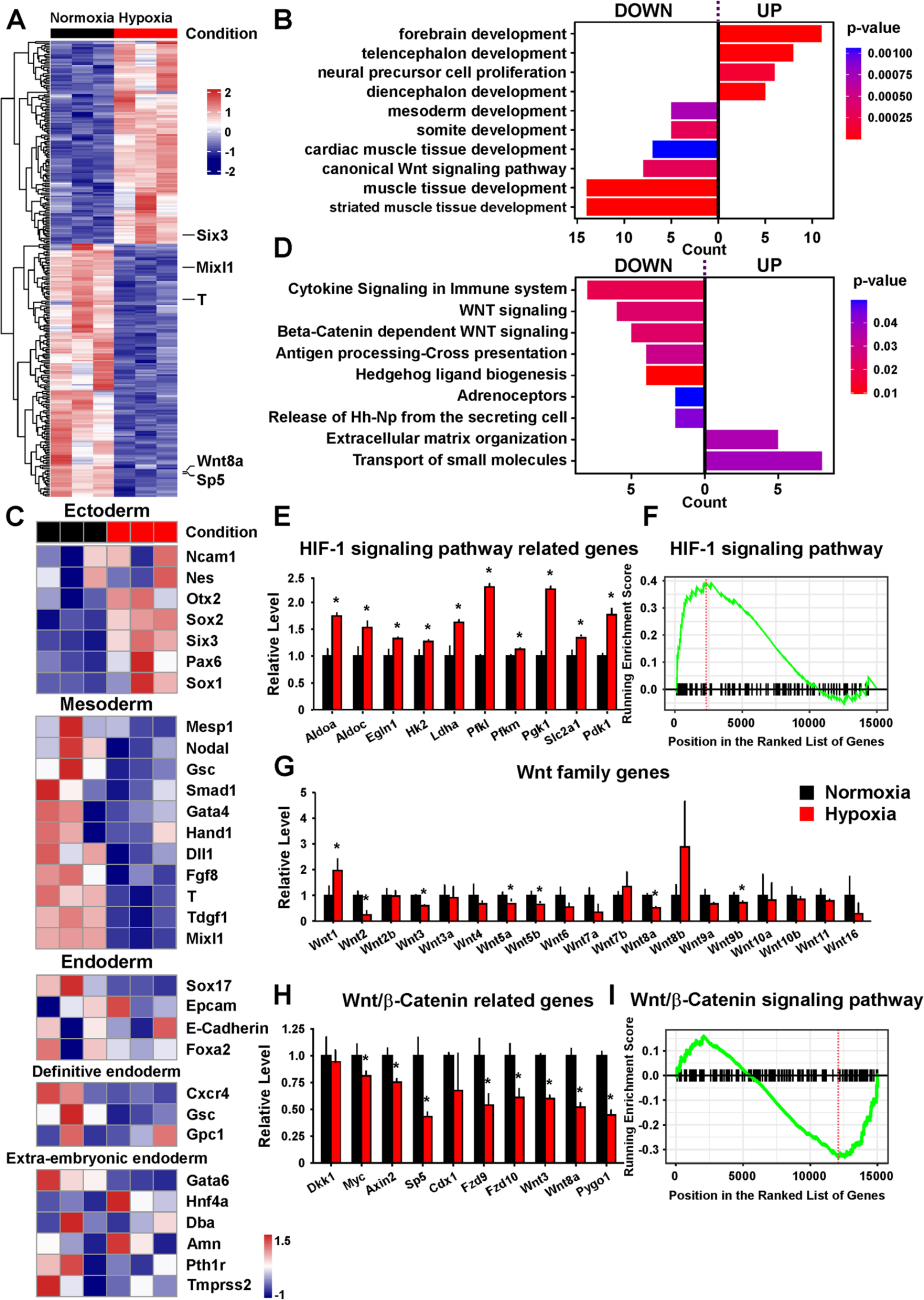
RNA-seq suggested that hypoxia regulated mESC differentiation through the Wnt/β-Catenin pathway. **A** The relative levels of the top 50 differentially expressed genes (DEGs) in AB2.2 mESCs on differentiation day 4 under hypoxia versus normoxia. **B** The GO terms of the DEGs under hypoxia versus normoxia. **C** The relative levels of the marker genes of three germ layers in AB2.2 mESCs on differentiation day 4 under hypoxia versus normoxia. **D** The pathway analysis of the DEGs under hypoxia versus normoxia. **E** The expression of genes related to the HIF-1 signaling pathway in AB2.2 mESCs on differentiation day 4 under hypoxia versus normoxia. **F** GSEA showed that HIF-1 signaling pathway activation was enhanced by hypoxia. **G** The expression of Wnt family genes in AB2.2 mESCs on differentiation day 4 under hypoxia versus normoxia. **H** The expression of Wnt/β-Catenin pathway-related genes in AB2.2 mESCs on differentiation day 4 under hypoxia versus normoxia. **I** GSEA showed that the Wnt/β-Catenin pathway was repressed by hypoxia. *, significant (*P*<0.05)

To determine how hypoxia regulated the mesendoderm differentiation, we first performed RNA-sequencing (RNA-seq) on the AB2.2 cells on differentiation day 4 under normoxia and hypoxia, respectively. Principal component analysis (PCA) analysis showed that the gene expression patterns under the two conditions were distinct (Additional file 3: Fig. S3A). To determine the differentially expressed genes (DEGs), |Log_2_(Fold Change)| (|LogFC|) > 1 and the adjusted *P*-value < 0.05 were used as the cutoff criteria. There were 126 upregulated and 156 downregulated genes in the hypoxia group compared to the normoxia group (Additional file 3: Fig. S3B). We plotted a heatmap for these DEGs. The mesendoderm markers T and Mixl1 were significantly downregulated, whereas Six3, an ectoderm maker, was upregulated. Moreover, Wnt8a, a Wnt/β-Catenin pathway member, and Sp5, a Wnt/β-Catenin pathway downstream target, were both significantly downregulated, suggesting that the Wnt/β-Catenin pathway might be repressed under hypoxia during mESC differentiation (Fig. 2A). Gene ontology (GO) analysis showed that the ectoderm development-related biological processes were promoted, whereas the development of mesendoderm and its derived lineage-related processes were suppressed by hypoxia (Fig. 2B). We then studied the expression of marker genes for all three germ layers. The expression of mesendoderm markers was downregulated, whereas that of most ectoderm marker genes was upregulated under hypoxia. As to endoderm, the expression of definitive endoderm (DE) and extraembryonic endoderm (XEN) -related markers was differentially regulated: the markers that were shared by DE and XEN and DE-specific were downregulated, while the XEN-specific markers were not uniformly regulated (Fig. 2C).

Subsequently, we studied the underlying regulatory mechanism of hypoxia regulating mESC differentiation. We first studied the significantly altered pathways under hypoxia (Fig. 2D). The Wnt (especially Wnt/β-Catenin) pathway and hedgehog ligand biogenesis were among the significant repressed pathways. Regarding that hedgehog ligand biogenesis was suppressed by hypoxia, which might affect hedgehog (Hh) signaling, we studied the expression changes of Hh-related genes under hypoxia and found that Shh, Dhh, and Ihh were truly downregulated, whereas their downstream factors, such as Gli1/2 and Ptch1/2, were upregulated (Additional file 3: Fig. S3C). This pattern implied that the repression of hedgehog ligand biogenesis did not eventually lead to a repression of Hh signaling. For such reasons, we hypothesized that the Wnt, especially the Wnt/β-Catenin pathway, might be responsible for the effects of hypoxia on mESC differentiation. Intriguingly, we did not identify the HIF-1 signaling in the significantly altered pathways in Fig. 2D. To investigate the change in the HIF-1 signaling, we first studied the relative levels of HIF-1 signaling-related genes and found that these genes were mostly significantly upregulated under hypoxia (Fig. 2E). A GSEA also showed that the HIF-1 signaling was promoted under hypoxia (Fig. 2F).

Next, we studied the relative levels of Wnt family members and found that these genes were mainly downregulated except for Wnt1, Wnt7b, and Wnt8b (Fig. 2G). As other studies suggested, Wnt1, Wnt7b, and Wnt8b all played important roles in the development and function of neuroectoderm and brain [45–48], which suggested that these Wnt members might partially mediate the function of hypoxia on ectoderm differentiation. There are two Wnt-involved pathways, Wnt/β-Catenin (also known as the canonical Wnt) and β-Catenin-independent Wnt (also known as the noncanonical Wnt) pathways. We found that major Wnt/β-Catenin pathway components were significantly downregulated under hypoxia, which agreed with the GSEA results (Fig. 2H,I). Regarding the β-Catenin-independent Wnt pathway, although its classical ligands, Wnt5a and Wnt5b, were significantly downregulated (Fig. 2G), its other components were not uniformly regulated (Additional file 3: Fig. S3D) and the GSEA also showed bidirectional changes (Additional file 3: Fig. S3E), indicating the β-Catenin-independent Wnt pathway might not be markedly affected by hypoxia. Moreover, to further validate the effects of hypoxia at an earlier differentiation stage, we also performed RNA-seq on the AB2.2 cells on differentiation day 2 under normoxia and hypoxia, respectively. Consistently, the expression changes of essential genes and GSEA indicated the HIF-1 signaling and Wnt/β-Catenin pathway were promoted and repressed by hypoxia, respectively (Additional file 4: Fig. S4A-D). These results showed that hypoxia suppressed the Wnt/β-Catenin pathway. Combined with the established role of the Wnt/β-Catenin pathway in development [26–28], these results implied that hypoxia might regulate mESC differentiation by inhibiting the Wnt/β-Catenin pathway.

### Hypoxia inhibited the Wnt/β-Catenin pathway by repressing Gsk3β phosphorylation

**Fig. 3.**
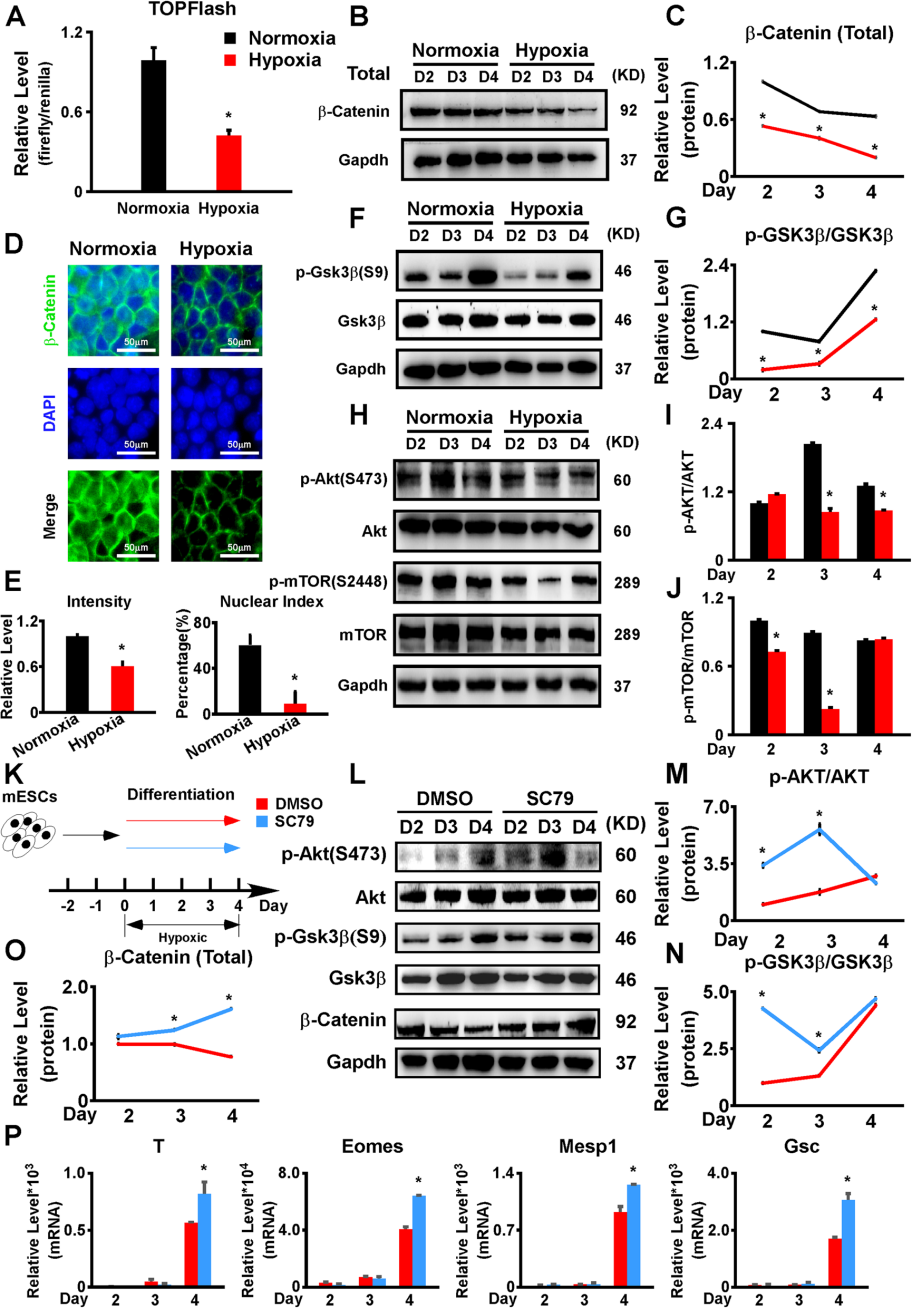
Hypoxia inhibited the Wnt/β-Catenin pathway via the Akt/Gsk3β axis. **A** TOPFlash assays showed that hypoxia remarkably repressed the Wnt/β-Catenin pathway. **B, C** β-Catenin expression was repressed by hypoxia in AB2.2 mESCs undergoing differentiation. **D** Hypoxia repressed both the expression and nuclear localization of β-Catenin in differentiating AB2.2 mESCs. β-Catenin (green) was stained on differentiation day 4. Nuclei were stained with DPAI (blue). **E** Quantification was performed on the images of **D** using the ImageJ software. The intensity was calculated as the ratio of the β-Catenin positive area versus the whole area. The nuclear index was the ratio of β-Catenin positive nuclei versus total nuclei number. **F, G** p-Gsk3β, **H–J** p-Akt, and p-mTOR expression was significantly inhibited by hypoxia. **K** Schematic diagram of AB2.2 mESC differentiation treated with or without SC79, an Akt activator, under hypoxia. **L–O** p-Akt, p-Gsk3β, and β-Catenin were significantly upregulated with SC79 under hypoxia. **P** SC79 treatment upregulated the repression of mesendoderm marker expression (T, Eomes, Mesp1, and Gsc) under hypoxia. *, significant (*P*<0.05)

To verify the RNA-seq results, we first performed RT-qPCR to evaluate the expression changes of several important Wnt/β-Catenin pathway genes. Under hypoxia, Wnt3 and Wnt8a were significantly downregulated on days 3–4 of the AB2.2 mESC differentiation (Additional file 5: Fig. S5A). Sp5 and Cdx1, the Wnt/β-Catenin pathway downstream targets, were also significantly downregulated (Additional file 5: Fig. S5B). As to R1 mESC differentiation, Wnt3, Wnt8a, Sp5, and Cdx1 were downregulated by hypoxia as well (Additional file 5: Fig. S5C-D). Moreover, we also performed a TOPFlash luciferase assay under normoxia and hypoxia, respectively. The hypoxia group showed a remarkable decrease in luciferase activity, which indicated that hypoxia inhibited the Wnt/β-Catenin pathway (Fig. 3A). Next, we investigated the expression pattern of β-Catenin during the AB2.2 mESC differentiation and found that the total β-Catenin level was downregulated under hypoxia (Fig. 3B,C). However, the expression of cytoplasmic β-Catenin was elevated for the first 3 days under hypoxia and downregulated on day 4, while nuclear β-Catenin was significantly repressed throughout the differentiation (Additional file 5: Fig. S5E-G). Immunostaining against β-Catenin was performed on differentiation day 4, showing that both the intensity and nuclear localization of β-Catenin were repressed by hypoxia (Fig. 3D,E). These results suggested that hypoxia not only inhibited the total level but also inhibited the nuclear localization of β-Catenin. As Gsk3β is an essential upstream regulator of β-Catenin in the Wnt/β-Catenin pathway, we next studied the expression and phosphorylation levels of Gsk3β. We found that the phosphorylated Gsk3β (S9) level was significantly reduced under hypoxia (Fig. 3F,G). To determine how the Gsk3β phosphorylation was affected, we detected the phosphorylated Akt (S473) (p-Akt(S473)) levels and found that the p-Akt(S473) was downregulated under hypoxia. Consistently, phosphorylated mTOR (S2448) (p-mTOR(S2448)), which was a downstream response factor of p-Akt(S473), was repressed under hypoxia (Fig. 3H–J). This was supported by GSEA which showed the PI3K/Akt signaling was repressed by hypoxia on both differentiation day 2 and day 4 (Additional file 4: Fig. S4E, Additional file 3: Fig. S3F). To further validate the role of the Akt/Gsk3β axis in regulating the Wnt/β-Catenin pathway and mesendoderm specification, we added SC79, a specific Akt activator, to the AB2.2 mESC differentiation performed under hypoxia (Fig. 3K). The phosphorylation of both Akt and Gsk3β and the total levels of β-Catenin were significantly upregulated (Fig. 3L–O). Likewise, the mesendoderm markers (T, Eomes, Mesp1, and Gsc) were significantly upregulated with the SC79 treatment (Fig. 3P). Taking together, these results suggested that hypoxia might suppress the mesendoderm differentiation by repressing the nuclear β-Catenin level through the Akt/Gsk3β axis.

### The Wnt/β-Catenin pathway mediated the hypoxia-induced repression of the mesendoderm differentiation

**Fig. 4.**
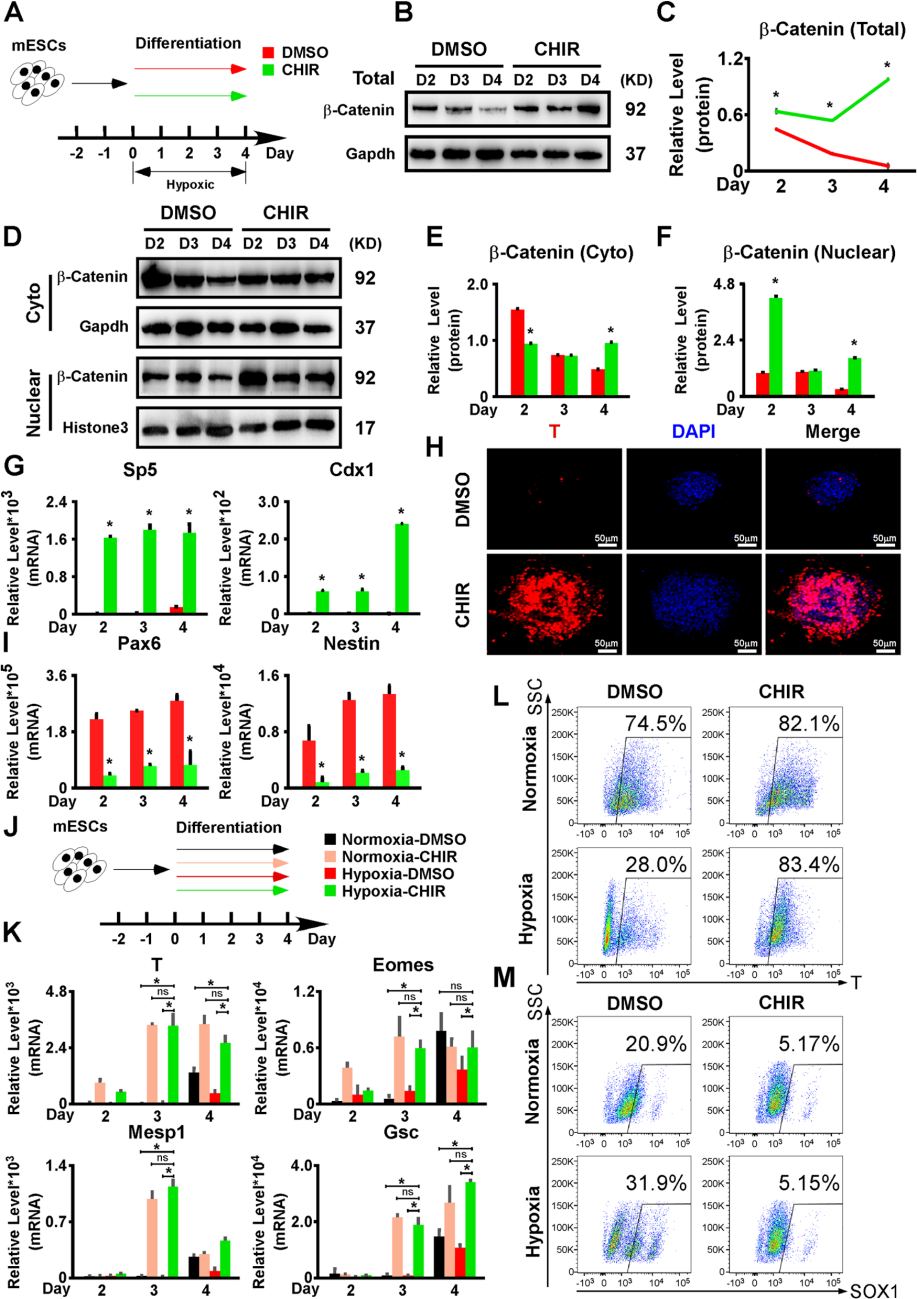
The activation of the Wnt/β-Catenin pathway successfully rescued the repression of mesendoderm differentiation induced by hypoxia. **A** Schematic diagram of AB2.2 mESC differentiation treated with or without CHIR under hypoxia. **B–F** Activating the Wnt/β-Catenin pathway with CHIR increased the total and nuclear β-Catenin levels in AB2.2 mESCs undergoing differentiation but had a minor effect on the cytoplasmic β-Catenin expression. **G** The expression of Wnt/β-Catenin downstream targets (Sp5 and Cdx1) was boosted by CHIR under hypoxia. **H** CHIR treatment restored T protein expression under hypoxia. T (red) was stained on differentiation day 4. Nuclei were stained with DPAI (blue). **I** CHIR treatment inhibited the expression of ectoderm markers (Pax6 and Nestin). **J** Schematic diagram of the parallel comparison of CHIR treatment effects on AB2.2 mESC differentiation under either normoxia or hypoxia. **K** CHIR treatment similarly improved the expression of mesendoderm markers (T, Eomes, Mesp1, and Gsc) in differentiating AB2.2 mESCs under both normoxia and hypoxia. **L** The ratio of T+ cells on differentiation day 4 in AB2.2 mESCs was detected by flow cytometry. **M** The ratio of Sox1+ cells on differentiation day 4 in AB2.2 mESCs was detected by flow cytometry. *, significant (*P*<0.05); ns, not significant

First, we performed 4-day mESC differentiation assays and administered IWP2 under normoxia to block the Wnt/β-Catenin pathway (Additional file 6: Fig. S6A). As a result, the Wnt/β-Catenin pathway downstream targets, Sp5 and Cdx1, were downregulated by IWP2 (Additional file 6: Fig. S6B). The expression of mesendoderm markers (T, Eomes, Mesp1, and Gsc) and T+ cell ratio were severely repressed upon IWP2 addition (Additional file 6: Fig. S6C-E), while the expression of ectoderm markers (Pax6 and Nestin) and Sox1+ cell ratio were upregulated (Additional file 6: Fig. S6D, S6F). These results suggested that IWP2 blockade of the Wnt/β-Catenin pathway under normoxia led to a differentiation outcome similar to hypoxia. Next, we asked whether activating the Wnt/β-Catenin pathway by CHIR treatment could rescue mesendoderm differentiation repression under hypoxia. Unlike the differentiation in Additional file 1: Fig. S1E, CHIR here was added on differentiation days 0–4 (Fig. 4A). Upon CHIR treatment, total and nuclear β-Catenin levels were markedly increased, which demonstrated that the Wnt/β-Catenin pathway was successfully activated (Fig. 4B–F). In addition, Sp5 and Cdx1 were upregulated (Fig. 4G). By performing differentiation assays, we found that T expression was significantly upregulated with the CHIR treatment (Fig. 4H), while the ectoderm markers (Pax6 and Nestin) were downregulated (Fig. 4I). Moreover, to test whether CHIR treatment could fully restore the mesendoderm differentiation under hypoxia back to the level under normoxia, we performed differentiation assays with CHIR treatment under both normoxia and hypoxia, respectively (Fig. 4J). The mesendoderm differentiation with CHIR treatment under hypoxia was remarkably improved compared to the DMSO control group under both normoxia and hypoxia, and reached the same level as the CHIR treatment group under normoxia, as indicated by the expression of mesendoderm markers (Fig. 4K) and T+ cell ratios (Fig. 4L). The ectoderm differentiation was strongly repressed by the CHIR treatment under both normoxia and hypoxia as indicated by the Sox1+ cell ratios (Fig. 4M). In summary, activating the Wnt/β-Catenin pathway fully rescued the mesendoderm differentiation that was suppressed by hypoxia, which indicated that hypoxia regulated the mesendoderm differentiation through the Wnt/β-Catenin pathway.

**Fig. 5.**
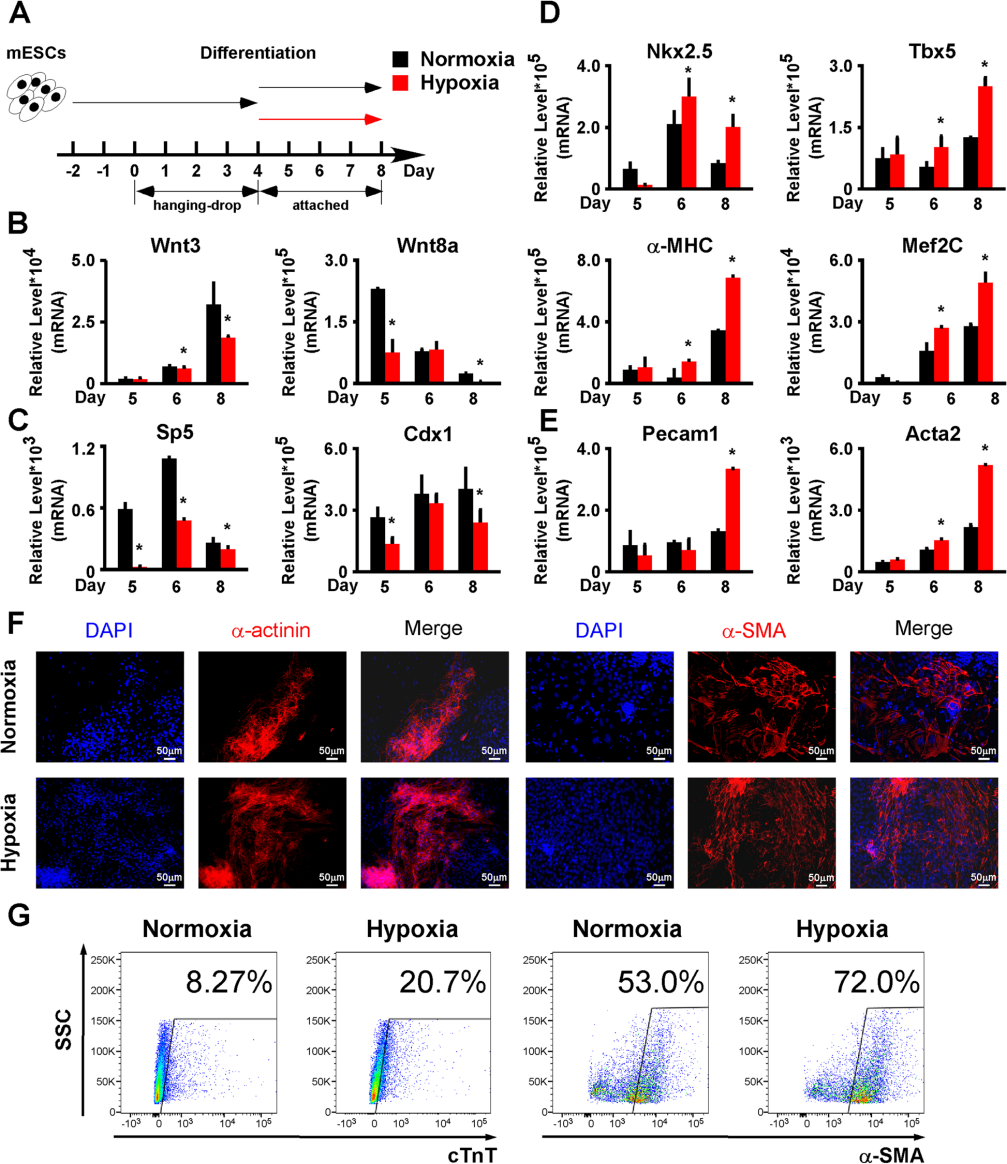
Hypoxia treatment after the mesendoderm promoted the differentiation of the mesendoderm-derived lineages. **A** Schematic diagram of the 8-day AB2.2 mESC differentiation that consisted of a 4-day differentiation under normoxia and a following 4-day differentiation under either normoxia or hypoxia. **B** Wnt3, Wnt8a, **C** Sp5, and Cdx1 were all significantly repressed by hypoxia. **D** Cardiac markers (Tbx5, Mef2C, Nkx2-5, and α-MHC) were significantly upregulated by hypoxia. **E** Pecam1 and Acta2 were significantly upregulated by hypoxia. **F** By immunostaining on differentiation day 8, the expression of α-Actinin (red) was shown to be enhanced. Nuclei were stained with DPAI (blue). **G** By immunostaining on differentiation day 8, the expression of α-SMA (red) was shown to be enhanced. Nuclei were stained with DPAI (blue). **H** The ratios of cTnT+ and α-SMA+ cells on differentiation day 8 in AB2.2 mESCs were detected by flow cytometry. *, Significant (*P*<0.05)

The activation of the Wnt/β-Catenin pathway is required for mesendoderm differentiation but its immediate repression after mesendoderm is developed is also required to enable further differentiation of mesendoderm-derived lineages, such as cardiomyocytes, endothelial, and smooth muscle cells [29–31]. As we noticed that hypoxia inhibited the Wnt/β-Catenin pathway, we asked if hypoxia treatment after mesendoderm developed could promote the differentiation of the mesendoderm-derived lineages. We performed eight-day differentiations on AB2.2 mESCs: for the first 4 days, the differentiating cells were cultured under normoxia to ensure proper mesendoderm differentiation; for the rest 4 days, the cells were classified into two groups and subjected to normoxia and hypoxia treatments, respectively (Fig. 5A). Wnt3, Wnt8a, Sp5, and Cdx1 were all significantly repressed by hypoxia, indicating that the Wnt/β-Catenin pathway was inhibited by hypoxia (Fig. 5B,C). The cardiac markers (Tbx5, Mef2C, Nkx2-5, and α-MHC), endothelial marker (Pecam1), and smooth muscle marker (Acta2) were all significantly upregulated (Fig. 5D,E). By immunostaining, we observed increased expression of structural proteins of cardiomyocytes (α-Actinin) and smooth muscle (α-SMA) (Fig. 5F,G). Flow cytometry results also showed that both the ratios of cTnT+ (a cardiac marker) and α-SMA+ cells were significantly upregulated (Fig. 5H). These results suggested that hypoxia treatment after mesendoderm was developed promoted the differentiation of the mesendoderm downstream lineages by suppressing the Wnt/β-Catenin pathway.

### Hif-1α knockdown suppressed the mesendoderm differentiation

**Fig. 6.**
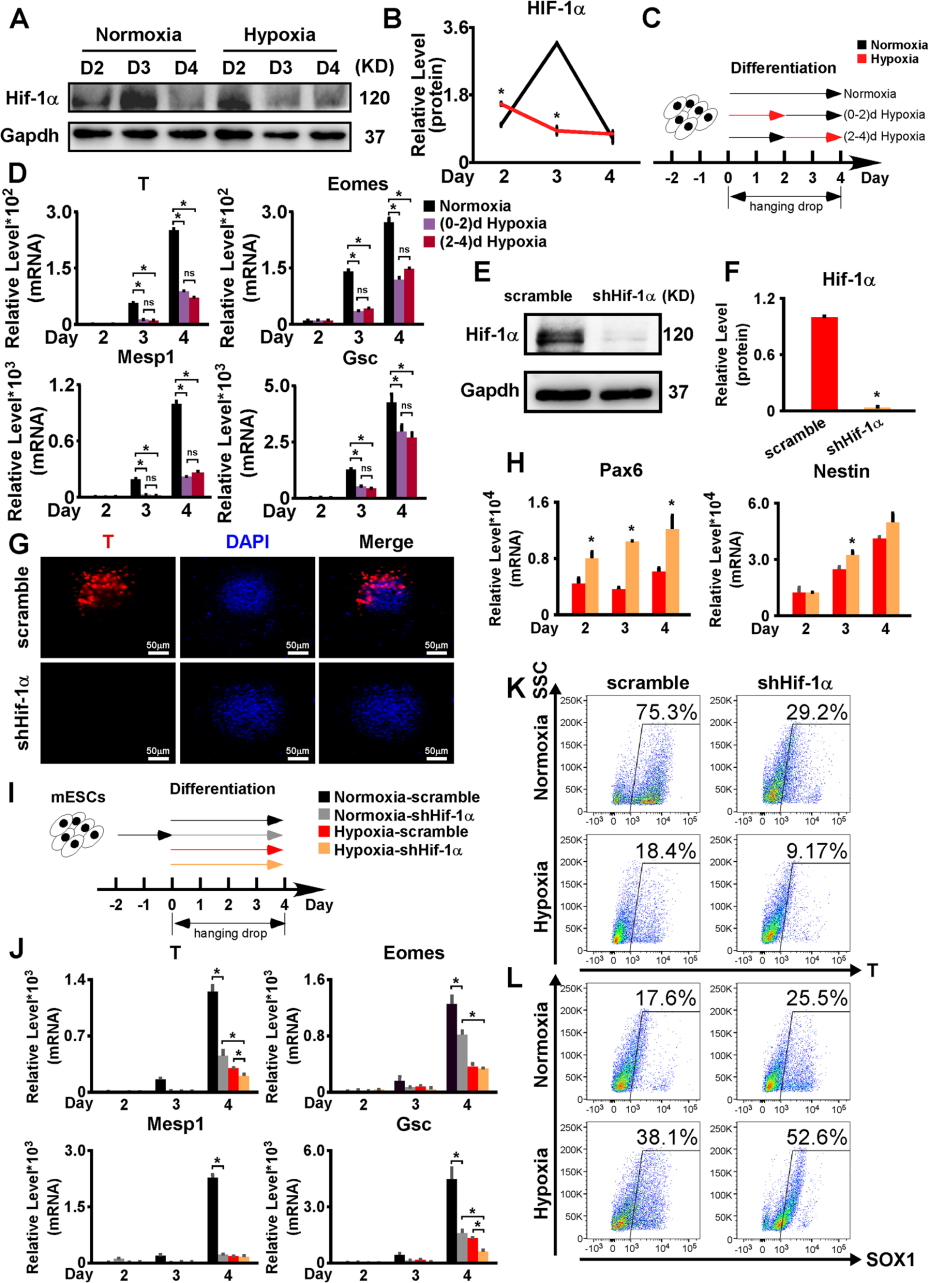
Hif-1α knockdown repressed the mesendoderm differentiation and promoted the ectoderm differentiation. **A, B** The expression patterns of Hif-1α in differentiating AB2.2 cells under normoxia and hypoxia. **C** Schematic diagram of AB2.2 mESC differentiation treated with normoxia and hypoxia on differentiation days 0–2 and 2–4, respectively. **D** The expression of mesendoderm markers (T, Eomes, Mesp1, and Gsc) was repressed to the same extent by hypoxia on differentiation days 0–2 and 2–4. **E, F** Hif-1α knockdown was verified by western blotting analysis under hypoxia. **G** Hif-1α knockdown inhibited T protein expression under hypoxia. T (red) was stained on differentiation day 4. Nuclei were stained with DPAI (blue). **H** Hif-1α knockdown promoted the expression of ectoderm markers (Pax6 and Nestin). **I** Schematic diagram of the parallel comparison of Hif-1α knockdown effects on AB2.2 mESC differentiation under either normoxic or hypoxic conditions. **J** Hif-1α knockdown inhibited the expression of mesendoderm markers (T, Eomes, Mesp1, and Gsc) on differentiation day 4 under both normoxia and hypoxia, with the effect more obvious under normoxia. The ratios of **K** T+ and **L** Sox1+ cells on day 4 of the differentiation assays in **I** were detected by flow cytometry. scramble, AB2.2/scramble cells; shHif-1α, AB2.2/shHif-1α cells; *, significant (*P*<0.05); ns, not significant

As hypoxia suppressed mesendoderm differentiation, we asked whether this suppression was Hif-1α-dependent. We first determined the expression patterns of Hif-1α in the AB2.2 mESC differentiation under normoxia and hypoxia. Under normoxia, the expression of Hif-1α was notable and reached its peak on day 3. However, under hypoxia, the peak of Hif-1α shifted to earlier than day 2, with its expression staying at a relatively low level thereafter (Fig. 6A,B). This result agreed with a previously reported pattern of Hif-1α under long-term hypoxia [49]. As PHD family members, especially PHD2, had been known as the essential enzymes mediating Hif-1α degradation, we detected the expression patterns of Egln1, the PHD2 coding gene, under both normoxia and hypoxia. The expression of Egln1 was relatively low on day 2 under normoxia and thereafter Egln1 was gradually upregulated and thus led to the drop of Hif-1α. As to hypoxia, Egln1 maintained a relatively high level on days 2–4 (Additional file 7: Fig. S7A). In general, the pattern of Egln1 was inversely correlated with Hif-1α but there was one day delay for Hif-1α to respond to the Egln1 changes, which suggested the Hif-1α changes during mESC differentiation might be at least partially due to Egln1 level changes. Next, we asked if the forward shift of Hif-1α was associated with the repressive effect caused by hypoxia. To address this, we performed differentiation assays with hypoxia treatment on day 0–2 and 2–4, respectively, to induce Hif-1α expression at different times (Fig. 6C). The levels of mesendoderm markers of these two groups were very close and both significantly lower than the normoxia control group (Fig. 6D).

We then constructed the Hif-1α knockdown (shHif-1α) and scramble control (scramble) AB2.2 cell lines and verified them by western blotting under hypoxia (Fig. 6E,F). Next, we assayed these two cell lines for 4-day differentiations under hypoxia. T expression was even further downregulated under hypoxia (Fig. 6G), while the expression of ectoderm markers (Pax6 and Nestin) was upregulated (Fig. 6H). Moreover, we also tested the effect of Hif-1α knockdown under both normoxia and hypoxia in parallel (Fig. 6I). The expression of mesendoderm markers (T, Eomes, Mesp1, and Gsc) and T+ cell ratios were downregulated by Hif-1α knockdown under both conditions, implying that the loss of Hif-1α impeded the mesendoderm differentiation regardless of normoxia or hypoxia. Strikingly, by comparing the Hif-1α knockdown under normoxia and hypoxia, we found that hypoxia still significantly repressed the mesendoderm differentiation under the circumstance of the Hif-1α loss (Fig. 6J,K). On the contrary, the Sox1+ cell ratio was upregulated with Hif-1α knockdown under both conditions, implying that Hif-1α knockdown might promote ectoderm differentiation (Fig. 6L).

### Hif-1α overexpression promoted the mesendoderm differentiation

**Fig. 7.**
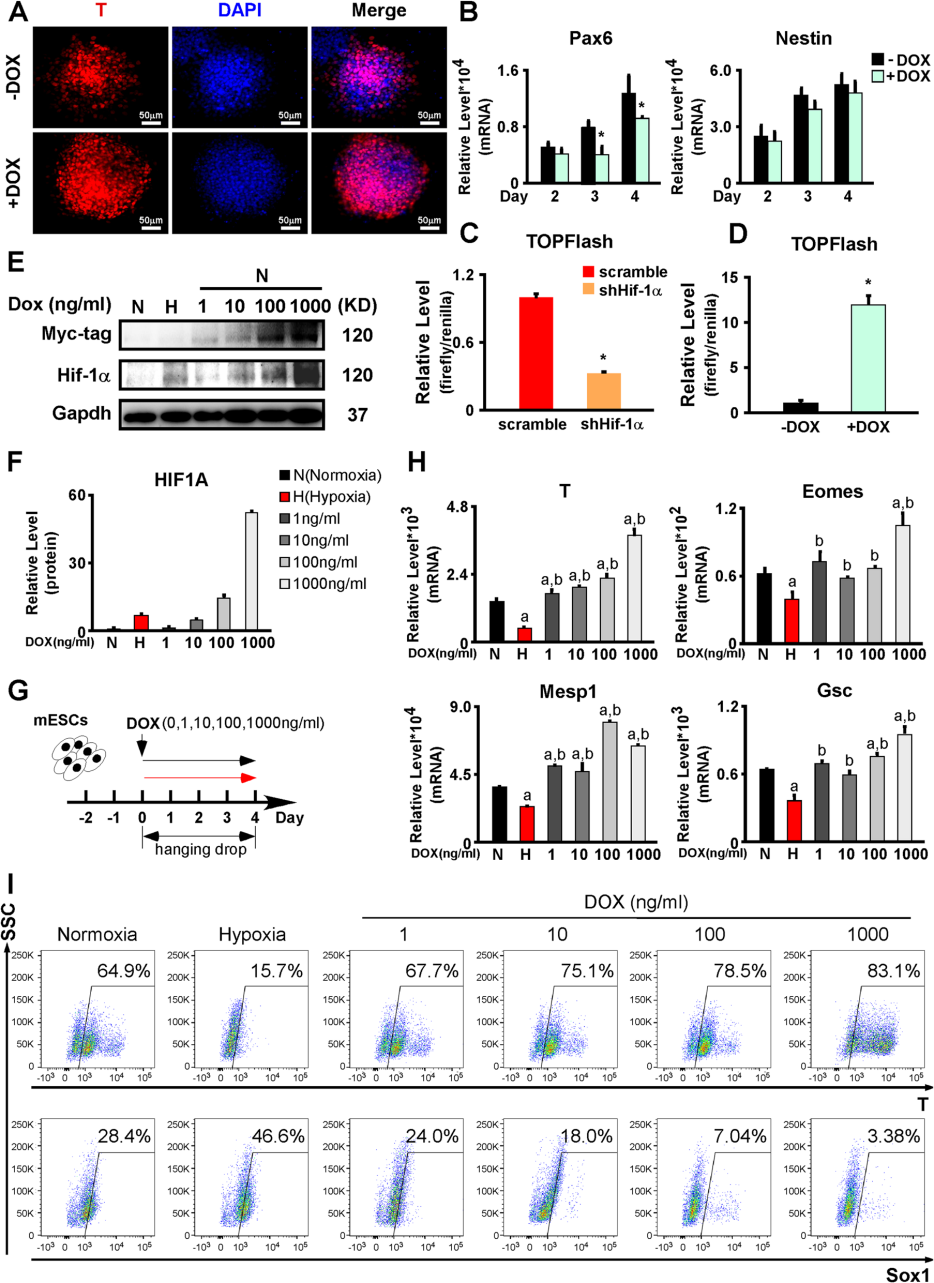
Hif-1α overexpression promoted the mesendoderm differentiation but repressed the ectoderm differentiation. **A** Hif-1α overexpression upregulated T protein expression under normoxia. T (red) was stained on differentiation day 4. Nuclei were stained with DPAI (blue). **B** Hif-1α overexpression induced by Dox supplementation repressed the expression of ectoderm markers (Pax6 and Nestin), although the difference in Nestin levels was not significant. **C** TOPFlash assays showed that Hif-1α knockdown significantly repressed the Wnt/β-Catenin pathway activation. **D** TOPFlash assays showed that Hif-1α overexpression significantly promoted the Wnt/β-Catenin pathway activation. **E, F** The expressions of Hif-1α in Hif-1α-iOE AB2.2 mESCs treated with 1, 10, 100, and 1000 ng/mL Dox, respectively. Normoxic and hypoxic cultures without dox treatment were used as controls. **G** Schematic diagram of Hif-1α-iOE AB2.2 mESC differentiation treated with 1, 10, 100, and 1000 ng/mL Dox, respectively. Normoxic and hypoxic cultures without dox treatment were used as controls. **H** The expression changes of mesendoderm markers (T, Eomes, Mesp1, and Gsc) on day 4 of the differentiation described in **G**. **I** The ratios of T+ and Sox1+ cells on day 4 of the differentiation assays in **G** were detected by flow cytometry. scramble, AB2.2/scramble cells; shHif-1α, AB2.2/shHif-1α cells; DOX, doxycycline; *, significant (*P*<0.05)

We also studied the effect of Hif-1α overexpression by differentiating doxycycline (Dox)-inducible Hif-1α-overexpressing (Hif-1α-iOE) AB2.2 cells. Initially, the Hif-1α overexpression was triggered by supplying them with 1000 ng/mL Dox. The Hif-1α overexpression was verified by western blotting with anti-Myc-tag and anti-Hif-1α antibodies (Additional file 7: Fig. S7B). For differentiation, all mesendoderm markers were significantly upregulated (Additional file 7: Fig. S7C, Fig. 7A), while the ectoderm markers were downregulated (Fig. 7B). These results indicated that Hif-1α might promote mesendoderm differentiation and repress ectoderm differentiation, in contrast to the effects of hypoxia.

Next, we wanted to determine whether Hif-1α affected the Wnt/β-Catenin pathway. We performed TOPFlash assays with Hif-1α knockdown under hypoxia and found that Hif-1α knockdown remarkably repressed the Wnt/β-Catenin pathway (Fig. 7C). Consistent with this finding, the expression of Wnt3, Wnt8a, Sp5, and Cdx1 was downregulated upon Hif-1α knockdown (Additional file 7: Fig. S7D). When Hif-1α was overexpressed, the TOPFlash activity was significantly elevated (Fig. 7D). The expression of Wnt3, Wnt8a, Sp5, and Cdx1 was upregulated by Hif-1α overexpression (Additional file 7: Fig. S7E). These results suggested that Hif-1α promoted the Wnt/β-Catenin pathway.

We then examined the effects of Hif-1α overexpression at different levels, which was achieved by supplying Hif-1α-iOE AB2.2 cells with Dox at a series of concentrations (1, 10, 100, and 1000 ng/mL) under normoxia. By western blots, we found that the levels of Hif-1α overexpression were positively correlated with the Dox concentrations. Among that, the Hif-1α overexpression induced by 10 ng/mL Dox resembled the level of endogenous Hif-1α upregulation under 2% hypoxia (Fig. 7E,F). We also tested the expressions of Hif-1α target genes (Pgk1, Lhda, Egln1, and Vegfa) in these groups. Compared to normoxia, hypoxia only treatment significantly promoted the expressions of Hif-1α target genes. Consistently, the Hif-1α target genes were all positively correlated with Dox concentrations and Hif-1α levels. It is worth noting that there was no significant difference between the hypoxia group and the 10 ng/mL Dox group, which induced a similar extent of Hif-1α overexpression with hypoxia, implying that the upregulations of Hif-1α target genes under hypoxia might be attributed to the Hif-1α levels (Additional file 7: Fig. S7F).

Subsequently, we performed in vitro differentiation on Hif-1α-iOE AB2.2 cells and induced Hif-1α overexpression using the same Dox concentration set as Fig. 7E under normoxia. Normoxic and hypoxic cultures without dox treatment were used as controls (Fig. 7G). By RT-qPCR, all mesendoderm markers were significantly upregulated in the Dox group compared to the hypoxia group (Fig. 7H,I). When compared to the normoxia group, T and Mesp1 showed significant increases in all Dox groups, while Gsc and Eomes started to display significant upregulation when Dox concentrations reached 100 ng/mL and 1000 ng/mL, respectively (Fig. 7H,I). On the contrary, Sox1+ cell ratios were inversely correlated with Dox concentrations. Dox-treated groups all exerted decreased Sox1+ cells compared to both normoxia and hypoxia groups (Fig. 7I).

### Hif-1α was a fine-tune factor for the effects of hypoxia on mESC differentiation

To compare the genes regulated by hypoxia and Hif-1α, in addition to RNA-seq of AB2.2 mESCs on differentiation day 4 under normoxia and hypoxia, we also performed RNA-seq of Hif-1α-iOE AB2.2 cells on differentiation day 4, which included a non-Dox treatment control group and 10 ng/mL Dox-treated group, aiming at mimicking the endogenous Hif-1α upregulation caused by hypoxia. As a result, Hif-1α overexpression caused only 43 DEGs (38 upregulated and 5 downregulated) using the cutoff of |LogFC| > 1 and the adjusted *P*-value < 0.05 (Additional file 8: Fig. S8A). GO analysis on the upregulated DEGs suggested that development (especially mesendoderm development), transcription, cell-cell signaling, and Wnt signaling-related processes were significantly promoted (Additional file 8: Fig. S8C). As the downregulated DEGs were too few, no significantly suppressed GO term was enriched. The GO results were consistent with our previous findings that Hif-1α promoted mesendoderm development and the Wnt signaling.

Apart from this, we performed an RNA-seq of shHif-1α AB2.2 cells that were differentiated under normoxia and hypoxia, respectively, on differentiation day 4, aiming at investigating the effects of hypoxia on the cells lacking Hif-1α. Compared to normoxia, the Hif-1α knockdown cells under hypoxia had 939 DEGs (575 upregulated and 364 downregulated) using the cutoff of |LogFC| > 1 and the adjusted *P*-value < 0.05 (Additional file 8: Fig. S8B). It should be noted that wildtype AB2.2 cells differentiated under hypoxia generated 282 DEGs when compared to normoxia, and Hif-1α overexpression induced by 10 ng/mL Dox treatment exerted 43 DEGs. These results implied that hypoxia lacking Hif-1α could cause a wider range of gene changes than hypoxia with endogenous Hif-1α expression, implying the regulatory roles of Hif-1α. Meanwhile, although Hif-1α overexpression only caused 43 DEGs, it might also cause “mild” changes in other genes so that it could fine-tune the effect of hypoxia.

**Fig. 8.**
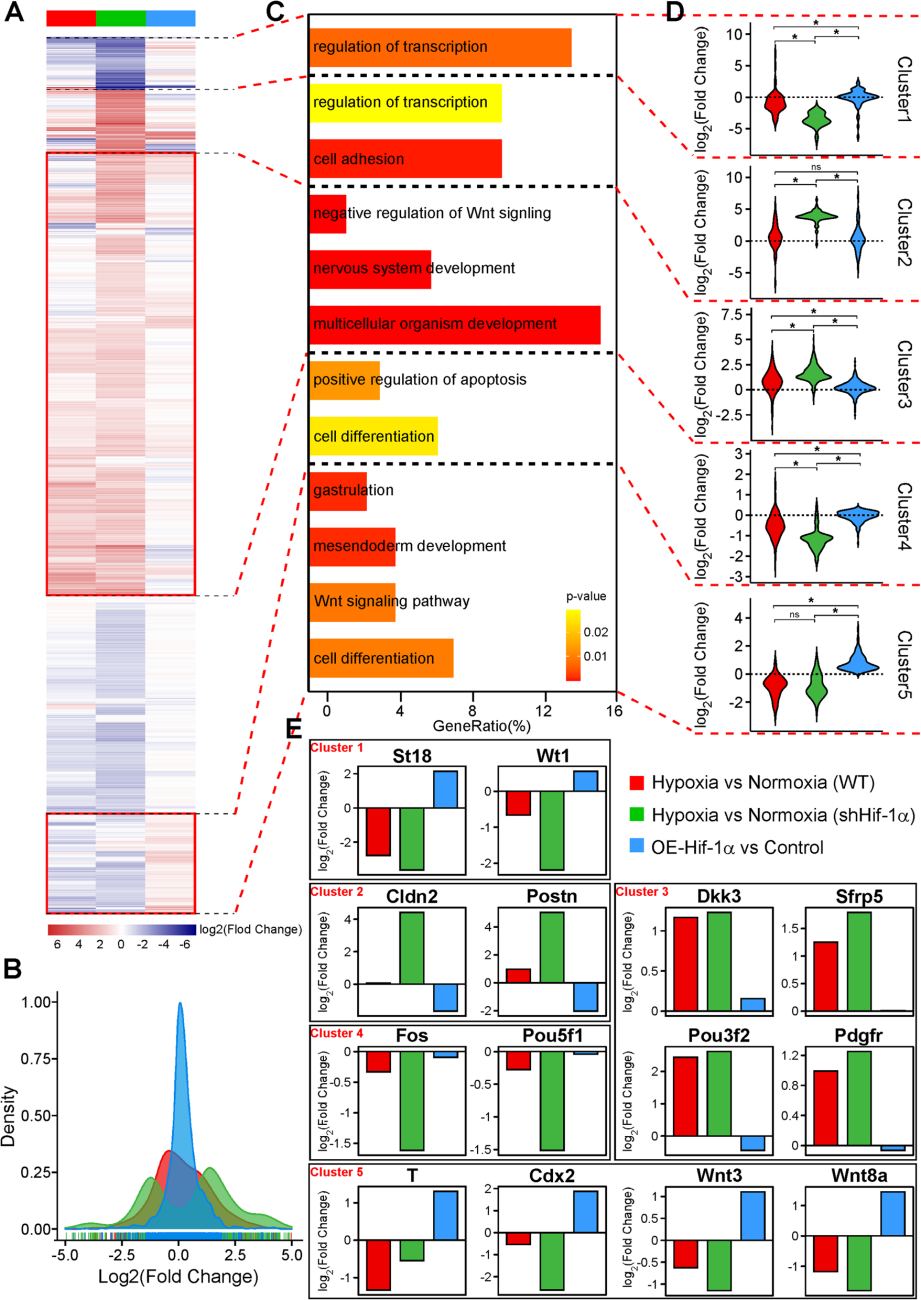
Hif-1α was a fine-tune factor for the effects of hypoxia on mESC differentiation. **A** The heatmap of logFC values of 1157 merged DEGs in the HvsN_WT, HvsN_shHif-1α, and Hif-1α-OE_vs_Con groups. All DEGs were classified into 5 clusters. **B** The distributions of LogFC values in the three groups. **C** The GO terms of the genes in each cluster in **A**. **D** The violin plots of logFC values in each cluster. **E** The level changes of representative genes in each cluster. HvsN_WT, wildtype AB2.2 cells on differentiation day 4 under hypoxia versus normoxia; HvsN_shHif-1α, shHif-1α AB2.2 cells on differentiation day 4 under hypoxia versus normoxia; Hif-1α-OE_vs_Con, Hif-1α-iOE AB2.2 cells treated with 10 ng/mL Dox versus non-Dox control on differentiation day 4; *, significant (*P*<0.05)

To test the above hypothesis, we integrated and analyzed the RNA-seq data of wildtype AB2.2 cells on differentiation day 4 under hypoxia versus normoxia (HvsN_WT), shHif-1α AB2.2 cells on differentiation day 4 under hypoxia versus normoxia (HvsN_shHif-1α), and Hif-1α-iOE AB2.2 cells treated with 10 ng/mL Dox versus non-Dox control on differentiation day 4 (Hif-1α-OE_vs_Con). We merged the DEGs from these three groups and obtained 1157 genes in total. We first plotted a heatmap with the LogFC value of these genes in each group and identified five gene clusters (Fig. 8A). The distributions of LogFC values indicated that the overall gene change extents caused by Hif-1α overexpression were the lowest among the three groups, while the HvsN_shHif-1α group displayed the largest overall LogFC range. The HvsN_WT group, which could be influenced by both Hif-1α and non-Hif-1α hypoxia factors, showed middle-level gene changes (Fig. 8B). Likewise, the logFC of the HvsN_WT group was between the HvsN_shHif-1α and Hif-1α-OE_vs_Con groups in all five clusters (Fig. 8D). Next, we studied the genes within each cluster. Cluster 1 genes were downregulated most in the HvsN_shHif-1α group and little regulated in the other two groups (Fig. 8A, D). Go analysis indicated that cluster 1 genes were involved in transcription regulation, which included St18 and Wt1 (Fig. 8C, E). Cluster 2 genes were especially upregulated in the HvsN_shHif-1α group (Fig. 8A, D). Such genes regulated transcription and cell adhesion, which included Cldn2 and Postn (Fig. 8C, E). The genes in clusters 1 and 2 were dramatically altered in the HvsN_shHif-1α group but not in the HvsN_WT and Hif-1α-OE_vs_Con groups (Fig. 8A, D), also indicating that hypoxia might rely on Hif-1α to reduce the gene vibrations caused by non- Hif-1α hypoxia factors. Cluster 3 genes were upregulated in the HvsN_WT and HvsN_shHif-1α groups, but they were little changed in the Hif-1α-OE_vs_Con group (Fig. 8A, D). The genes in this cluster were involved in the negative regulation of Wnt signaling and development (especially nervous system development) (Fig. 8C). Among the genes in cluster 3, Dkk3 and Sfrp5, negative regulators for the Wnt signaling, were upregulated in the HvsN_WT and HvsN_shHif-1α groups, but little affected in the Hif-1α-OE_vs_Con group; Pou3f2, a neural lineage factor, and Pdgfr, a regulator for multi-lineages, were upregulated in the HvsN_WT and HvsN_shHif-1α groups, but slightly repressed in the Hif-1α-OE_vs_Con group (Fig. 8E). These results implied that the Wnt signaling and ectoderm lineage regulators in cluster 3 might be Hif-1α independent. Cluster 4 genes were mostly repressed in the HvsN_WT and HvsN_shHif-1α groups, but little altered in the Hif-1α-OE_vs_Con group (Fig. 8A, D). Such genes were involved in the regulation of apoptosis and cell differentiation, which includes Fos and Pou5f1 (Oct4) (Fig. 8C, E). Considering that apoptosis was repressed by hypoxia (Additional file 8: Fig. S8D), apoptosis might be mainly regulated by hypoxia in a Hif-1α-independent manner. The distributions of LogFC values in clusters 3 and 4 were very near to zero for the Hif-1α-OE_vs_Con group but of similar trends for the HvsN_WT and HvsN_shHif-1α groups, also suggesting that Hif-1α had limited influences on the genes in the clusters 3 and 4 (Fig. 8D). Cluster 5 genes mainly regulated gastrulation, mesendoderm development, Wnt signaling, and cell differentiation (Fig. 8C). These genes were inhibited in the HvsN_WT and HvsN_shHif-1α groups but upregulated in the Hif-1α-OE_vs_Con group. T, Cdx2, Wnt3, and Wnt8a were the representative genes in this cluster. Taking together, the regulation from Hif-1α overexpression, which mimicked the endogenous Hif-1α upregulation caused by hypoxia, was relatively weaker and more selective, implying that Hif-1α was a fine-tune factor under hypoxia.

## Discussion

**Fig. 9.**
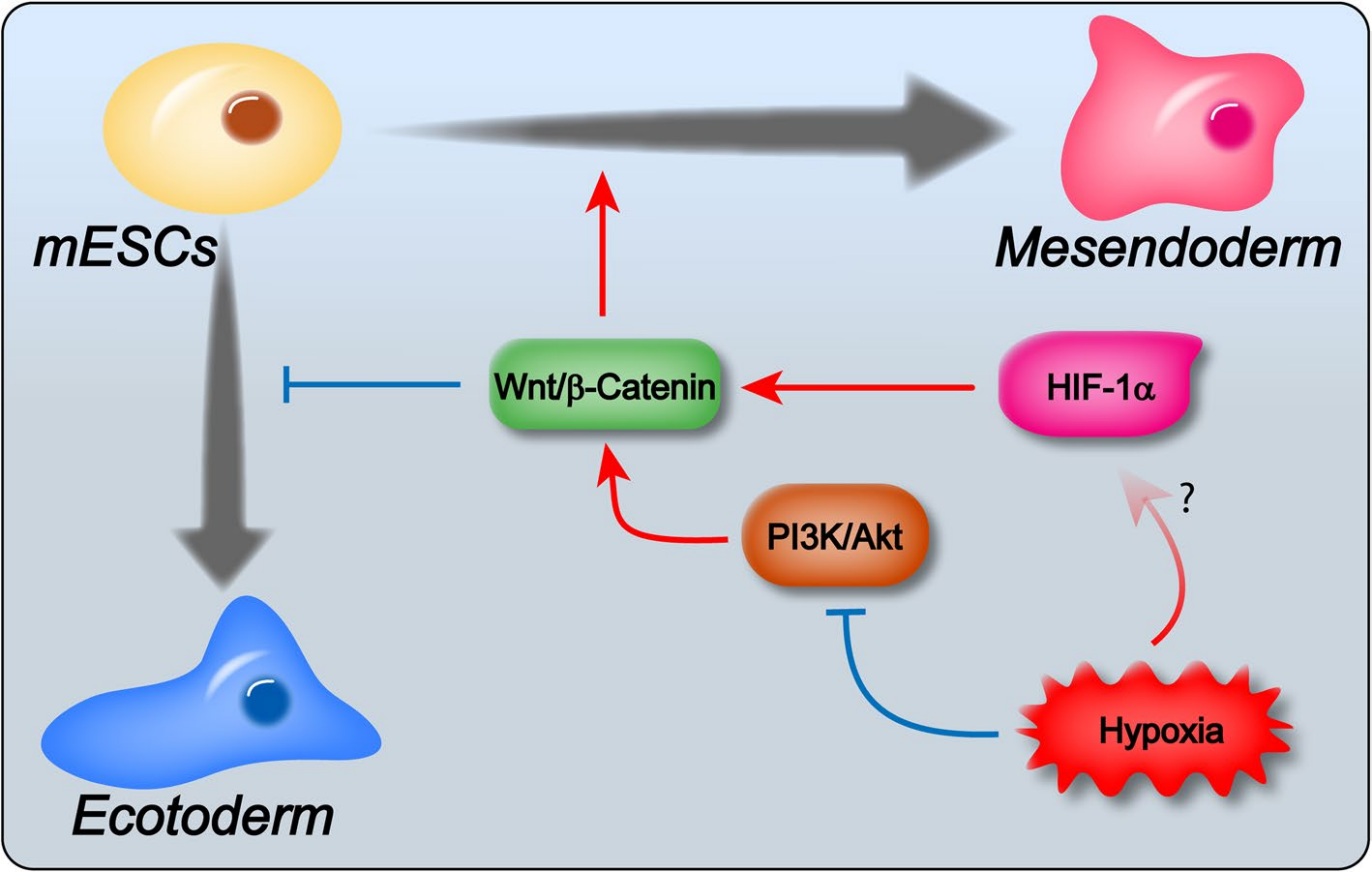
A working model of hypoxia and Hif-1α on mESC differentiation. Hypoxia suppressed the mesendoderm differentiation and promoted the ectoderm differentiation of mESCs by inhibiting the Wnt/β-Catenin pathway. The inhibition of the Wnt/β-Catenin pathway was through the PI3K/Akt/GSK3β axis. Hif-1α, on the contrary, promoted the mesendoderm differentiation and suppressed the ectoderm differentiation, which was at least partially through the Wnt/β-Catenin pathway as well

Here we studied the effects of hypoxia and Hif-1α on mESC differentiation. The mesendoderm differentiation of mESCs was suppressed by hypoxia treatment but promoted by Hif-1α. On the contrary, the ectoderm differentiation was promoted by hypoxia but suppressed by Hif-1α. The effects of hypoxia and Hif-1α relied on manipulating the Wnt/β-Catenin pathway. Under hypoxia, the Wnt/β-Catenin pathway was suppressed as a result of the PI3K/Akt/GSK3β axis repression. In contrast, Hif-1α overexpression promoted the Wnt/β-Catenin pathway, while its knockdown repressed the Wnt/β-Catenin pathway. Hif-1α acted as a fine-tune factor for the effects of hypoxia (Fig. 9).

Hypoxia occurs when the oxygen supply through passive diffusion cannot meet the demand of embryo growth in the uterus, which was typically ≤2% O_2_ [1]. Thus, the “hypoxia” condition we used in this study was 2% O_2_, which resembled the oxygen concentration during development and was termed relative to the ambient air. Upon hypoxia during embryo development, the development of circulation and hematopoietic systems is triggered to provide the embryo with sufficient oxygen and nutrient supplies through blood circulation. Multiple reports have documented the promotive role of hypoxia in angiogenesis and hematopoiesis [50, 51]. As both blood vessels and hematopoietic systems belong to mesendoderm-derived lineages, it is logical to speculate that the mesendoderm specification is also promoted by hypoxia. Nonetheless, we here discovered that the mesendoderm differentiation of mESCs was severely impaired by hypoxia. This was consistent with a recent finding that T, an essential mesendoderm marker, was gradually downregulated by hypoxia in an oxygen concentration-dependent manner [10]. However, another report also showed that T in differentiating ESCs was upregulated by hypoxia when the hypoxia was performed at a relatively late stage, while hypoxia starting from the beginning of mESC differentiation truncated the mesoderm formation [16]. This agreed with our observation that hypoxia suppressed the mesendoderm differentiation from mESCs, while promoting the downstream lineage differentiation from mesendoderm. In other words, the effects of hypoxia on development are highly stage and cell type sensitive. As the oxygen concentration in the uterus is dynamic and oxygen within different parts of embryos varies, the oxygen supply for all embryo cells is always changing and thus might serve as an important factor in regulating embryo development.

Furthermore, previous studies have implied the important role of hypoxia in the survival and differentiation of neural stem cells [52, 53]. The ectoderm region of mouse embryos was reported to be hypoxic at the E8.5-16.5 stage [54]. Consistently, we found that hypoxia promoted the ectoderm differentiation as suggested by the upregulations of Sox1, Pax6, and Nestin and RNA-seq data. Particularly, the effect of hypoxia on the ectoderm differentiation seems to be prior to epiblast formation as the Sox1+ cell ratio was little affected by hypoxia in the differentiation of EpiLCs (Additional file 1: Fig. S1I). Moreover, we noticed that the size of embryoid bodies (EB) in the hypoxia group was relatively smaller than the normoxia group (Fig. 1C, E, H). This encouraged us to determine whether the cell cycle and apoptosis processes were affected by hypoxia. We performed GSEA of these two processes and found that the cell cycle and apoptosis were both repressed by hypoxia (Additional file 8: Fig. S8D-E). Thus, the smaller EB under hypoxia might be due to the repressed cell cycle. As we have shown that hypoxia suppressed the Wnt/β-Catenin and PI3K/Akt pathways, we thought that these two pathways might at least partially mediate the change in the cell cycle, agreeing with their established roles in the cell cycle [55–57].

Hif-1α has been well known as a central regulator in hypoxia response [58]. Previous studies mostly showed synergetic functions of hypoxia and Hif-1α, with few studies, however, displaying Hif-1α-independent hypoxia effects [59, 60]. Moreover, some studies simply concluded and inferred the effect of hypoxia from the results of Hif-1α. Surprisingly, we found that in contrast to hypoxia, Hif-1α augmented mesendoderm differentiation. This agreed with prior reports that Hif-1α promoted mesendoderm differentiation and loss of Hif-1α led to heart abnormalities [5, 61]. We first checked the expression patterns of Hif-1α along the mESC differentiation under normoxia and hypoxia. In line with previous reports, Hif-1α was immediately boosted by hypoxia and then dropped back and maintained at a relatively low level. Intriguingly, Hif-1α under normoxia displayed a peak type expression, with the peak on day 3. The overall expressions of Hif-1α were lower under hypoxia versus normoxia for differentiation days 2-4 and its peak on day 3 was diminished by hypoxia as well (Fig. 6A,B). This agreed with previous findings that long-term hypoxia would suppress the expression of Hif-1α [49]. Next, we tested the effects of Hif-1α knockdown under normoxia and hypoxia in parallel and found that Hif-1α knockdown under both normoxia and hypoxia suppressed the mesendoderm differentiation and promoted the ectoderm differentiation, with the changes under normoxia being more obvious than hypoxia ones (Fig. 6I–L), which matched the relative levels of Hif-1α under both conditions (Fig. 6A,B). Moreover, by comparing the differentiation of Hif-1α knockdown mESCs under normoxia and hypoxia, we noticed that hypoxia still suppressed the mesendoderm differentiation and promoted the ectoderm differentiation even though Hif-1α was knockdown, implying that Hif-1α was not necessary for the effects of hypoxia. Consistently, by overexpressing Hif-1α, we found that the mesendoderm and ectoderm differentiations were promoted and suppressed, respectively. By analyzing RNA-seq data, we noticed that the endogenous Hif-1α upregulation under hypoxia itself only caused limited numbers of DEGs, and its regulations on mESC differentiation-related genes were relatively milder compared to hypoxia. Moreover, the range of genes regulated by Hif-1α was relatively narrower than hypoxia. For example, the genes in clusters 3 and 4, which were involved in the regulation of ectoderm development and apoptosis, were mainly regulated by hypoxia rather than Hif-1α. However, as to the genes in cluster 5, which regulated mesendoderm development and Wnt signaling, hypoxia and Hif-1αexerted opposite influences, consistent with the results of our differentiation assays (Fig. 8). Thus, Hif-1α might act as a fine-tune factor for the effects of hypoxia on mESC differentiation. To our best knowledge, this is the first report of the adverse functions of hypoxia and Hif-1α.

Mesendoderm differentiation is a complex process that is precisely controlled by many extra- and intracellular signaling pathways. By the pathway analysis of RNA-seq data, we found that Wnt (especially Wnt/β-Catenin) pathway and hedgehog ligand biogenesis were both significantly repressed (Fig. 2D). hedgehog (Hh) signaling was involved in the development of the brain, spinal cords, limbs, and internal organs [62]. Particularly, a recent study showed that activating the Hh signaling promoted the neuroectoderm specification [63]. We investigated the relative levels of Hh signaling-related genes and found that most Hh activation-related components, including Gli1/2 and Ptch1/2, were upregulated, though the hedgehog members (Dhh, Ihh, and Shh) were downregulated (Additional file 3: Fig. S3C). This indicated that the Hh signaling was likely activated by hypoxia, though the hedgehog ligand biogenesis was repressed. Thus, we thought the Wnt pathway rather than Hh signaling might mediate the effects of hypoxia. Furthermore, by testing the relative expressions of essential genes and GSEA, we believed that the Wnt/β-Catenin pathway functioned downstream of hypoxia in regulating mESC differentiation.

Manipulations of each step of the Wnt/β-Catenin pathway have been found to significantly affect mesendoderm differentiation [64, 65]. Gsk3β stays directly upstream of β-Catenin, whose upregulation and dephosphorylation repressed the accumulation and localization of β-Catenin [66]. CHIR is widely used as a Gsk3β inhibitor and Wnt/β-Catenin pathway activator [67]. Here we tried supplying CHIR on differentiation days 0–4 and 3–4, respectively, and found that CHIR on differentiation days 0–4 fully restored the mesendoderm differentiation (Fig. 4J–M), while the day 3–4 CHIR treatment still exerted suppressed mesendoderm differentiation compared to the normoxia (Additional file 1: Fig. S1E-G). This might suggest that the functions of hypoxia on mesendoderm differentiation and Wnt/β-Catenin started earlier than day 3. Akt, an important factor in the PI3K/Akt pathway, acted as a kinase for Gsk3β, thereby regulating the activity of the Wnt/β-Catenin pathway. Through western blotting, we found that hypoxia severely suppressed the Wnt/β-Catenin pathway likely through the Akt/Gsk3β axis. This finding was in accord with the reports that hypoxia affected cell metabolism by regulating p-Akt(S473) in a Hif-1α-independent manner in mESCs [59] and p-Akt(S473) directly phosphorylated Gsk3β(S9) [68, 69]. To validate this, we used SC79, an Akt activator, to counteract the repression of p-Akt(S473) under hypoxia. As a result, the Wnt/β-Catenin pathway, as well as the mesendoderm differentiation, was rescued, corroborating our hypothesis. Apart from the Akt/Gsk3β axis, other possible mechanisms might also mediate the effect of hypoxia on the Wnt/β-Catenin pathway. For example, casein kinase 1 (CK1) is an essential component of the β-Catenin destruction complex, which initials the destruction of β-Catenin by phosphorylating the Ser45 of β-Catenin [70]. Although CK1 was shown to phosphorylate both Hif-1α and Hif-2α to affect their activities, its role in hypoxia remains unclear [71, 72]. Thus, exploring the roles of CK1 in hypoxia and mESC differentiation might deepen our understanding of the inhibition of the Wnt/β-Catenin pathway under hypoxia, which calls for investigations in the future.

## Conclusions

Hypoxia and Hif-1α oppositely regulate the mesendoderm and ectoderm differentiation through manipulating the Wnt/β-Catenin pathway, which might contribute to our understanding of hypoxia-involved stem cell fate determination during embryogenesis.

## Methods

### Cell culture

AB2.2, R1, and HEK293T cells were kindly provided by the Stem Cell Bank, Chinese Academy of Sciences. The AB2.2 and R1 cells were cultured in KnockOut DMEM (Gibco) supplemented with 15% fetal bovine serum (ExCell), 50 U/mL penicillin (Gibco), and 50 μg/mL streptomycin (Gibco), 2 mM L-glutamine (Gibco), 1× MEM Non-Essential Amino Acids Solution (Gibco), 0.1 mM 2-Mercaptoethanol (Sigma), and 10^3^ units LIF (Millipore) on 0.1% gelatin (Sigma)-coated plates. Upon differentiation, AB2.2 and R1 cells were cultured by the hanging-drop method for 4 days, followed by another 4 days of differentiation on 0.1% gelatin-coated plates if needed. The differentiation medium, if not otherwise specified, was 75% IMDM (HyClone) and 25% F12 (HyClone) supplemented with 2 mM L-glutamine, 0.25% BSA (Sigma), 1× B-27 (Gibco), 0.5× N-2 (Gibco), 50 U/mL penicillin, 50 μg/mL streptomycin, and 0.45 mM 1-thioglycerol (Sigma). Activin A (R&D, 0.01 μg/mL) was added to the culture for the first 4 days of differentiation. The high-serum differentiation medium, which was non-lineage-prone, consisted of KnockOut DMEM supplemented with 20% fetal bovine serum, 50 U/mL penicillin and 50 μg/mL streptomycin, and 0.1 mM 2-Mercaptoethanol. The final concentration of IWP2 or CHIR99021 (CHIR) was 3 μM when used during differentiation. Epiblast like cells (EpiLCs) were induced by seeding mESCs onto 16.7 μg/mL fibronectin (Gibco)-coated 6-well plates (2×10^5^ /well) and cultured in an N2B27 medium supplemented with 20 ng/mL activin A, 12.5 ng/ml bFGF (Peprotech), and 1% KSR (Gibco) for 4 days as previously described [42, 43]. For mesendoderm differentiation, EpiLCs were cultured in an N2B27 medium supplemented with 20 ng/mL activin A and 3 μM CHIR for 24 h as previously described [44]. HEK293T cells were cultured in high-glucose DMEM (HyClone) supplemented with 10% fetal bovine serum (Clark), 50 U/mL penicillin, and 50 μg/mL streptomycin.

All cells were cultured in a regular cell culture incubator (Thermo Fisher Scientific) with ambient O_2_ and 5% CO_2_ at 37°C, except for those subjected to hypoxia treatment, which were cultured in another tri-gas cell culture incubator (Thermo Fisher Scientific) with 2% O_2_ and 5% CO_2_ at 37°C. Typically, the mESC differentiation towards mesendoderm consisted of a 4-day hanging-drop culture under hypoxia without medium change. Thus, the differentiating cells under hypoxia were kept in the hypoxic incubator without disturbance for the whole 4-day culture. As to specific culture treatments (e.g., adding CHIR on day 3), we took out one culture dish each time to narrow the hypoxia interruption time to less than 1 min for medium change and harvesting embryoid bodies.

### Plasmids and transfection

pLL4.0 and Scramble plasmids were constructed by our laboratory as described previously [73]. The shHif-1α plasmid was produced by ligating the Hif-1α shRNA coding sequence into a pLL4.0 vector. The forward strand of the shHif-1α coding sequence is 5′-TGCTGTTGATCTTATAATGATTCAAGAGATCATTATAAGATCAACAGCTTTTTTC-3′, and the reverse strand is 5′-TCGAGAAAAAAGCTGTTGATCTTATAATGATCTCTTGAATCATTATAAGATCAACAGCA-3′. The pcDNA3 mHIF-1α MYC (P402A/P577A/N813A) and pCW57-MCS1-2A-MCS2 plasmids were purchased from Addgene. The pCW57-Hif-1α plasmid, a doxycycline-inducible oxygen-resistant Hif-1α overexpression plasmid, was developed by ligating the Hif-1α expression cassette of the pcDNA3 mHIF-1α MYC (P402A/P577A/N813A) plasmid to the pCW57-MCS1-2A-MCS2 vector at the BamHI site.

Plasmid transfection was performed using PolyJet (SignaGen). Cells were seeded onto cell culture dishes 24 h before transfection to ensure cell confluence of between 80 and 90% at the transfections. The ratio of plasmids to PolyJet was 1:2 (μg:μL). The medium was changed 24 h posttransfection.

### Lentivirus production and mESC-based cell line construction

To produce lentivirus for mESC infection, the aforementioned lentiviral plasmids along with psPAX2 and PMD2.G plasmids were transfected into HEK293T cells at a ratio of 3:2:1 using PolyJet (SignaGen). The medium was changed 24 h posttransfection. The supernatant medium, containing lentiviruses, was collected 48 and 72 h after transfection. mESCs were infected with this lentivirus-containing supernatant during subculture. Drug selections were performed starting 96 h postinfection until stable. All constructed cell lines were verified by western blotting or RT-qPCR.

### Luciferase assays

Dual-luciferase assays were performed according to the manufacturer’s instructions (Beyotime). TOPFlash and β-Catenin overexpression plasmids were gifts from Dr. Yu Liu at the University of Houston. The PRL-TK plasmid was used as a Renilla luciferase control. All plasmids were transiently transfected into HEK293T cells. Samples were collected 48 h posttransfection, and the luciferase activity was measured immediately: Relative luciferase activity = firefly luciferase activity/Renilla luciferase activity.

### Real-time quantitative RT-PCR (RT-qPCR)

We used a total RNA isolation reagent (Biosharp) for total RNA extraction, the FastKing RT kit (Tiangen) for reverse transcription, and the Powerup SYBR master mix (Applied Biosystems) for quantitative PCR. All these experiments were performed according to the corresponding manufacturer’s instructions. Gapdh was used as an internal control. The sequences of the RT-qPCR primers are listed in Additional file 9: Table S1.

### RNA-sequencing (RNA-seq) and data analysis

Total RNA samples were sent to Anhui Microanaly Genetech Co., Ltd., for library construction and RNA-seq. The raw RNA-seq data were first trimmed and filtered by the TrimGalore software. The reads were aligned to the mouse genome (GRCm38) using HISAT2 software. Read counts were generated using StringTie software. Differentially expressed genes (DEGs) were determined by the DESeq2 package in R [74]. |LogFC|>1, and the adjusted *P*-value <0.05 was used as the cutoff criteria. Gene ontology and GSEA were performed using the clusterProfiler package [75]. All RNA-seq data are available in the GEO database (GSE171871, GSE208062).

### Western blotting analysis

Whole-cell proteins were extracted with the EASYpack protease inhibitors (Roche) and phosphatase inhibitor-containing cell lysis buffer (Beyotime). Separate cytoplasmic and nuclear proteins were extracted using a nuclear and cytoplasmic protein extraction kit (Beyotime) according to the manufacturer’s instructions. Protein concentrations were measured using a BCA protein assay kit (Biosharp) and adjusted to be at the same concentration for SDS-PAGE electrophoresis and immunoblotting, which were conducted as previously described [73]. The following antibodies were used: anti-β-Catenin mAb (1:1000, Cell Signaling, Catalog No. 8480), anti-Gapdh pAb (1:2000, Biosharp, Catalog No. BL006B), anti-Histone H3 mAb (1:5000, HuaBio, Catalog No. EM30605), anti-p-Gsk3β(S9) mAb (1:1000, Cell Signaling, Catalog No. 5558), anti-Gsk3β mAb (1:1000, Invitrogen, Catalog No. MA5-15109), anti-p-Akt(S473) mAb (1:1000, Invitrogen, Catalog No. MA1-20325), anti-Akt Pan pAb (1:1000, Invitrogen, Catalog No. 44-609G), anti-p-mTOR(S2448) mAb (1:1000, Cell Signaling, Catalog No. 5536), anti-mTOR mAb (1:1000, Cell Signaling, Catalog No. 2983), anti-Hif-1α mAb (1:1000, Cell Signaling, Catalog No. 14179), anti-Myc-Tag mAb (1:1000, Cell Signaling, Catalog No.2276), goat anti-mouse HRP secondary antibody (1:2000, Biosharp, Catalog No. BL001A), and donkey anti-rabbit HRP secondary antibody (1:2000, Invitrogen, Catalog No. 31458). Gel band intensities were quantified using ImageJ software.

### Immunostaining of cultured cells

Cell immunostaining was performed as described previously [73]. Briefly, cells were fixed with 4% PFA and then blocked with 10% normal goat serum and 0.1% Triton X-100 in PBS. The cells were then incubated with primary antibodies overnight. On the next day, the cells were incubated with the secondary antibodies for 90 min and DPAI for 5 min. The cells were kept in PBS for observation. Images were taken with a Leica DMi8 fluorescence microscope. The following antibodies were used: anti-T mAb (1:100, Abcam, Catalog No. ab209665), anti-Sarcomeric α-Actinin mAb (1:100, Abcam, Catalog No. ab9465), anti-α-SMA mAb (1:100, Santa Cruz, Catalog No. sc32251), anti-Sox1 mAb (1:100, Abcam, Cat No. ab109290), goat anti-rabbit Alex Fluor 488-conjugated IgG (1:500, Invitrogen, Catalog No. A11008), goat anti-mouse Alexa Fluor Plus 555-conjugated IgG (1:500, Invitrogen, Catalog No. A32727).

### Flow cytometry

Cultured cells were trypsinized into single cells and then briefly washed with PBS. Subsequently, the cells were fixed with 4% PFA for 15 min and incubated in 90% methanol overnight at −20 °C. The next day, the cells were re-suspended and incubated in 0.5% BSA diluted primary antibody for 1 h, with no antibody treatment as the control for flow cytometry gating. Next, the cells were incubated with secondary antibodies for 30 min. Finally, the cells were subjected to flow cytometry on a BD FACSCantoII Flow Cytometer (BD). The following antibodies were used: anti-T mAb (1:100, Abcam, Cat No. ab209665), anti-SOX1 mAb (1:100, Abcam, Cat No. ab109290), anti-Troponin T (cTnT) mAb (1:100, Invitrogen, Cat No. MA512960), anti-α-SMA mAb (1:100, Santa Cruz, Cat No. sc32251), goat anti-rabbit IgG (H+L) pAb (1:500, Invitrogen, Cat No. A-11008), goat anti-mouse IgG (H+L) pAb (1:500, Invitrogen, Cat No. A-11001).

### Statistical analysis

The RNA-seq assays and quantifications of protein expressions using Western blotting were performed with three replicates, while the RT-qPCR and luciferase assays were performed with four replicates. For two-group comparisons, two-sided Student’s *t* test was performed; for three or more groups, one-way ANOVA was performed. *P*<0.05 was considered to be statistically significant. The data are shown as the means±SD.

## Supplementary Information

**Additional file 1: Fig. S1.** Further validation of the effects of hypoxia on mESC differentiation. (**A**) Hypoxia significantly repressed the mRNA expression of endoderm markers (Sox17 and Cxcr4) in differentiating AB2.2 mESCs. (**B**) Hypoxia significantly repressed the mRNA expression of an endothelial marker (Pecam1) and a smooth muscle marker (Acta2) in differentiating AB2.2 mESCs. (**C**) Hypoxia significantly repressed the expression of mesendoderm markers (T, Eomes, Mesp1, and Gsc) in differentiating R1 mESCs. (**D**) Hypoxia significantly upregulated the mRNA expression of ectoderm markers (Pax6 and Nestin) in differentiating R1 mESCs. (**E**) Schematic diagram of AB2.2 mESC differentiation under normoxia or hypoxia with CHIR treatment on differentiation day 3-4. (**F**) In the differentiation shown in (**E**), the expression of mesendoderm markers (T, Eomes, Mesp1, and Gsc) was also repressed by hypoxia. (**G**) In the differentiation shown in (**E**), the ratios of T+ and Sox1+ cells were downregulated and upregulated, respectively, by hypoxia. (**H**) In the mesendoderm differentiation from epiblast like cells (EpiLCs), the expression of mesendoderm markers (T, Eomes, Mesp1, and Gsc) was also repressed by hypoxia. (**I**) In the mesendoderm differentiation from EpiLCs, the T+ cell ratio was downregulated, while the Sox1+ cell ratio was minorly altered. R1, the assays performed in R1 mESCs; EpiLC, the assays performed in epiblast like cells; N, normoxia; H, hypoxia; *, significant (*P*<0.05).

**Additional file 2: Fig. S2.** The effects of hypoxia on AB2.2 mESC differentiation performed with a high-serum differentiation medium. The differentiation in the high-serum differentiation medium was non-lineage-prone. In the differentiation performed with the high-serum medium, the expressions of (**A**) mesendoderm markers (T, Eomes, Mesp1, and Gsc) and (**B**) ectoderm markers (Pax6 and Nestin) were downregulated and upregulated by hypoxia, respectively. *, significant (*P*<0.05).

**Additional file 3: Fig. S3.** The supplementary analyses performed on the RNA-seq data of AB2.2 mESCs on differentiation day 4 under normoxia and hypoxia. (**A**) PCA of RNA-seq data from normoxia and hypoxia groups. The PCA included three biological repeats for each group. (**B**) The volcano plot showed the differentially expressed genes (DEGs). |Log_2_(Fold Change)|>1 and adjusted *P*-value<0.05 were used as the cutoff criteria. (**C**) The related levels of Hh signaling-related genes in AB2.2 mESCs on differentiation day 4 under hypoxia versus normoxia. (**D**) The related levels of noncanonical Wnt pathway-related genes in AB2.2 mESCs on differentiation day 4 under hypoxia versus normoxia. (**E**) GSEA showed that the noncanonical pathway activation was not uniformly regulated by hypoxia. (**F**) GSEA showed that the PI3K/Akt signaling pathway was significantly repressed by hypoxia. *, significant (*P*<0.05).

**Additional file 4: Fig. S4.** The analyses performed on the RNA-seq data of AB2.2 mESCs on differentiation day 2 under normoxia and hypoxia. (**A**) The related levels of HIF-1 signaling-related genes in AB2.2 mESCs on differentiation day 2 under hypoxia versus normoxia. (**B**) GSEA showed that the HIF-1 signaling pathway activation was significantly enhanced by hypoxia. (**C**) The related levels of Wnt/β-Catenin pathway-related genes in AB2.2 mESCs on differentiation day 2 under hypoxia versus normoxia. (**D**) GSEA showed that the Wnt/β-Catenin pathway was significantly repressed by hypoxia (**E**) GSEA showed that the PI3K/Akt signaling pathway was significantly repressed by hypoxia. *, significant (*P*<0.05).

**Additional file 5: Fig. S5.** The Wnt/β-Catenin pathway was affected by hypoxia. (**A**) The expression of canonical Wnts (Wnt3 and Wnt8a) and (**B**) Wnt/β-Catenin pathway downstream targets (Sp5 and Cdx1) was downregulated by hypoxia in AB2.2 mESCs undergoing differentiation. (**C**) The expression of canonical Wnts (Wnt3 and Wnt8a) and (**D**) Wnt/β-Catenin pathway downstream targets (Sp5 and Cdx1) was downregulated by hypoxia in R1 mESCs undergoing differentiation. (**E-G**) Expression of nuclear β-Catenin was significantly repressed, while that of cytoplasmic β-Catenin was upregulated until day 3 and downregulated on day 4 with hypoxia treatment. *, significant (*P*<0.05).

**Additional file 6: Fig. S6.** The Wnt/β-Catenin pathway and AB2.2 mESC differentiation were affected by IWP2. (**A**) Schematic diagram of mESC differentiation treated with or without IWP2 under normoxia. (**B**) The expression of Wnt/β-Catenin downstream targets (Sp5 and Cdx1) was severely inhibited by IWP2 in AB2.2 mESCs undergoing differentiation. (**C**) IWP2 repressed T protein expression (red) on differentiation day 4. Nuclei were stained with DPAI (blue). (**D**) The ratios of T+ and Sox1+ cells were downregulated and upregulated by IWP2, respectively. (**E**) IWP2 treatment significantly repressed the mRNA expression of mesendoderm markers (T, Eomes, Mesp1, and Gsc) in AB2.2 mESCs undergoing differentiation. (**F**) Inhibiting the Wnt/β-Catenin pathway by IWP2 upregulated the expression of ectoderm markers (Pax6 and Nestin). *, significant (*P*<0.05).

**Additional file 7: Fig. S7.** The supplementary data of the effects of Hif-1α on the mesendoderm differentiation and Wnt/β-Catenin pathway. (**A**) The expression patterns of Egln1 in the differentiating AB2.2 mESCs under normoxia and hypoxia. (**B**) Doxycycline (Dox)-inducible overexpression of oxygen-resistant Hif-1α was verified by western blotting analysis. A Myc tag was added to the C-terminus of Hif-1α. (**C**) The mRNA expression of mesendoderm markers (T, Eomes, Mesp1, and Gsc) in AB2.2 mESCs with or without Dox triggered Hif-1α overexpression under normoxia. (**D**) The mRNA expression of canonical Wnts (Wnt3 and Wnt8a) and Wnt/β-Catenin pathway downstream targets (Sp5 and Cdx1) in scramble control and shHif-1α AB2.2 mESCs under hypoxia. (**E**) The mRNA expression of canonical Wnts (Wnt3 and Wnt8a) and Wnt/β-Catenin pathway downstream targets (Sp5 and Cdx1) in AB2.2 mESCs with or without Dox triggered Hif-1α overexpression under normoxia. (**F**) The expression changes of the HIF-1 signaling targets (Pgk1, Ldha, Egln1, and Vegfa) in Hif-1α-iOE AB2.2 mESCs treated with 1, 10, 100, and 1000 ng/mL Dox, respectively. Normoxic and hypoxic cultures without dox treatment were used as controls. N, normoxia; H, hypoxia; a, significant (*P*<0.05) compared to the normoxia group; b, significant (*P*<0.05) compared to the hypoxia group. pCW57-Hif-1α, AB2.2/DOX-inducible overexpression of oxygen-resistant Hif-1α; scramble, AB2.2/scramble cells; shHif-1α, AB2.2/shHif-1α cells; DOX, doxycycline; *, significant (*P*<0.05).

**Additional file 8: Fig. S8.** The supplementary analyses performed on the RNA-seq data of normoxia, hypoxia, control, and Hif-1α overexpression groups. The volcano plot showed the differentially expressed genes (DEGs) in (**A**) Hif-1α-OE_vs_Con and (**B**) HvsN_shHif-1α. |Log_2_(Fold Change)|>1 and adjusted *P*-value<0.05 were used as the cutoff criteria. (**C**) The GO terms of the DEGs upregulated by Hif-1α overexpression. GSEA of (**D**) apoptosis and (**E**) cell of AB2.2 mESCs on differentiation day 4 under hypoxia versus normoxia.

**Additional file 9: Table S1.** List of primers used for RT-qPCR.

**Additional file 10.** A excel file that includes the values of data points in all figures

**Additional file 11.** A compressed file that included our original uncropped gel/blot images

## Data Availability

The datasets generated during the current study has been deposited at Gene Expression Omnibus (GEO) repository under the reference ID GSE171871 [76] and GSE208062 [77].

## Abbreviations

mESC: Mouse embryonic stem cell
PSC: Pluripotent stem cell
EpiLC: Epiblast like cell
DE: Definitive endoderm
XEN: Extraembryonic endoderm
HIF-1: Hypoxia-inducible factor 1
T: T-brachyury
Hh: Hedgehog
CK1: Casein kinase 1
CHIR: CHIR99021
Dox: Doxycycline
EB: Embryoid body
RNA-seq: RNA sequencing
PCA: Principal component analysis
DEG: Differentially expressed gene
GO: Gene ontology
GSEA: Gene Set Enrichment Analysis
RT-qPCR: Real-time quantitative RT-PCR
shHif-1α: Hif-1α knockdown
scramble: Scramble control
Hif-1α-iOE: Doxycycline-inducible Hif-1α-overexpression
HvsN_WT: Wildtype AB2.2 cells on differentiation day 4 under hypoxia versus normoxia
HvsN_shHif-1α: shHif-1α AB2.2 cells on differentiation day 4 under hypoxia versus normoxia
Hif-1α-OE_vs_Con: Hif-1α-iOE AB2.2 cells treated with 10 ng/mL Dox versus non-Dox control on differentiation day 4
