# Supplementary figures and images for: Hypoxia is fine-tuned by Hif-1α and regulates mesendoderm differentiation through the Wnt/β-Catenin pathway
Source: BMC Biol. 2022 Oct 5;20:219. doi: 10.1186/s12915-022-01423-y (PMC9536055; doi:10.1186/s12915-022-01423-y)

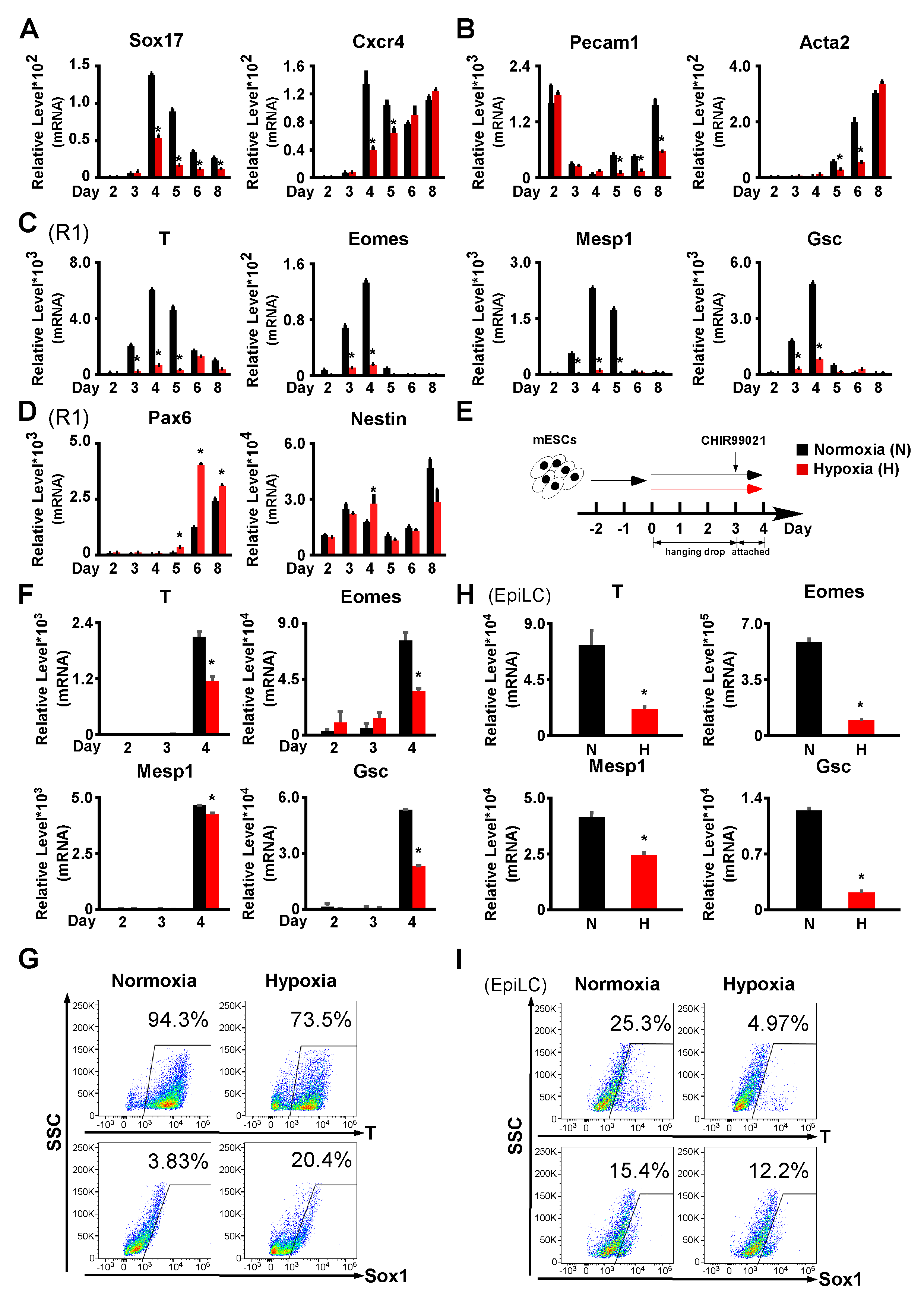

Supplement: Supplementary file 1 — Additional file 1: Fig. S1. Further validation of the effects of hypoxia on mESC differentiation. (A) Hypoxia significantly repressed the mRNA expression of endoderm markers (Sox17 and Cxcr4) in differentiating AB2.2 mESCs. (B) Hypoxia significantly repressed the mRNA expression of an endothelial marker (Pecam1) and a smooth muscle marker (Acta2) in differentiating AB2.2 mESCs. (C) Hypoxia significantly repressed the expression of mesendoderm markers (T, Eomes, Mesp1, and Gsc) in differentiating R1 mESCs. (D) Hypoxia significantly upregulated the mRNA expression of ectoderm markers (Pax6 and Nestin) in differentiating R1 mESCs. (E) Schematic diagram of AB2.2 mESC differentiation under normoxia or hypoxia with CHIR treatment on differentiation day 3-4. (F) In the differentiation shown in (E), the expression of mesendoderm markers (T, Eomes, Mesp1, and Gsc) was also repressed by hypoxia. (G) In the differentiation shown in (E), the ratios of T+ and Sox1+ cells were downregulated and upregulated, respectively, by hypoxia. (H) In the mesendoderm differentiation from epiblast like cells (EpiLCs), the expression of mesendoderm markers (T, Eomes, Mesp1, and Gsc) was also repressed by hypoxia. (I) In the mesendoderm differentiation from EpiLCs, the T+ cell ratio was downregulated, while the Sox1+ cell ratio was minorly altered. R1, the assays performed in R1 mESCs; EpiLC, the assays performed in epiblast like cells; N, normoxia; H, hypoxia; *, significant (P<0.05). [file 12915_2022_1423_MOESM1_ESM.tif]

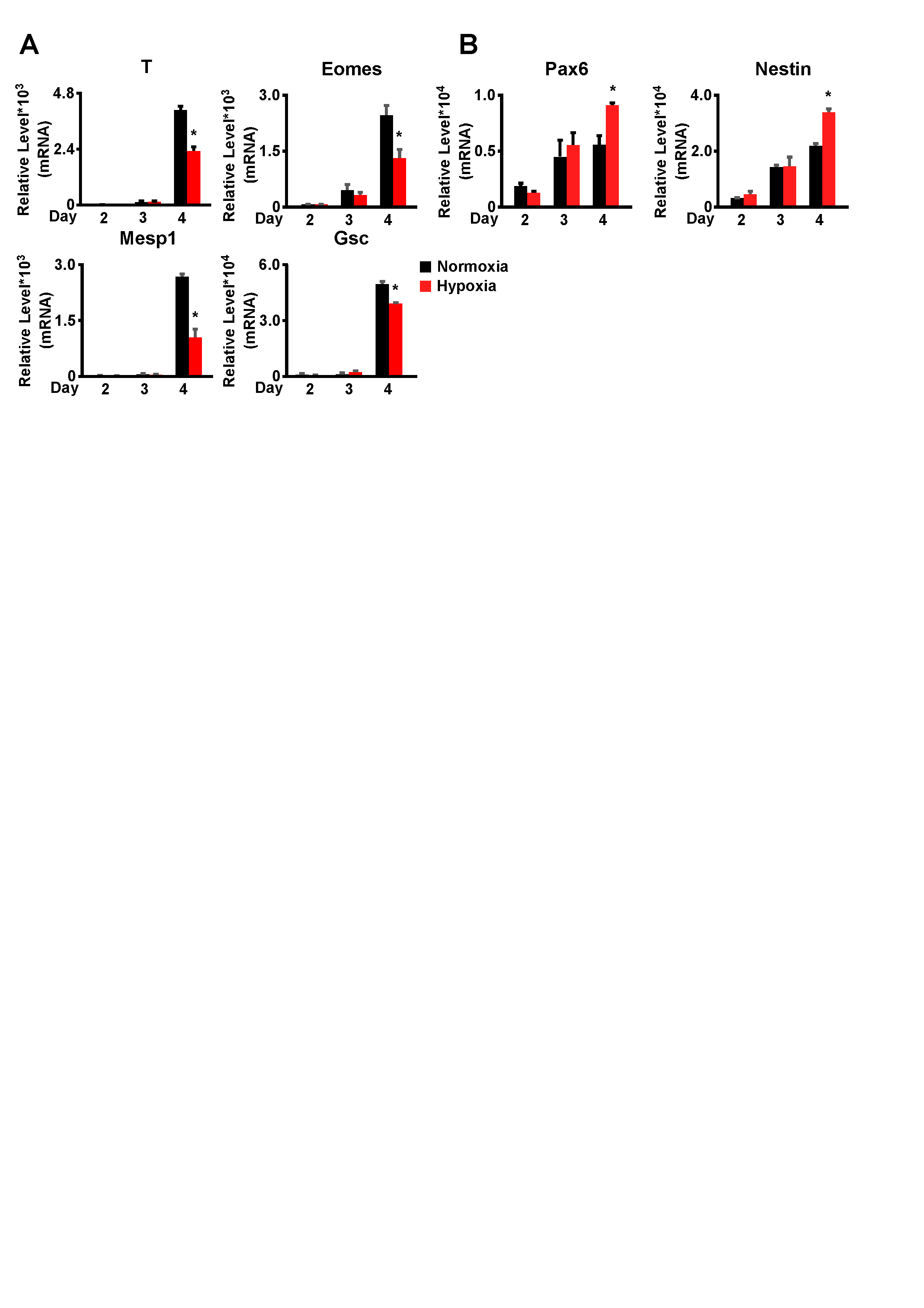

Supplement: Supplementary file 2 — Additional file 2: Fig. S2. The effects of hypoxia on AB2.2 mESC differentiation performed with a high-serum differentiation medium. The differentiation in the high-serum differentiation medium was non-lineage-prone. In the differentiation performed with the high-serum medium, the expressions of (A) mesendoderm markers (T, Eomes, Mesp1, and Gsc) and (B) ectoderm markers (Pax6 and Nestin) were downregulated and upregulated by hypoxia, respectively. *, significant (P<0.05). [file 12915_2022_1423_MOESM2_ESM.tif]

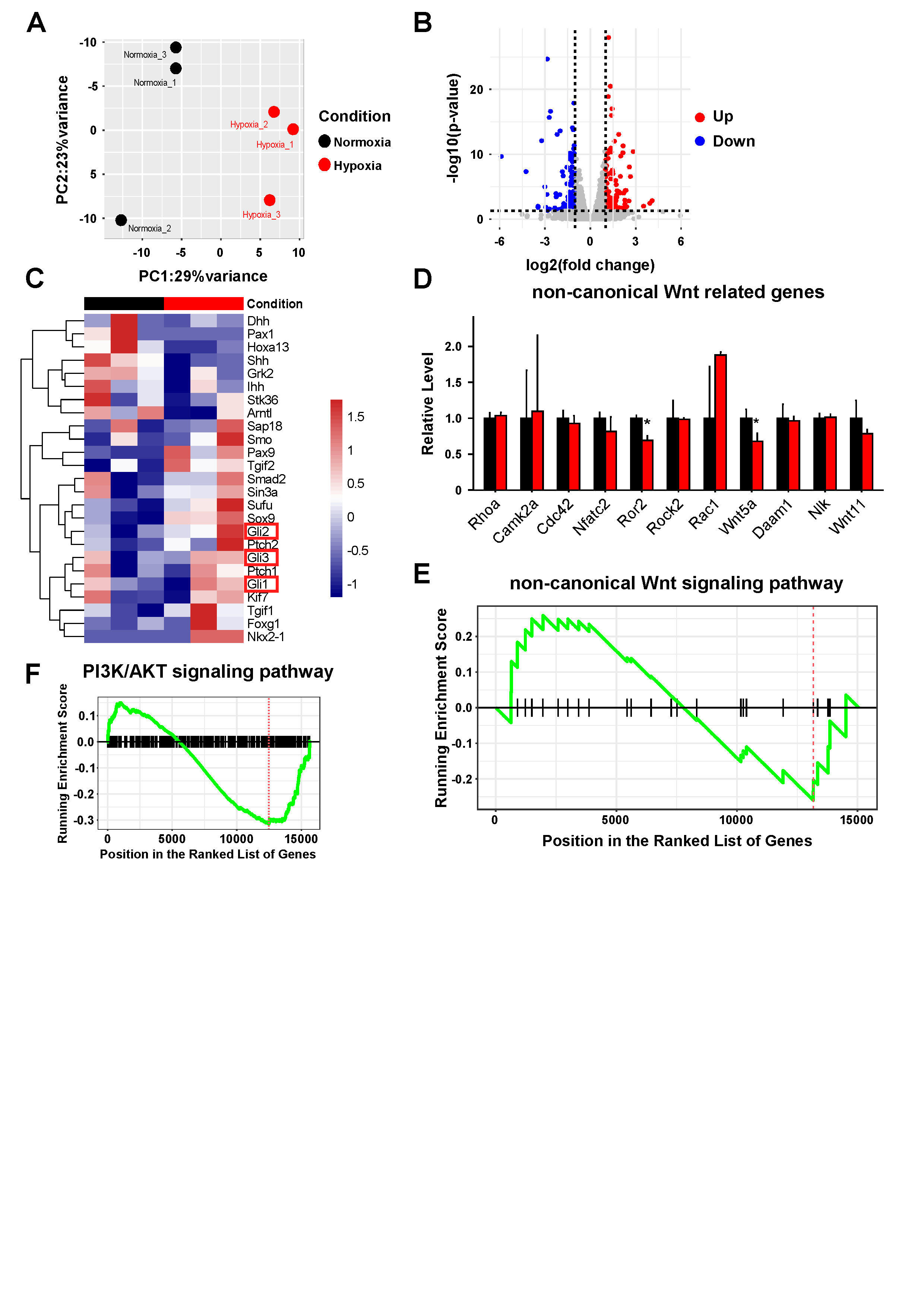

Supplement: Supplementary file 3 — Additional file 3: Fig. S3. The supplementary analyses performed on the RNA-seq data of AB2.2 mESCs on differentiation day 4 under normoxia and hypoxia. (A) PCA of RNA-seq data from normoxia and hypoxia groups. The PCA included three biological repeats for each group. (B) The volcano plot showed the differentially expressed genes (DEGs). |Log2(Fold Change)|>1 and adjusted P-value<0.05 were used as the cutoff criteria. (C) The related levels of Hh signaling-related genes in AB2.2 mESCs on differentiation day 4 under hypoxia versus normoxia. (D) The related levels of noncanonical Wnt pathway-related genes in AB2.2 mESCs on differentiation day 4 under hypoxia versus normoxia. (E) GSEA showed that the noncanonical pathway activation was not uniformly regulated by hypoxia. (F) GSEA showed that the PI3K/Akt signaling pathway was significantly repressed by hypoxia. *, significant (P<0.05). [file 12915_2022_1423_MOESM3_ESM.tif]

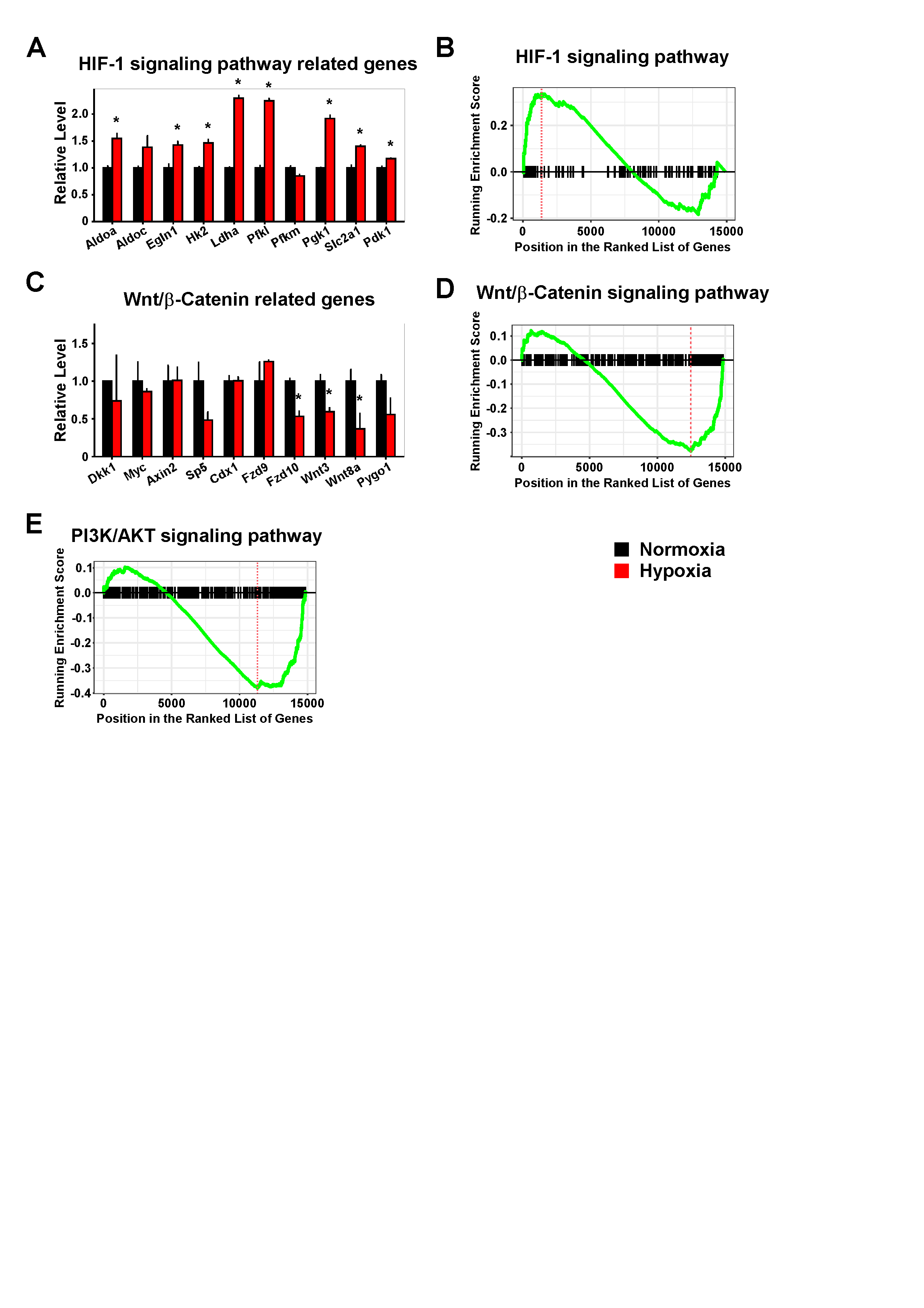

Supplement: Supplementary file 4 — Additional file 4: Fig. S4. The analyses performed on the RNA-seq data of AB2.2 mESCs on differentiation day 2 under normoxia and hypoxia. (A) The related levels of HIF-1 signaling-related genes in AB2.2 mESCs on differentiation day 2 under hypoxia versus normoxia. (B) GSEA showed that the HIF-1 signaling pathway activation was significantly enhanced by hypoxia. (C) The related levels of Wnt/β-Catenin pathway-related genes in AB2.2 mESCs on differentiation day 2 under hypoxia versus normoxia. (D) GSEA showed that the Wnt/β-Catenin pathway was significantly repressed by hypoxia (E) GSEA showed that the PI3K/Akt signaling pathway was significantly repressed by hypoxia. *, significant (P<0.05). [file 12915_2022_1423_MOESM4_ESM.tif]

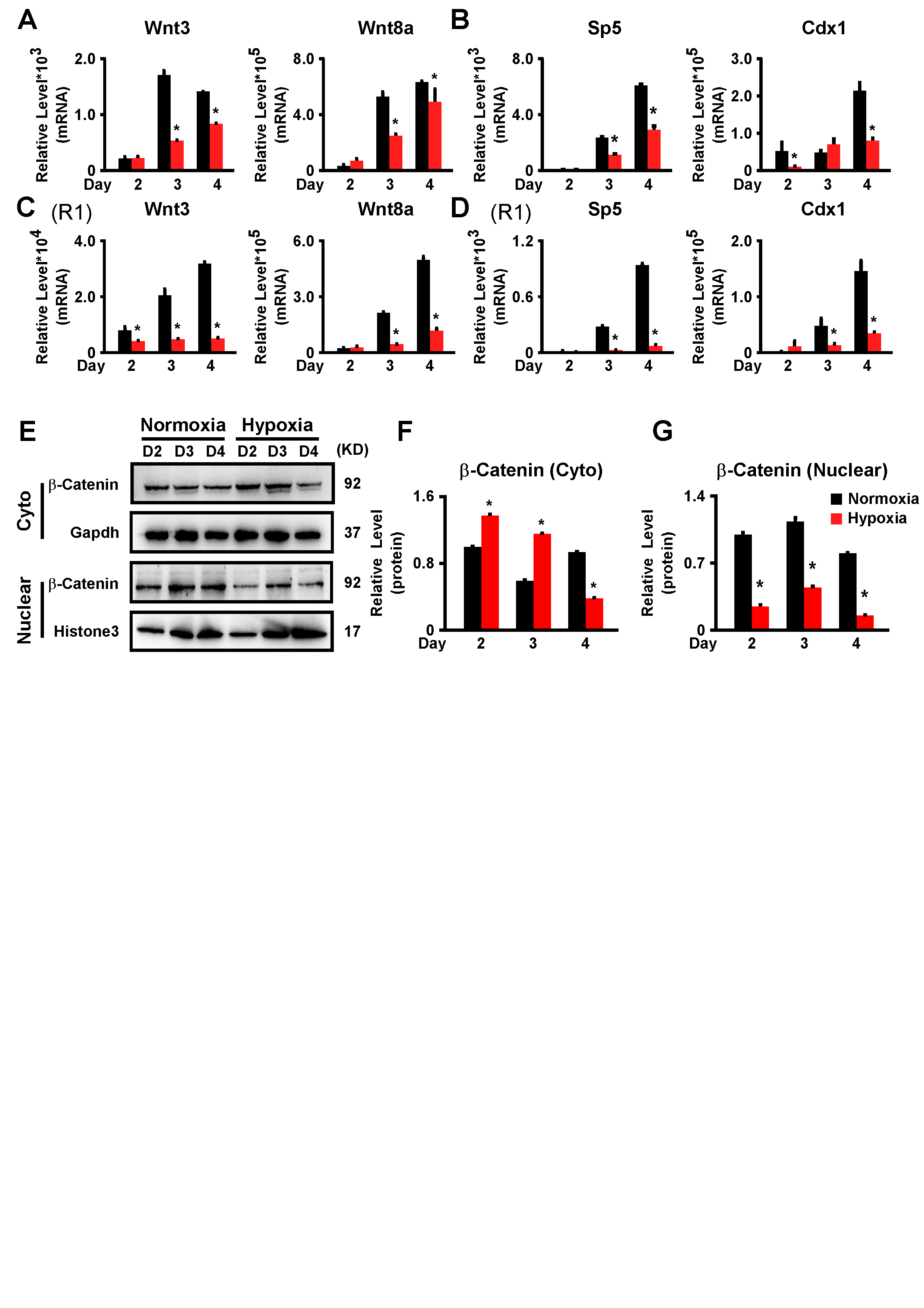

Supplement: Supplementary file 5 — Additional file 5: Fig. S5. The Wnt/β-Catenin pathway was affected by hypoxia. (A) The expression of canonical Wnts (Wnt3 and Wnt8a) and (B) Wnt/β-Catenin pathway downstream targets (Sp5 and Cdx1) was downregulated by hypoxia in AB2.2 mESCs undergoing differentiation. (C) The expression of canonical Wnts (Wnt3 and Wnt8a) and (D) Wnt/β-Catenin pathway downstream targets (Sp5 and Cdx1) was downregulated by hypoxia in R1 mESCs undergoing differentiation. (E-G) Expression of nuclear β-Catenin was significantly repressed, while that of cytoplasmic β-Catenin was upregulated until day 3 and downregulated on day 4 with hypoxia treatment. *, significant (P<0.05). [file 12915_2022_1423_MOESM5_ESM.tif]

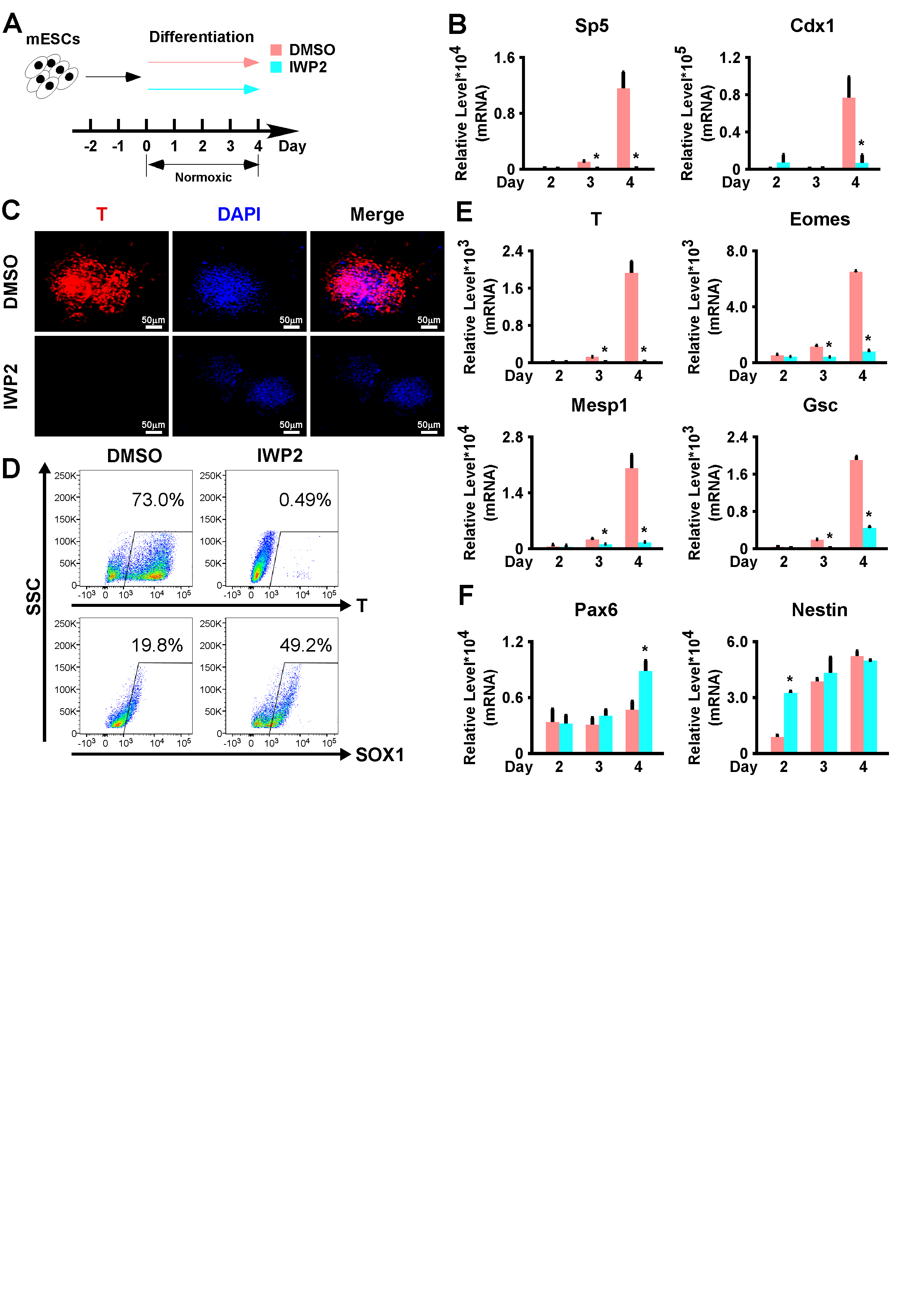

Supplement: Supplementary file 6 — Additional file 6: Fig. S6. The Wnt/β-Catenin pathway and AB2.2 mESC differentiation were affected by IWP2. (A) Schematic diagram of mESC differentiation treated with or without IWP2 under normoxia. (B) The expression of Wnt/β-Catenin downstream targets (Sp5 and Cdx1) was severely inhibited by IWP2 in AB2.2 mESCs undergoing differentiation. (C) IWP2 repressed T protein expression (red) on differentiation day 4. Nuclei were stained with DPAI (blue). (D) The ratios of T+ and Sox1+ cells were downregulated and upregulated by IWP2, respectively. (E) IWP2 treatment significantly repressed the mRNA expression of mesendoderm markers (T, Eomes, Mesp1, and Gsc) in AB2.2 mESCs undergoing differentiation. (F) Inhibiting the Wnt/β-Catenin pathway by IWP2 upregulated the expression of ectoderm markers (Pax6 and Nestin). *, significant (P<0.05). [file 12915_2022_1423_MOESM6_ESM.tif]

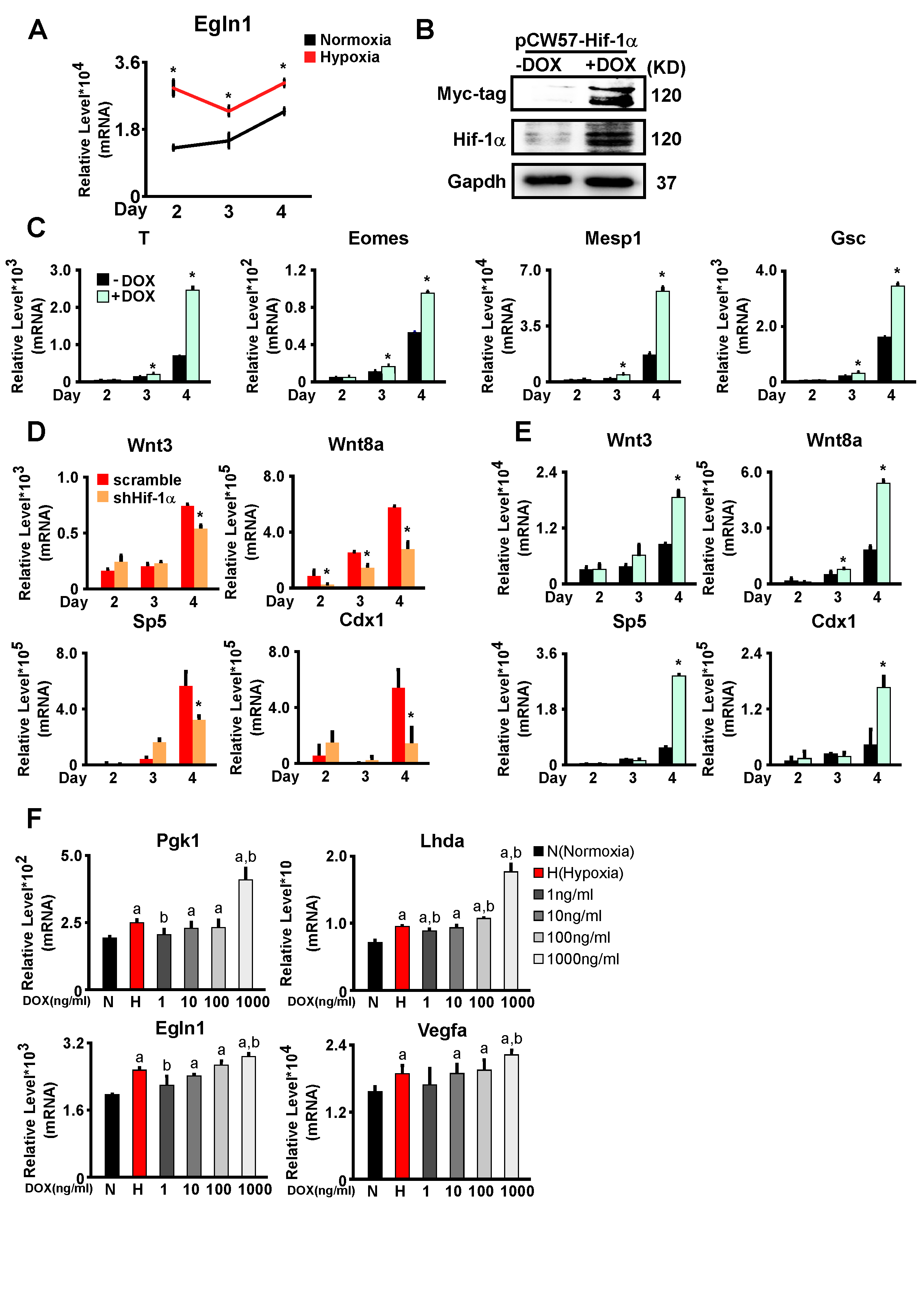

Supplement: Supplementary file 7 — Additional file 7: Fig. S7. The supplementary data of the effects of Hif-1α on the mesendoderm differentiation and Wnt/β-Catenin pathway. (A) The expression patterns of Egln1 in the differentiating AB2.2 mESCs under normoxia and hypoxia. (B) Doxycycline (Dox)-inducible overexpression of oxygen-resistant Hif-1α was verified by western blotting analysis. A Myc tag was added to the C-terminus of Hif-1α. (C) The mRNA expression of mesendoderm markers (T, Eomes, Mesp1, and Gsc) in AB2.2 mESCs with or without Dox triggered Hif-1α overexpression under normoxia. (D) The mRNA expression of canonical Wnts (Wnt3 and Wnt8a) and Wnt/β-Catenin pathway downstream targets (Sp5 and Cdx1) in scramble control and shHif-1α AB2.2 mESCs under hypoxia. (E) The mRNA expression of canonical Wnts (Wnt3 and Wnt8a) and Wnt/β-Catenin pathway downstream targets (Sp5 and Cdx1) in AB2.2 mESCs with or without Dox triggered Hif-1α overexpression under normoxia. (F) The expression changes of the HIF-1 signaling targets (Pgk1, Ldha, Egln1, and Vegfa) in Hif-1α-iOE AB2.2 mESCs treated with 1, 10, 100, and 1000 ng/mL Dox, respectively. Normoxic and hypoxic cultures without dox treatment were used as controls. N, normoxia; H, hypoxia; a, significant (P<0.05) compared to the normoxia group; b, significant (P<0.05) compared to the hypoxia group. pCW57-Hif-1α, AB2.2/DOX-inducible overexpression of oxygen-resistant Hif-1α; scramble, AB2.2/scramble cells; shHif-1α, AB2.2/shHif-1α cells; DOX, doxycycline; *, significant (P<0.05). [file 12915_2022_1423_MOESM7_ESM.tif]

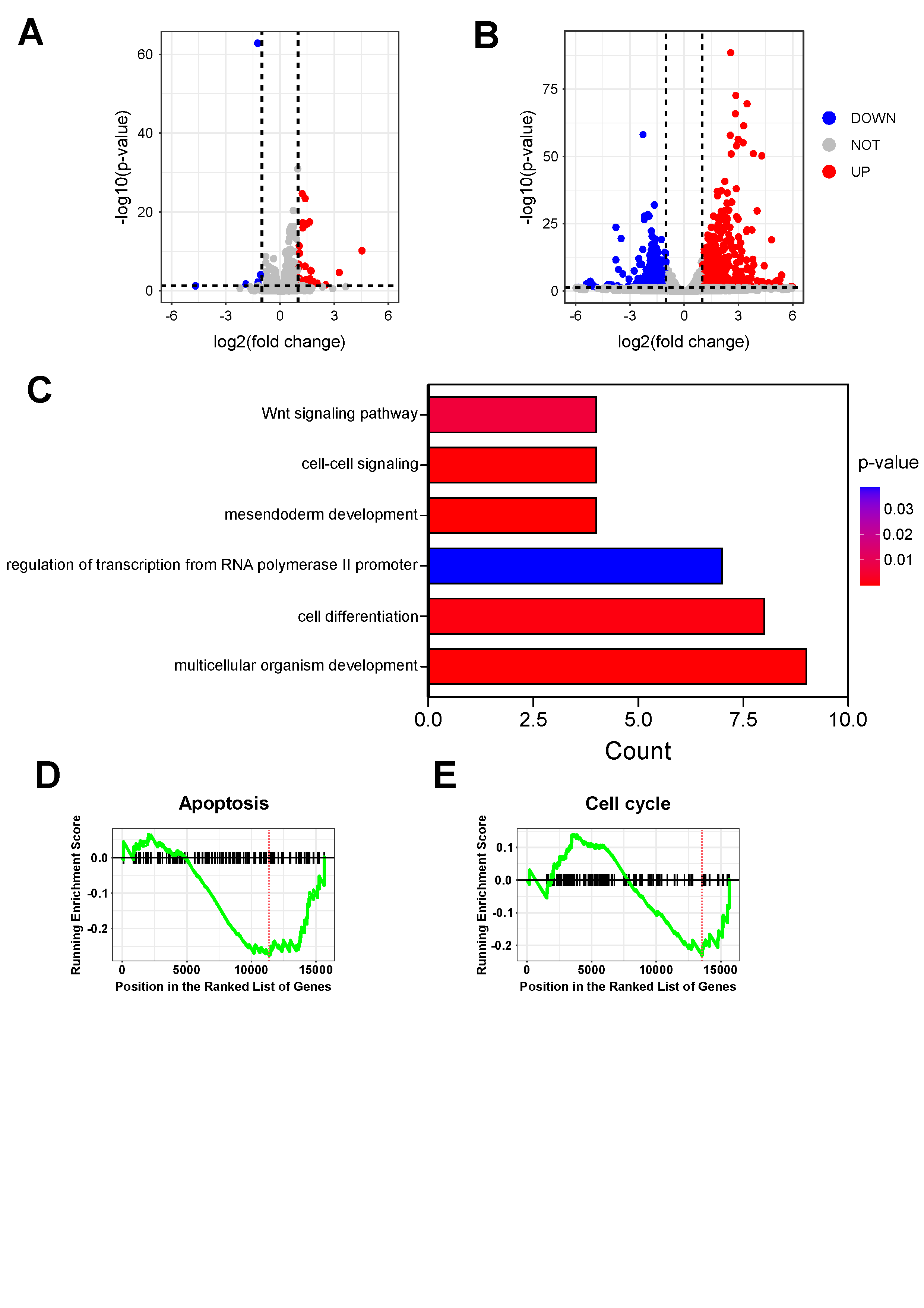

Supplement: Supplementary file 8 — Additional file 8: Fig. S8. The supplementary analyses performed on the RNA-seq data of normoxia, hypoxia, control, and Hif-1α overexpression groups. The volcano plot showed the differentially expressed genes (DEGs) in (A) Hif-1α-OE_vs_Con and (B) HvsN_shHif-1α. |Log2(Fold Change)|>1 and adjusted P-value<0.05 were used as the cutoff criteria. (C) The GO terms of the DEGs upregulated by Hif-1α overexpression. GSEA of (D) apoptosis and (E) cell of AB2.2 mESCs on differentiation day 4 under hypoxia versus normoxia. [file 12915_2022_1423_MOESM8_ESM.tif]

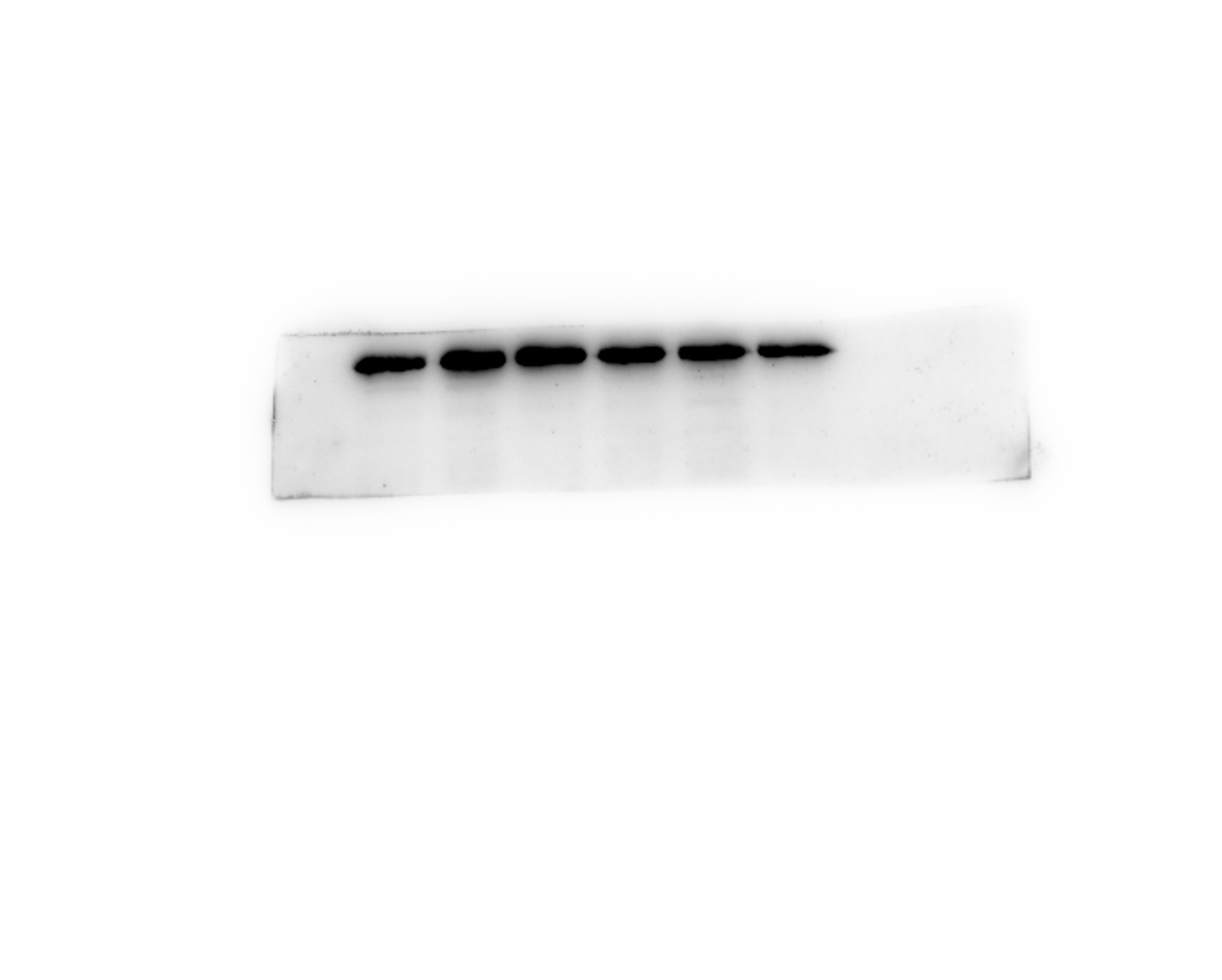

Supplement: Supplementary file 11 — Additional file 11. A compressed file that included our original uncropped gel/blot images [file 12915_2022_1423_MOESM11_ESM.zip › Additonal file 11/Figure 3B/Gapdh.tif]

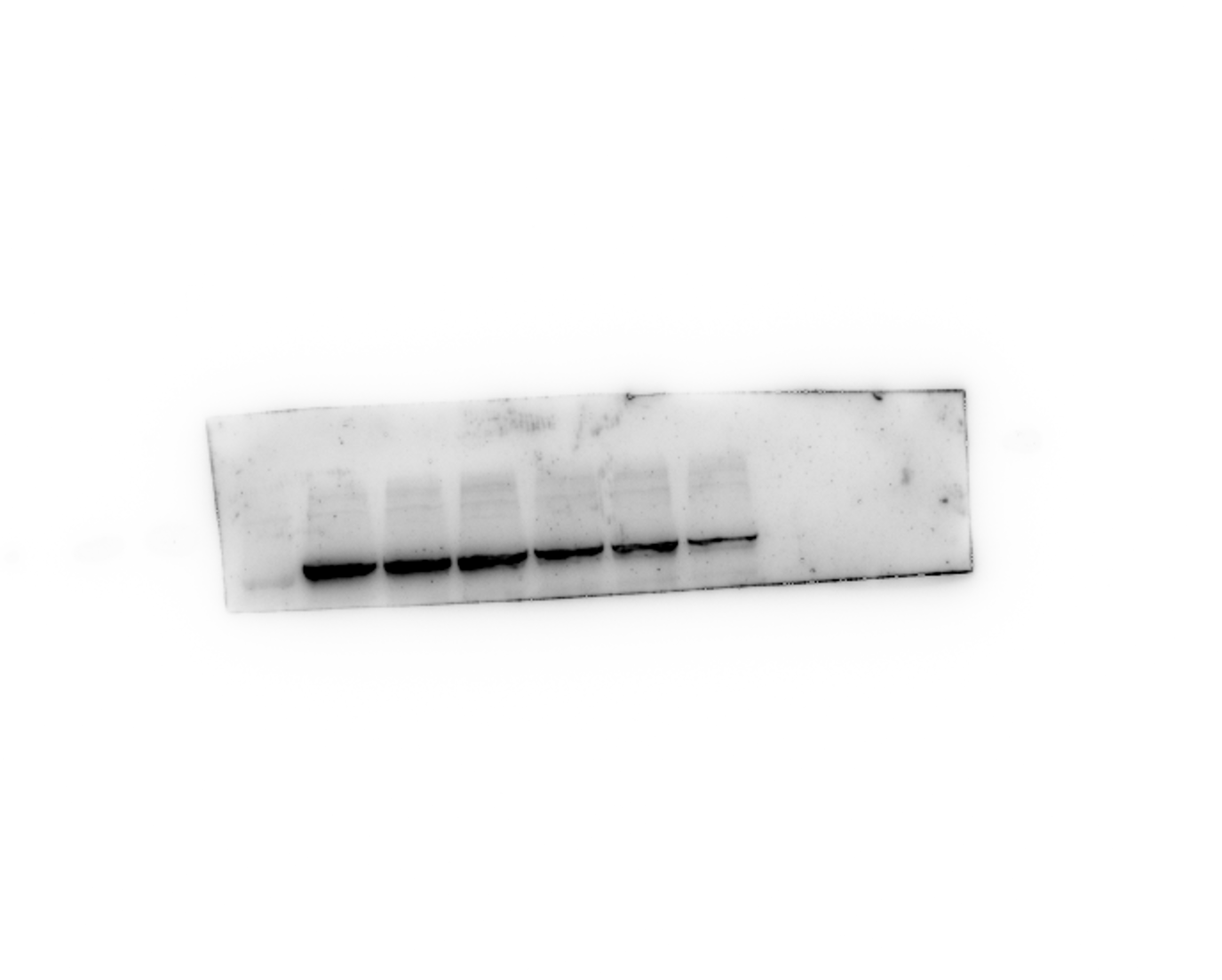

Supplement: Supplementary file 11 — Additional file 11. A compressed file that included our original uncropped gel/blot images [file 12915_2022_1423_MOESM11_ESM.zip › Additonal file 11/Figure 3B/b-Catenin.tif]

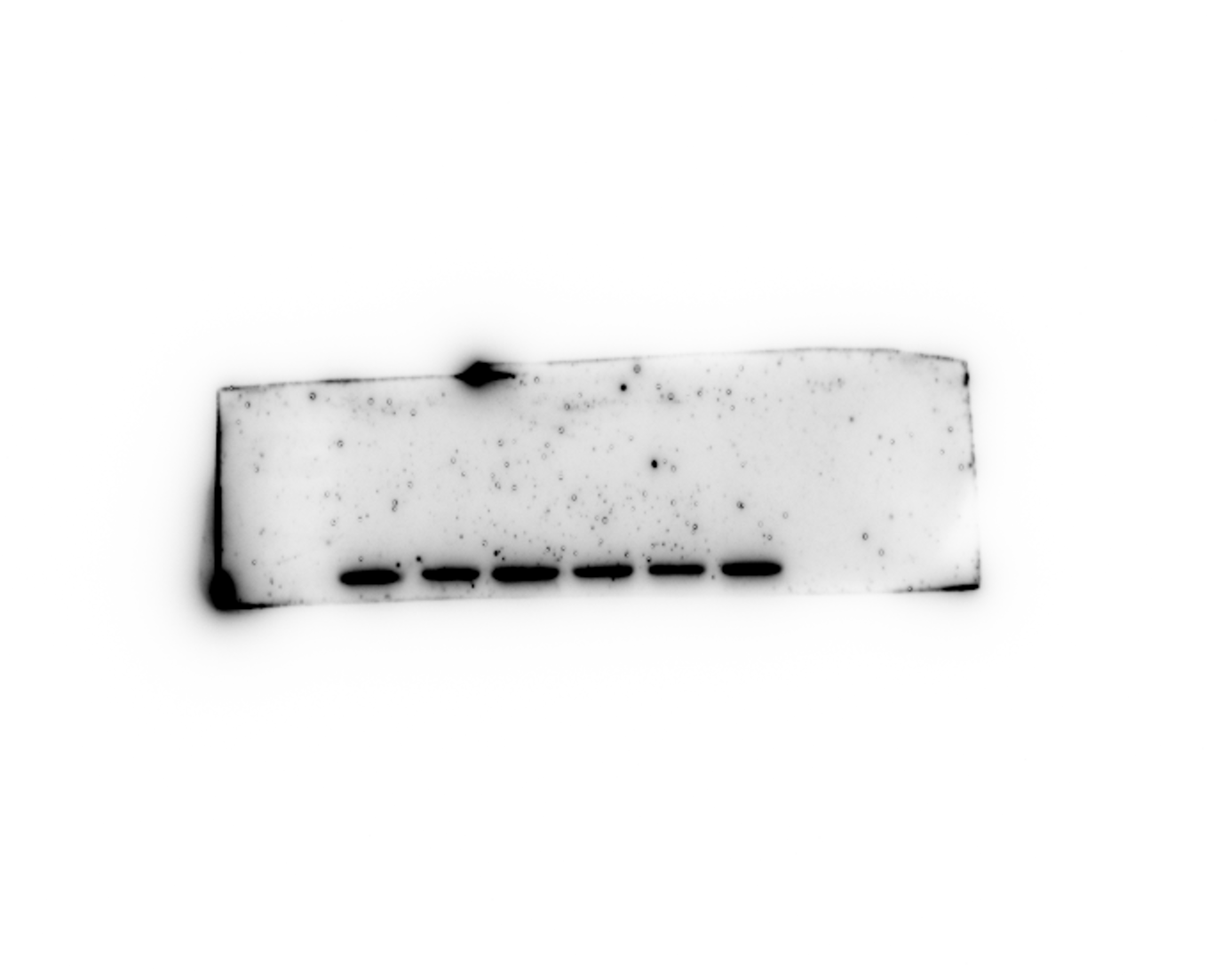

Supplement: Supplementary file 11 — Additional file 11. A compressed file that included our original uncropped gel/blot images [file 12915_2022_1423_MOESM11_ESM.zip › Additonal file 11/Figure 3F/GSK3b.tif]

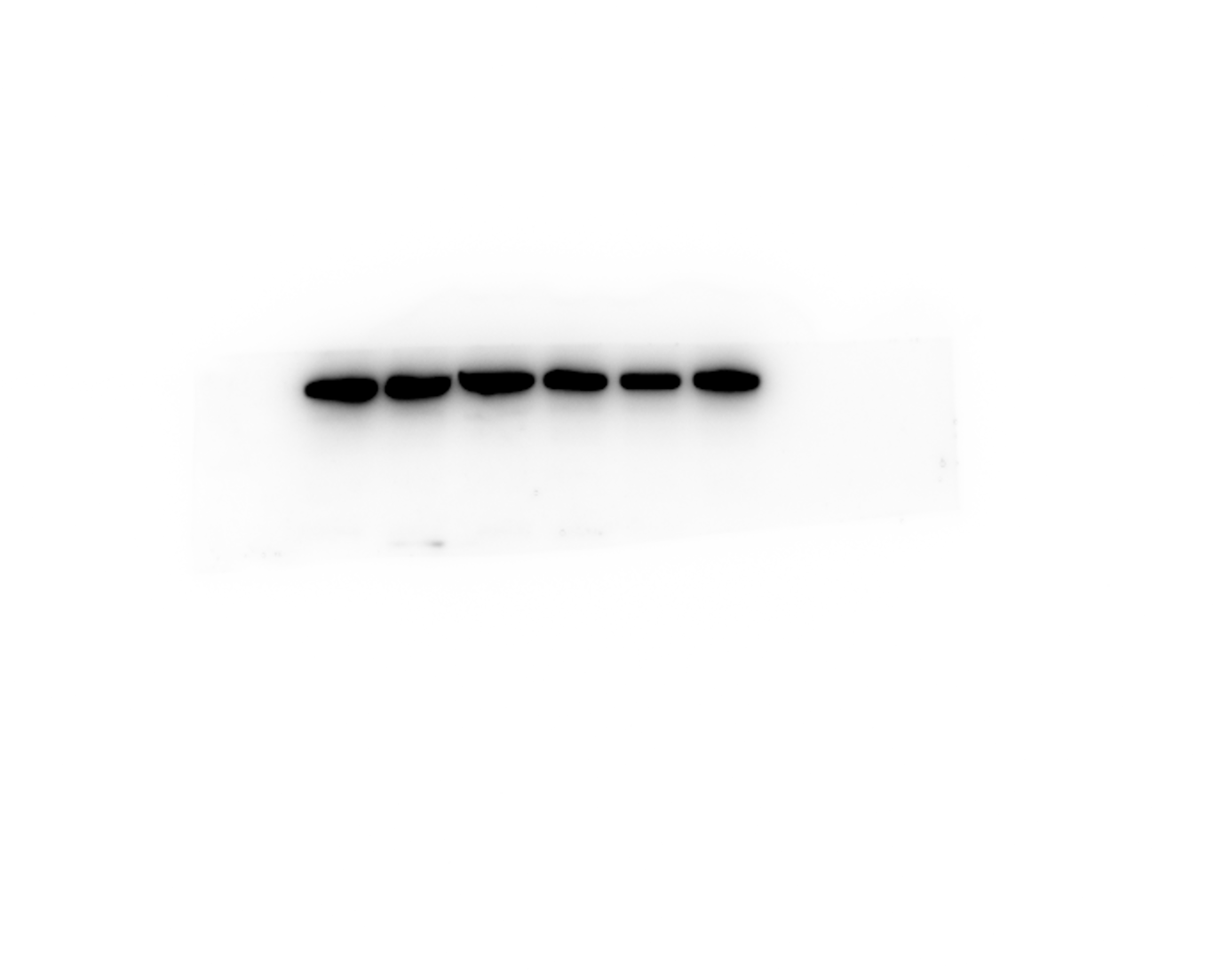

Supplement: Supplementary file 11 — Additional file 11. A compressed file that included our original uncropped gel/blot images [file 12915_2022_1423_MOESM11_ESM.zip › Additonal file 11/Figure 3F/Gapdh.tif]

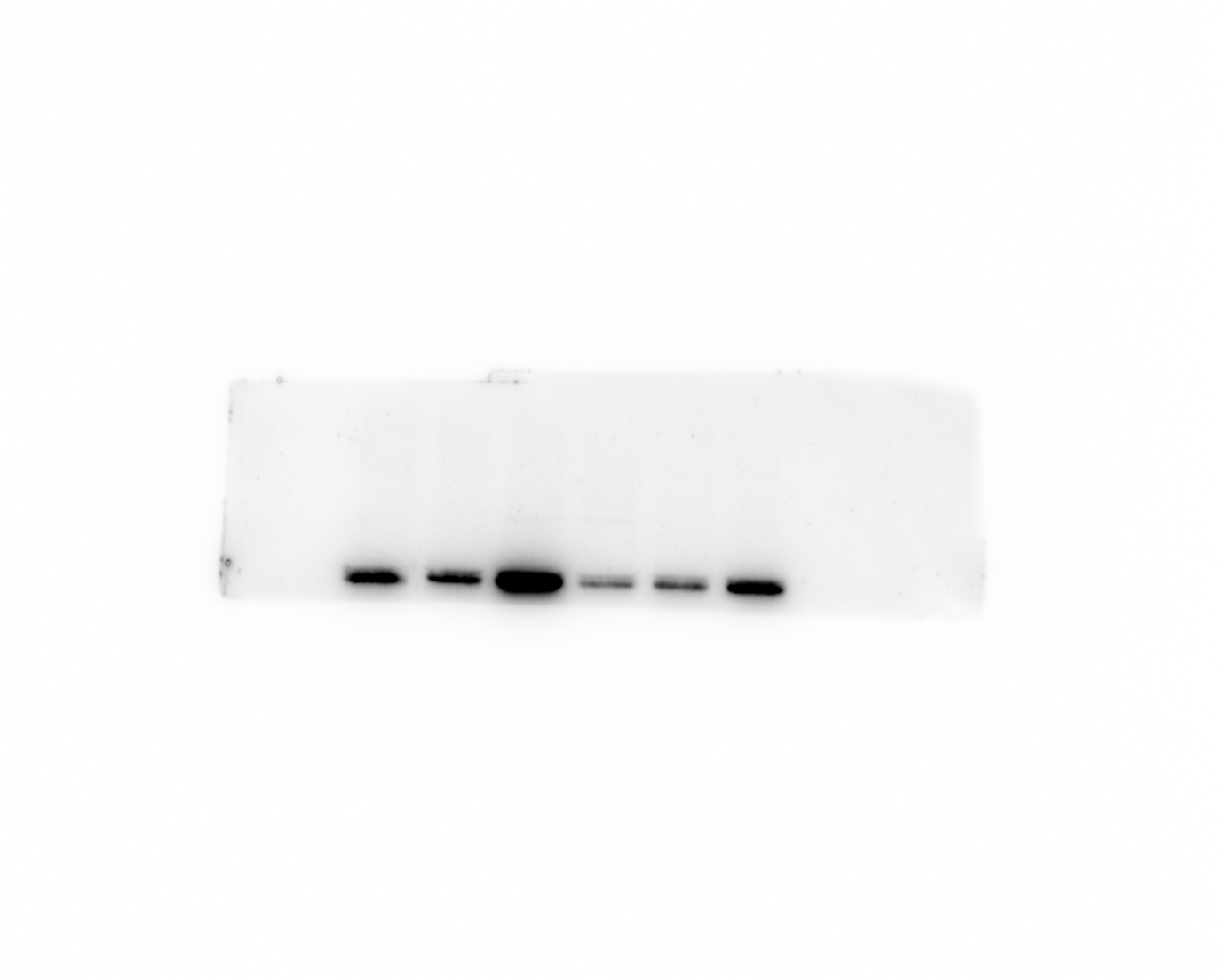

Supplement: Supplementary file 11 — Additional file 11. A compressed file that included our original uncropped gel/blot images [file 12915_2022_1423_MOESM11_ESM.zip › Additonal file 11/Figure 3F/p-GSK3b.tif]

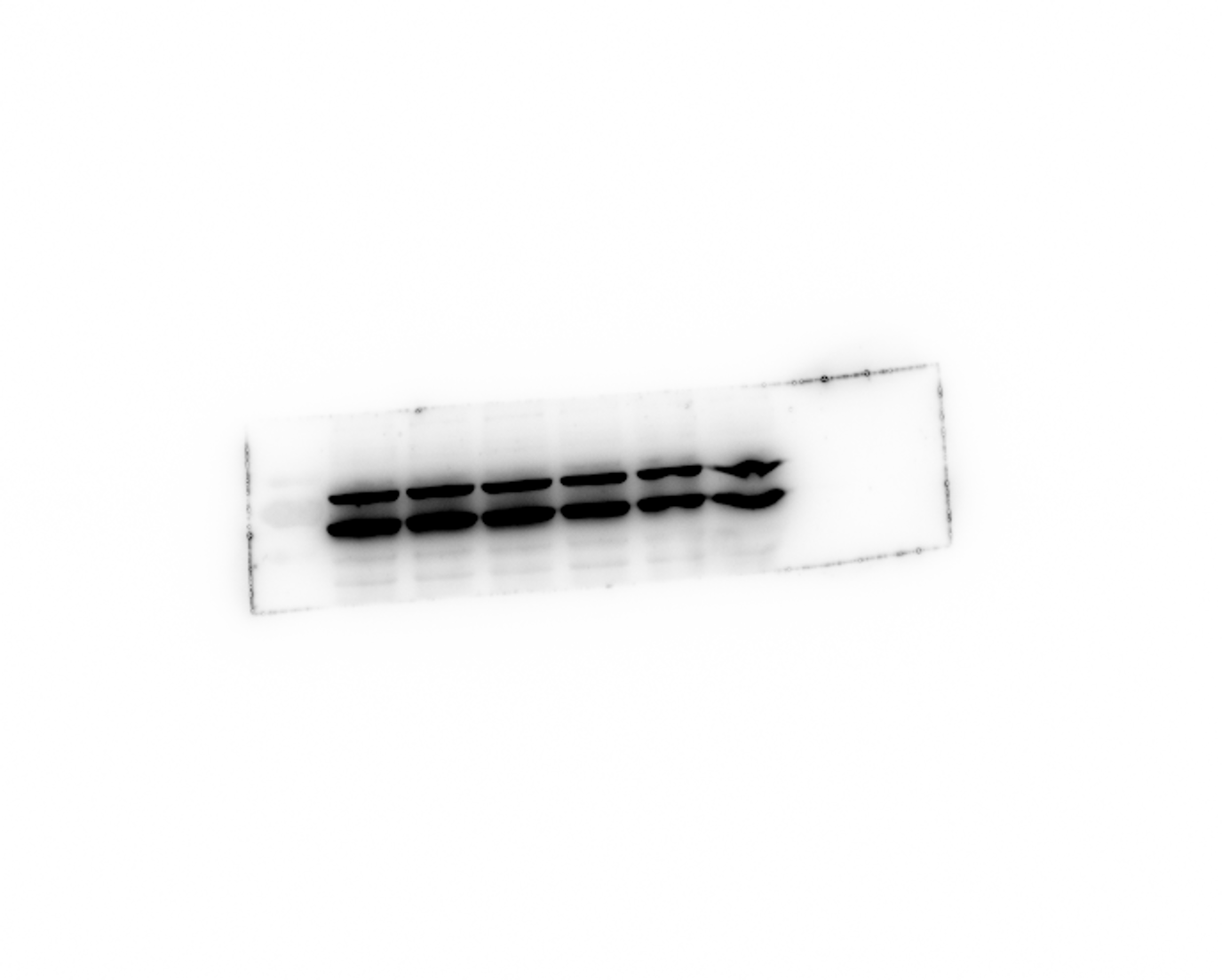

Supplement: Supplementary file 11 — Additional file 11. A compressed file that included our original uncropped gel/blot images [file 12915_2022_1423_MOESM11_ESM.zip › Additonal file 11/Figure 3H/AKT.tif]

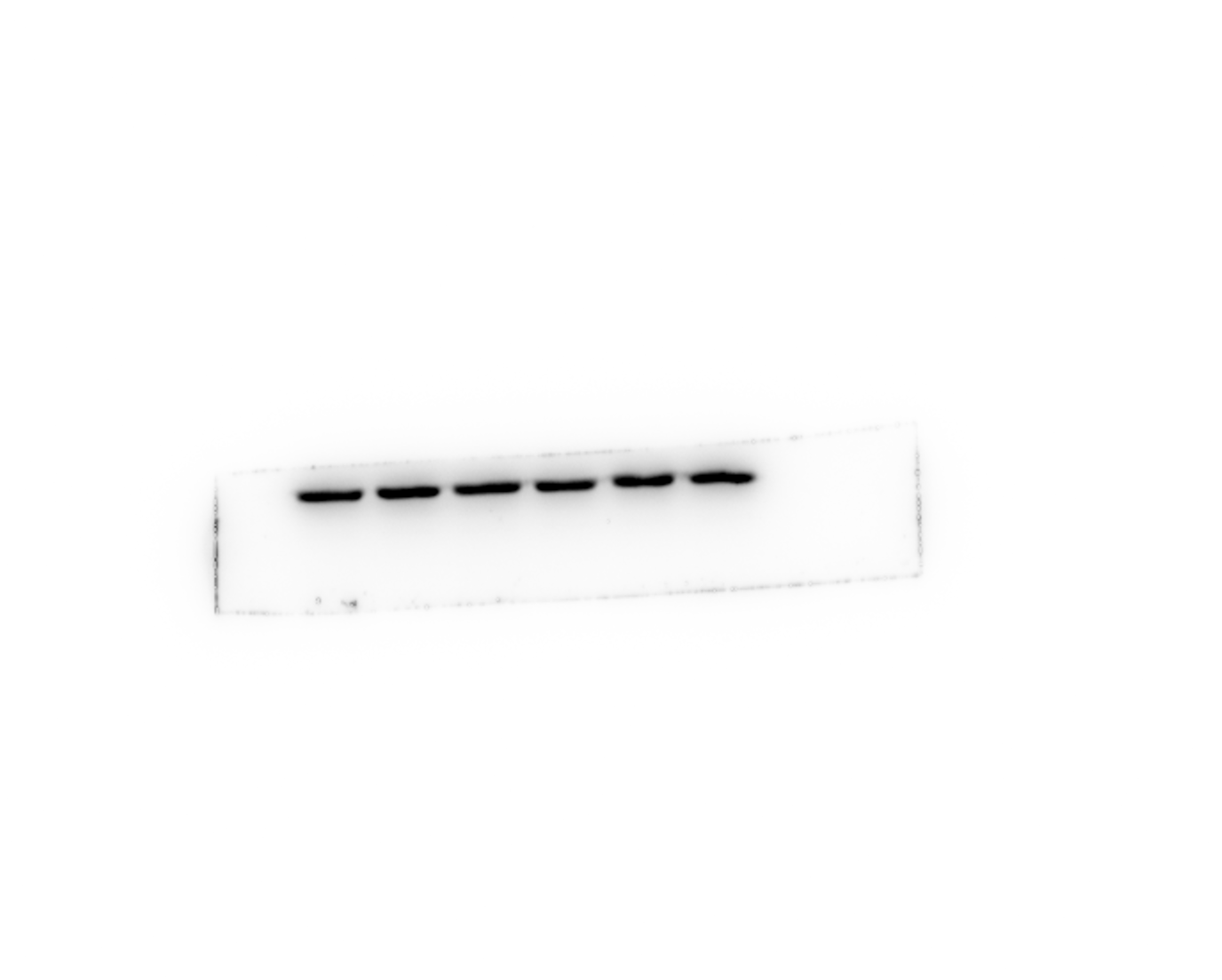

Supplement: Supplementary file 11 — Additional file 11. A compressed file that included our original uncropped gel/blot images [file 12915_2022_1423_MOESM11_ESM.zip › Additonal file 11/Figure 3H/Gapdh.tif]

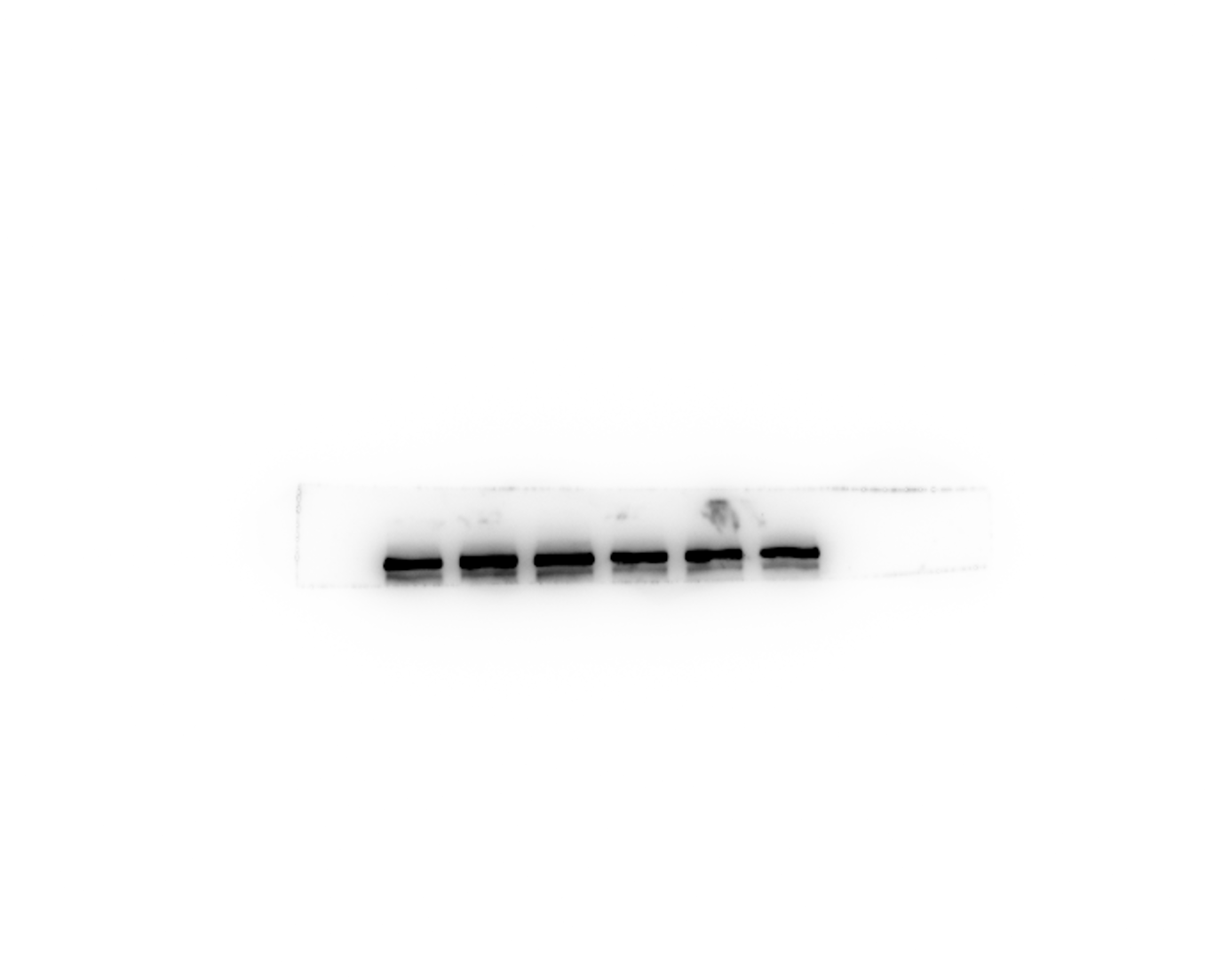

Supplement: Supplementary file 11 — Additional file 11. A compressed file that included our original uncropped gel/blot images [file 12915_2022_1423_MOESM11_ESM.zip › Additonal file 11/Figure 3H/mTOR.tif]

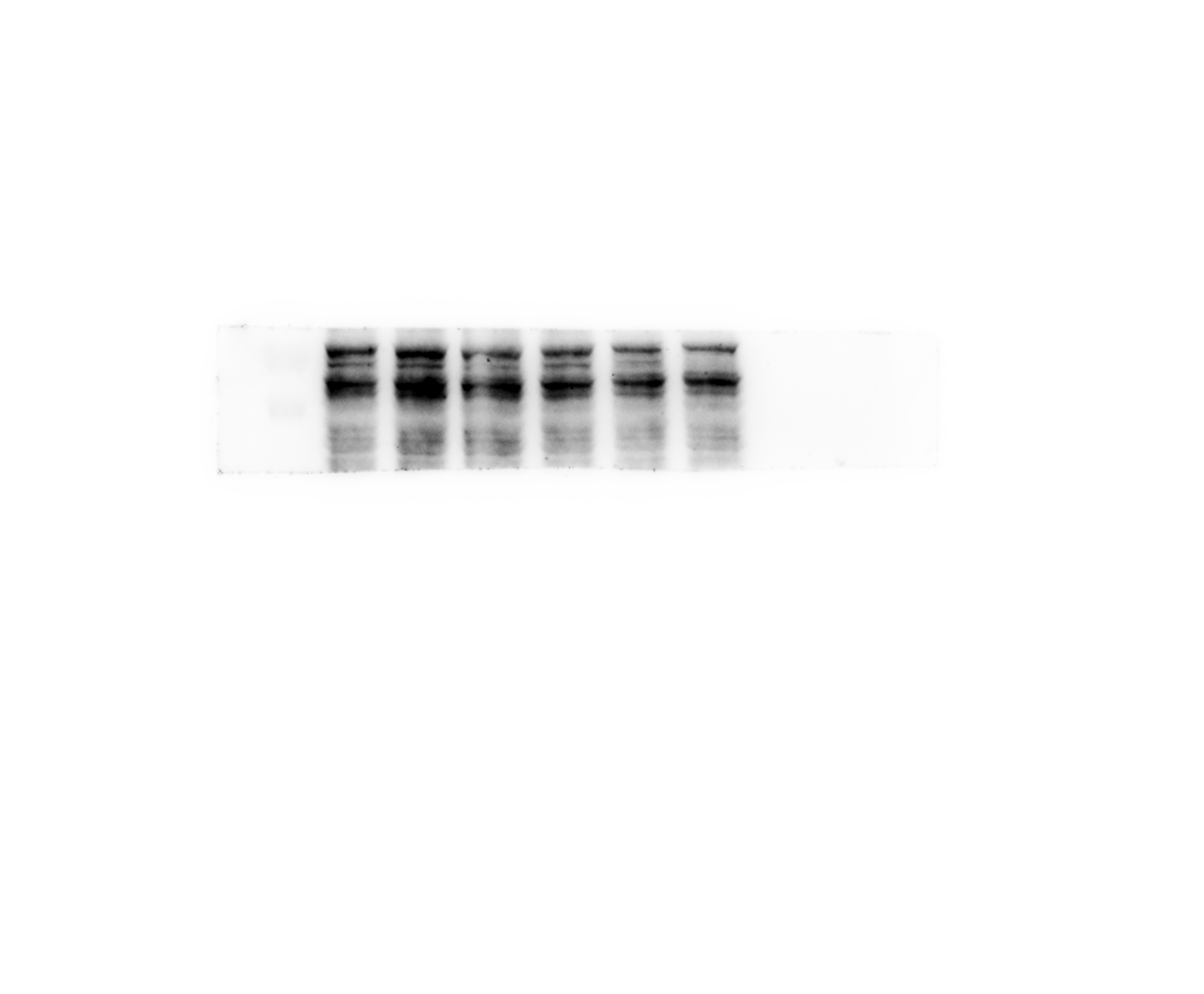

Supplement: Supplementary file 11 — Additional file 11. A compressed file that included our original uncropped gel/blot images [file 12915_2022_1423_MOESM11_ESM.zip › Additonal file 11/Figure 3H/p-AKT.tif]

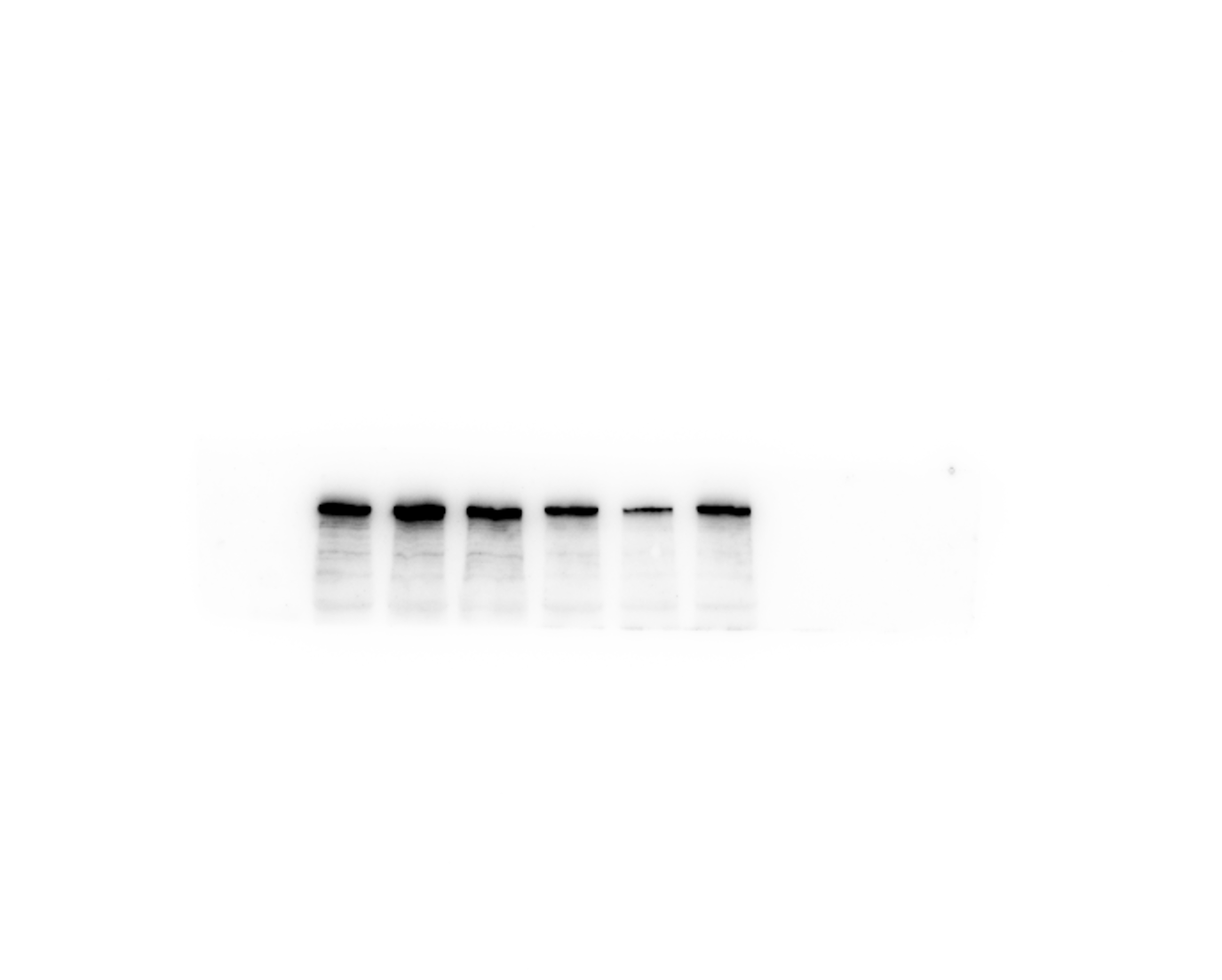

Supplement: Supplementary file 11 — Additional file 11. A compressed file that included our original uncropped gel/blot images [file 12915_2022_1423_MOESM11_ESM.zip › Additonal file 11/Figure 3H/p-mTOR.tif]

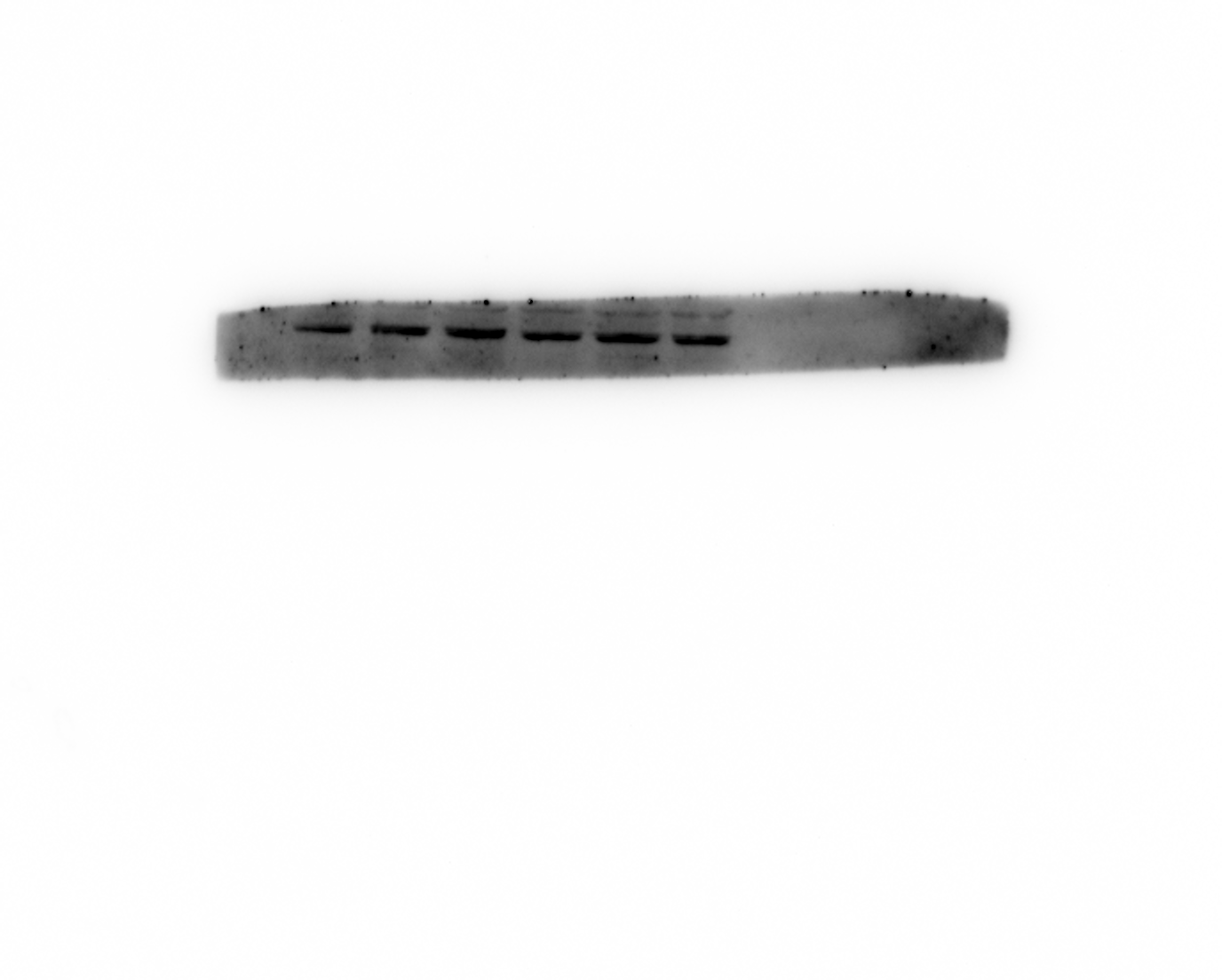

Supplement: Supplementary file 11 — Additional file 11. A compressed file that included our original uncropped gel/blot images [file 12915_2022_1423_MOESM11_ESM.zip › Additonal file 11/Figure 3L/AKT.tif]

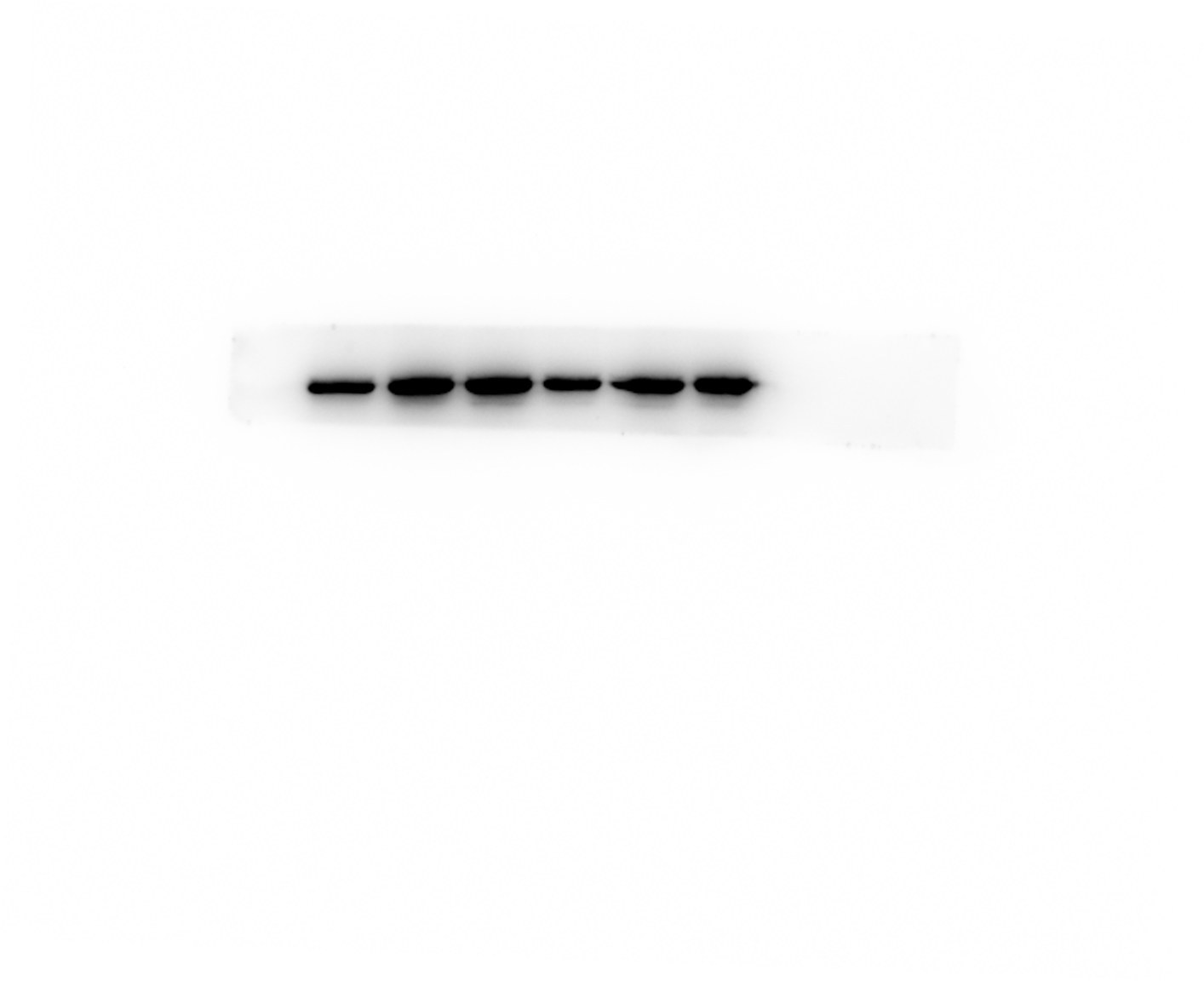

Supplement: Supplementary file 11 — Additional file 11. A compressed file that included our original uncropped gel/blot images [file 12915_2022_1423_MOESM11_ESM.zip › Additonal file 11/Figure 3L/GSK3b.tif]

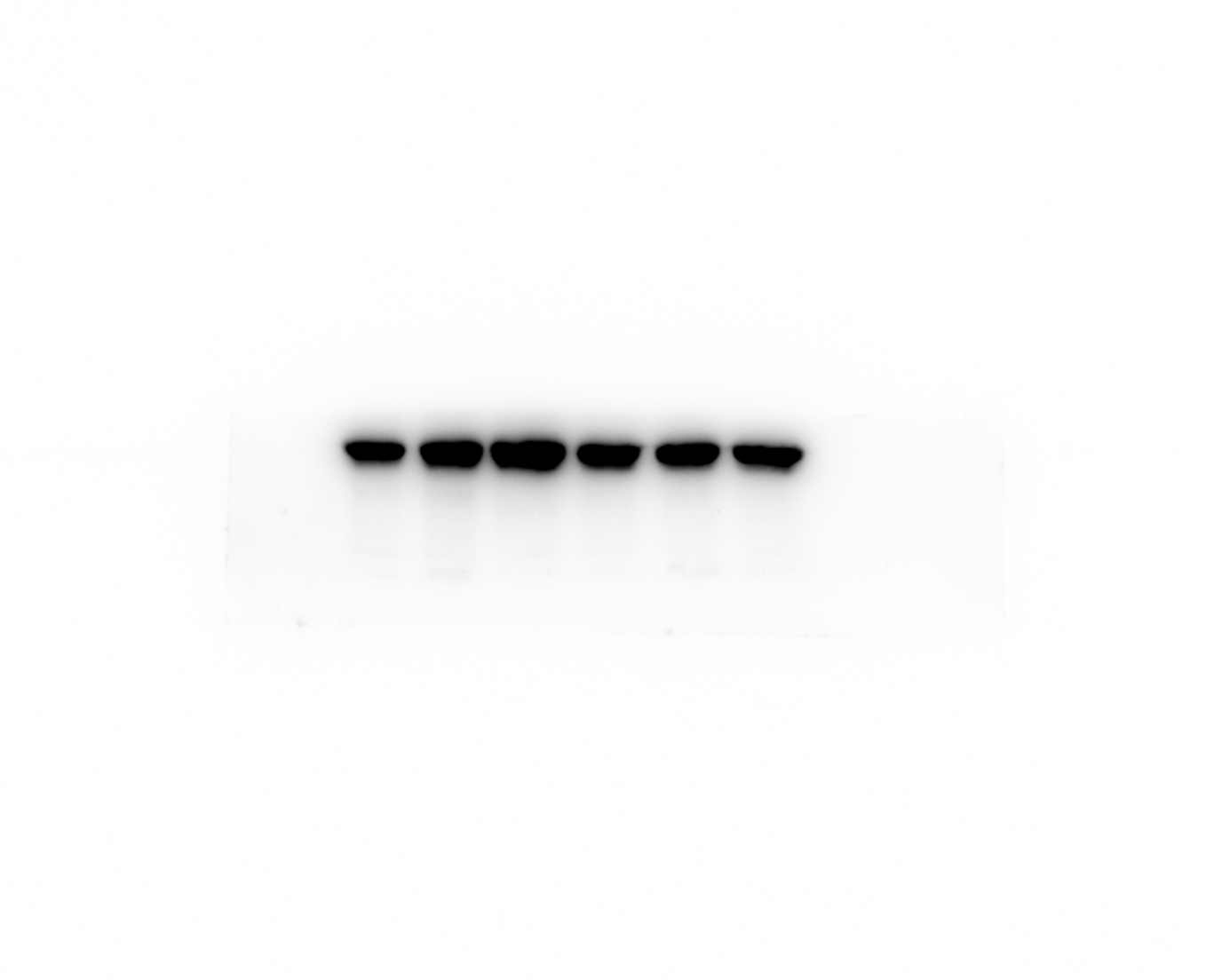

Supplement: Supplementary file 11 — Additional file 11. A compressed file that included our original uncropped gel/blot images [file 12915_2022_1423_MOESM11_ESM.zip › Additonal file 11/Figure 3L/Gapdh.tif]

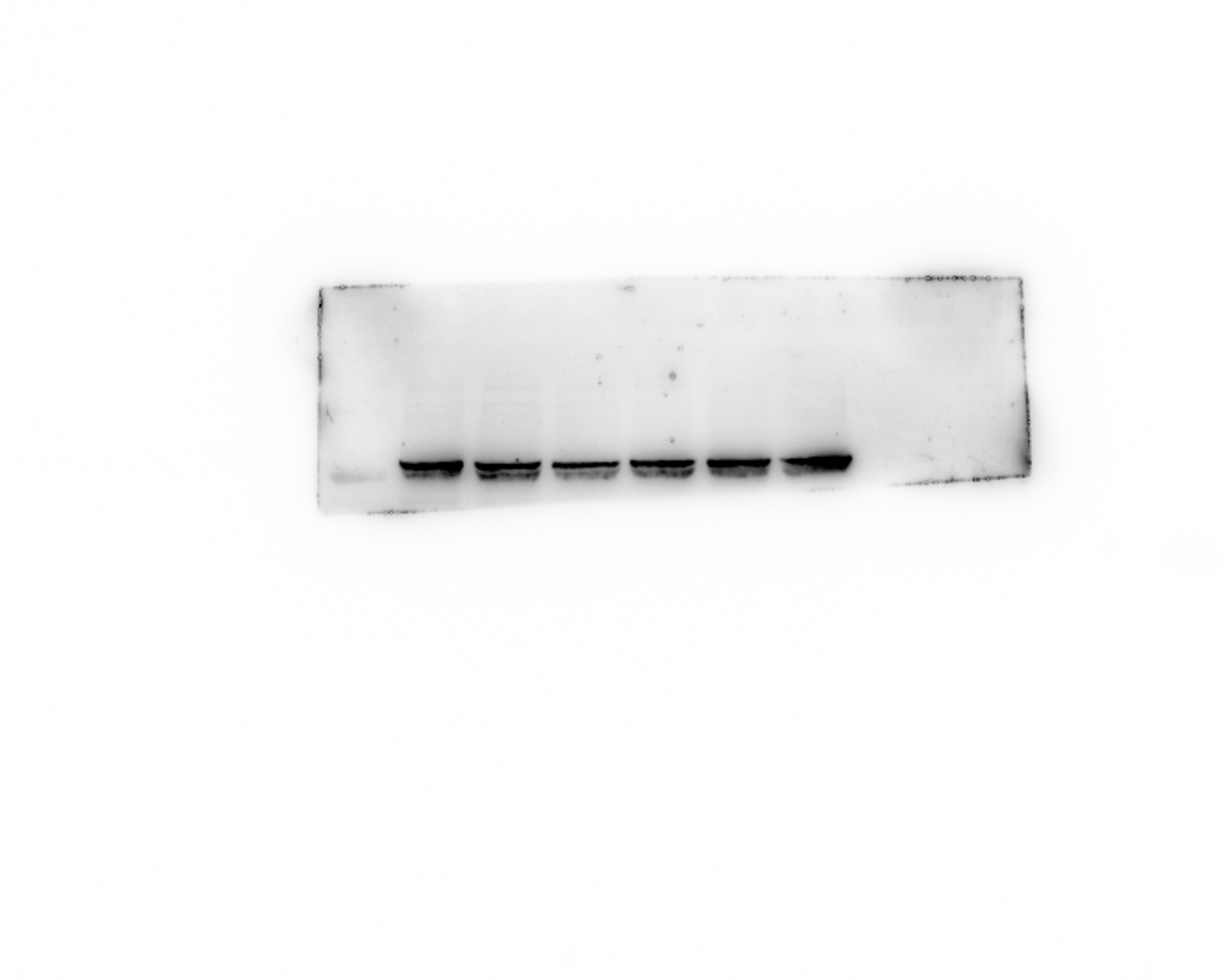

Supplement: Supplementary file 11 — Additional file 11. A compressed file that included our original uncropped gel/blot images [file 12915_2022_1423_MOESM11_ESM.zip › Additonal file 11/Figure 3L/b-Catenin.tif]

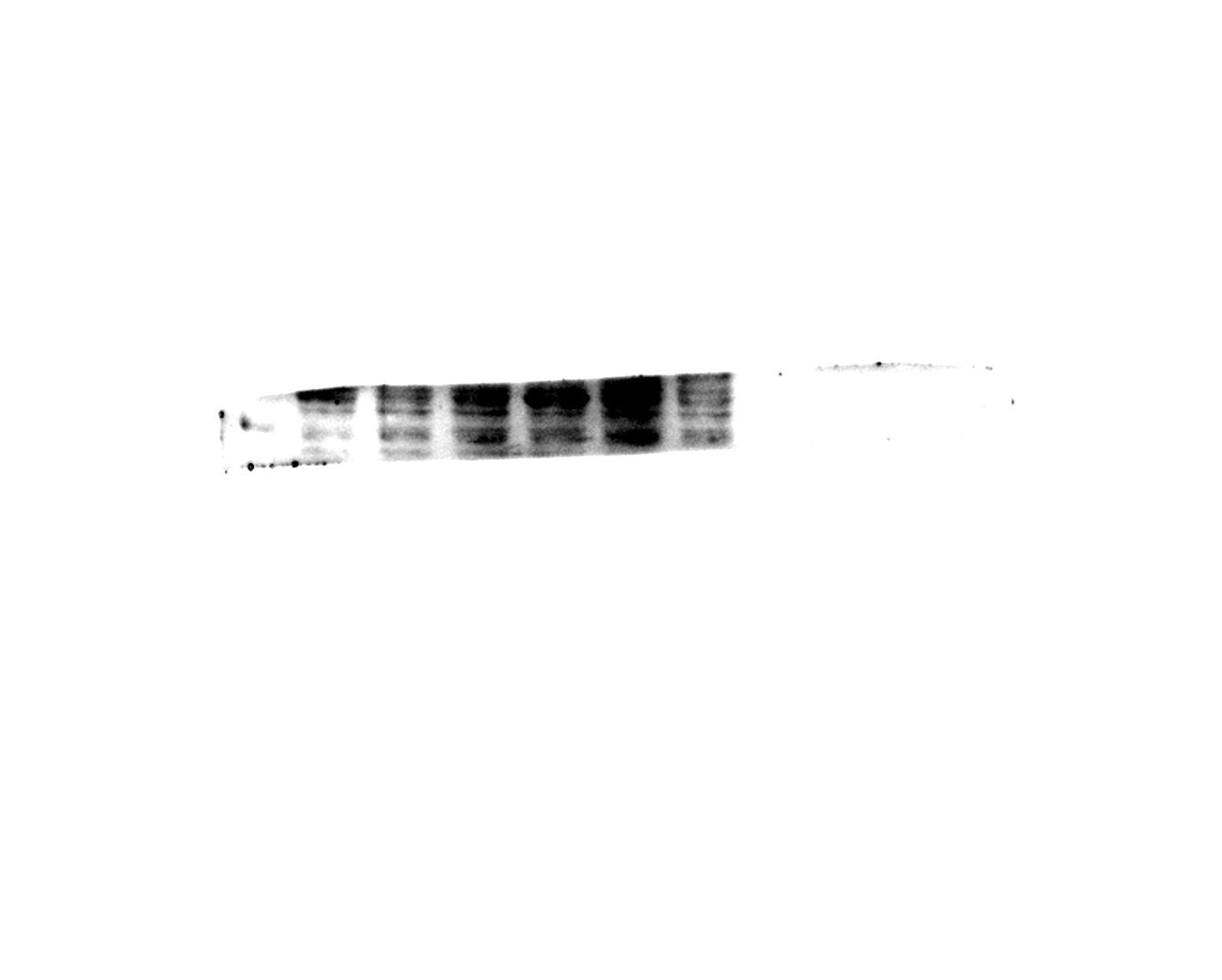

Supplement: Supplementary file 11 — Additional file 11. A compressed file that included our original uncropped gel/blot images [file 12915_2022_1423_MOESM11_ESM.zip › Additonal file 11/Figure 3L/p-AKT.tif]

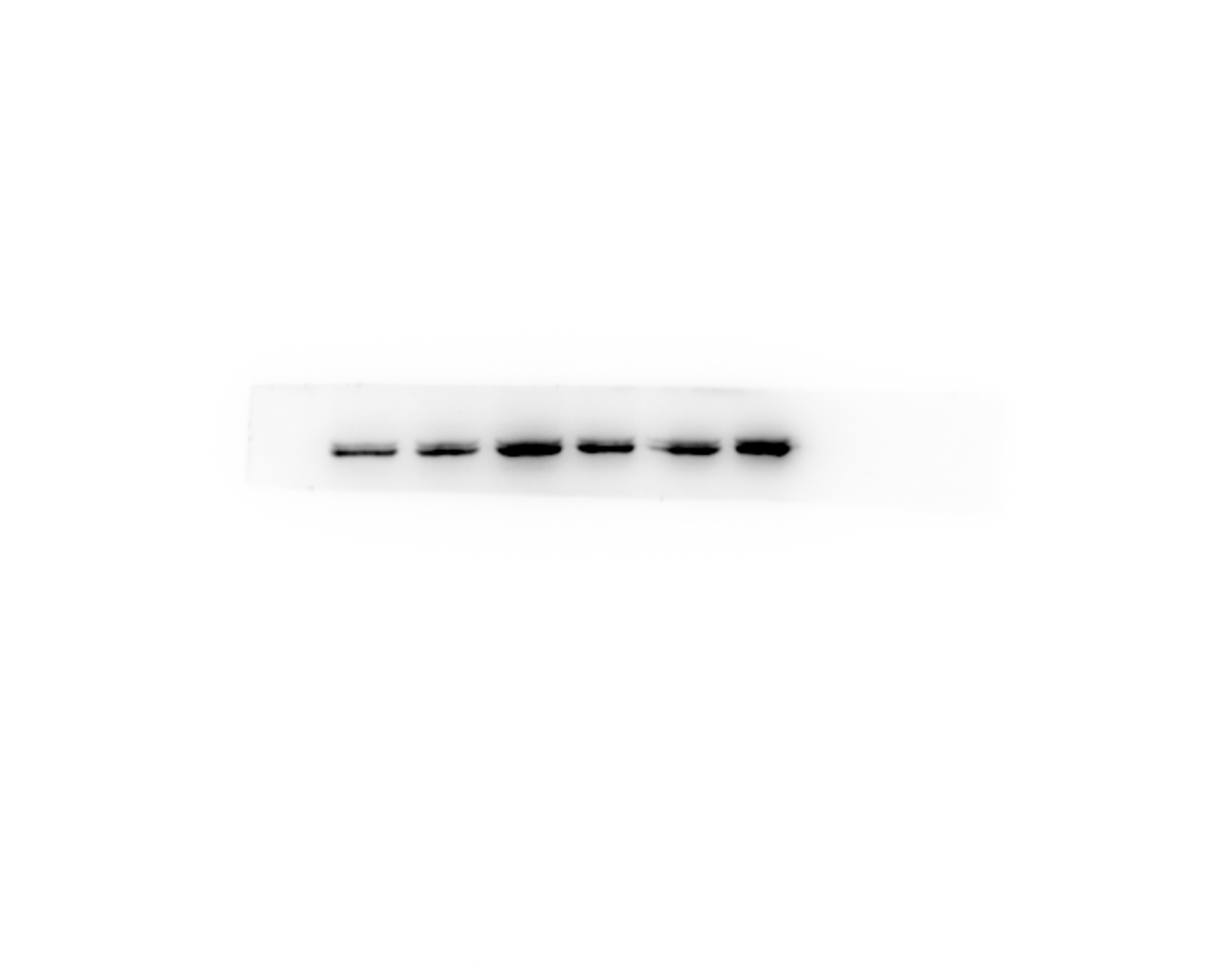

Supplement: Supplementary file 11 — Additional file 11. A compressed file that included our original uncropped gel/blot images [file 12915_2022_1423_MOESM11_ESM.zip › Additonal file 11/Figure 3L/p-GSK3b.tif]

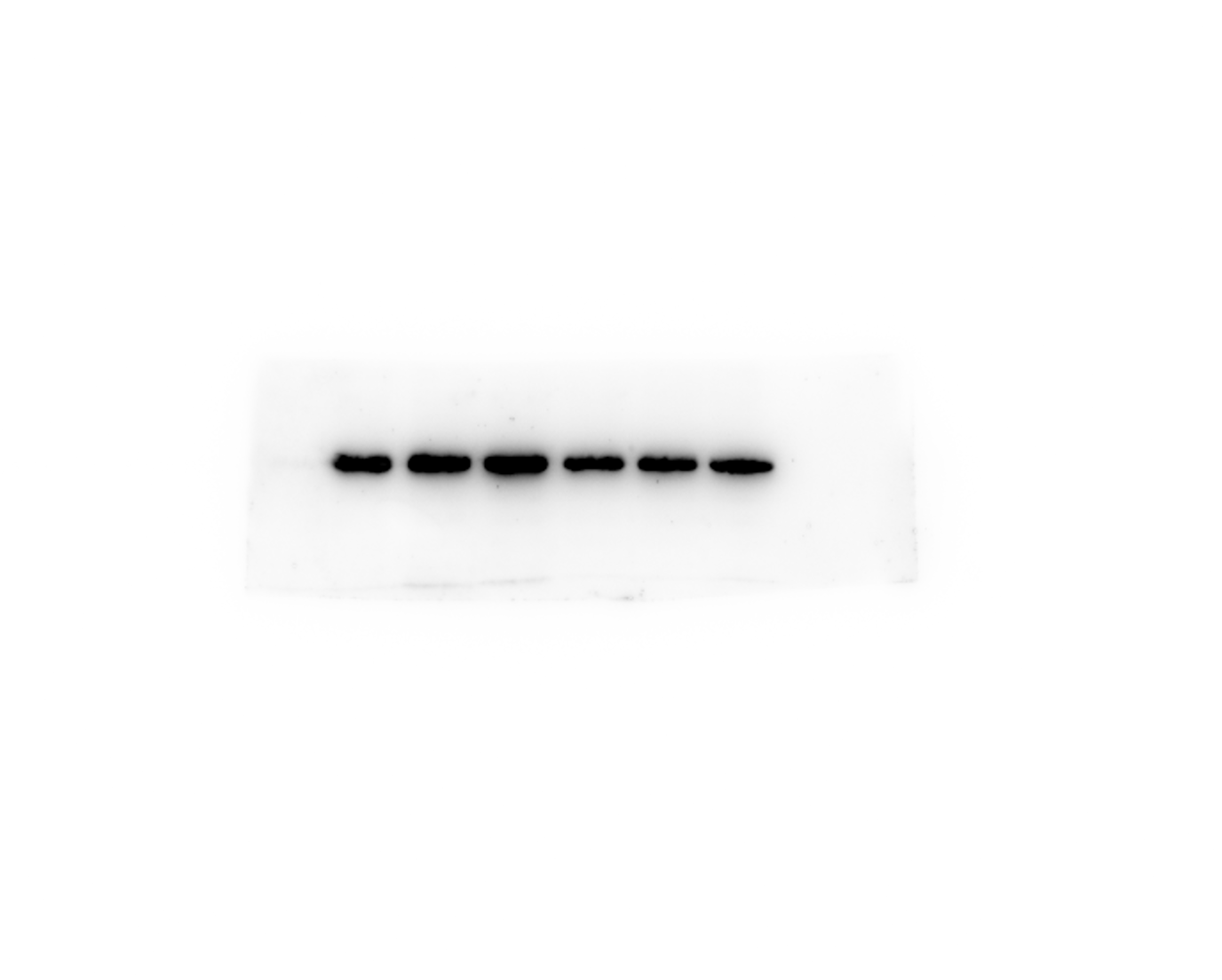

Supplement: Supplementary file 11 — Additional file 11. A compressed file that included our original uncropped gel/blot images [file 12915_2022_1423_MOESM11_ESM.zip › Additonal file 11/Figure 4B/Gapdh.tif]

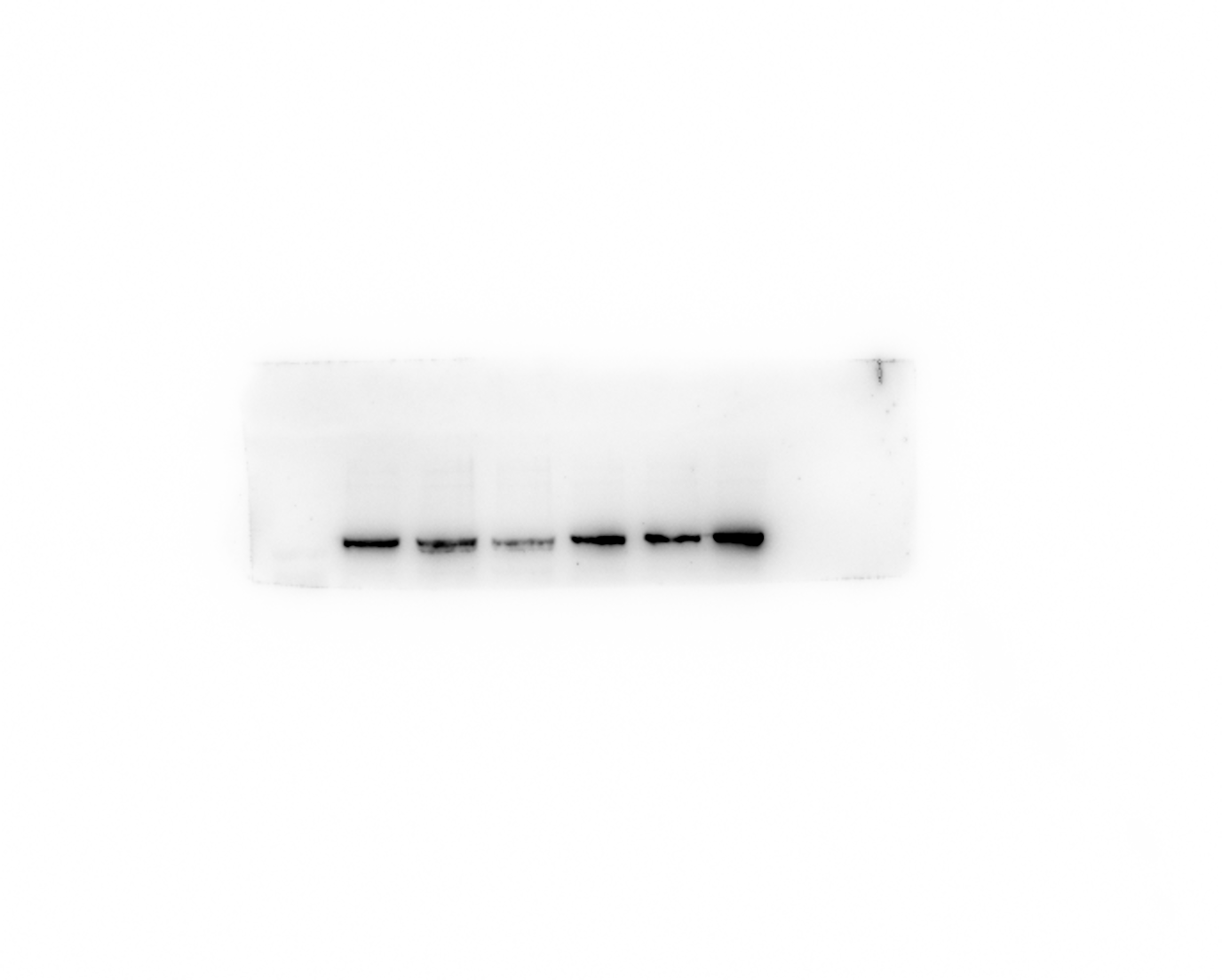

Supplement: Supplementary file 11 — Additional file 11. A compressed file that included our original uncropped gel/blot images [file 12915_2022_1423_MOESM11_ESM.zip › Additonal file 11/Figure 4B/b-Catenin.tif]

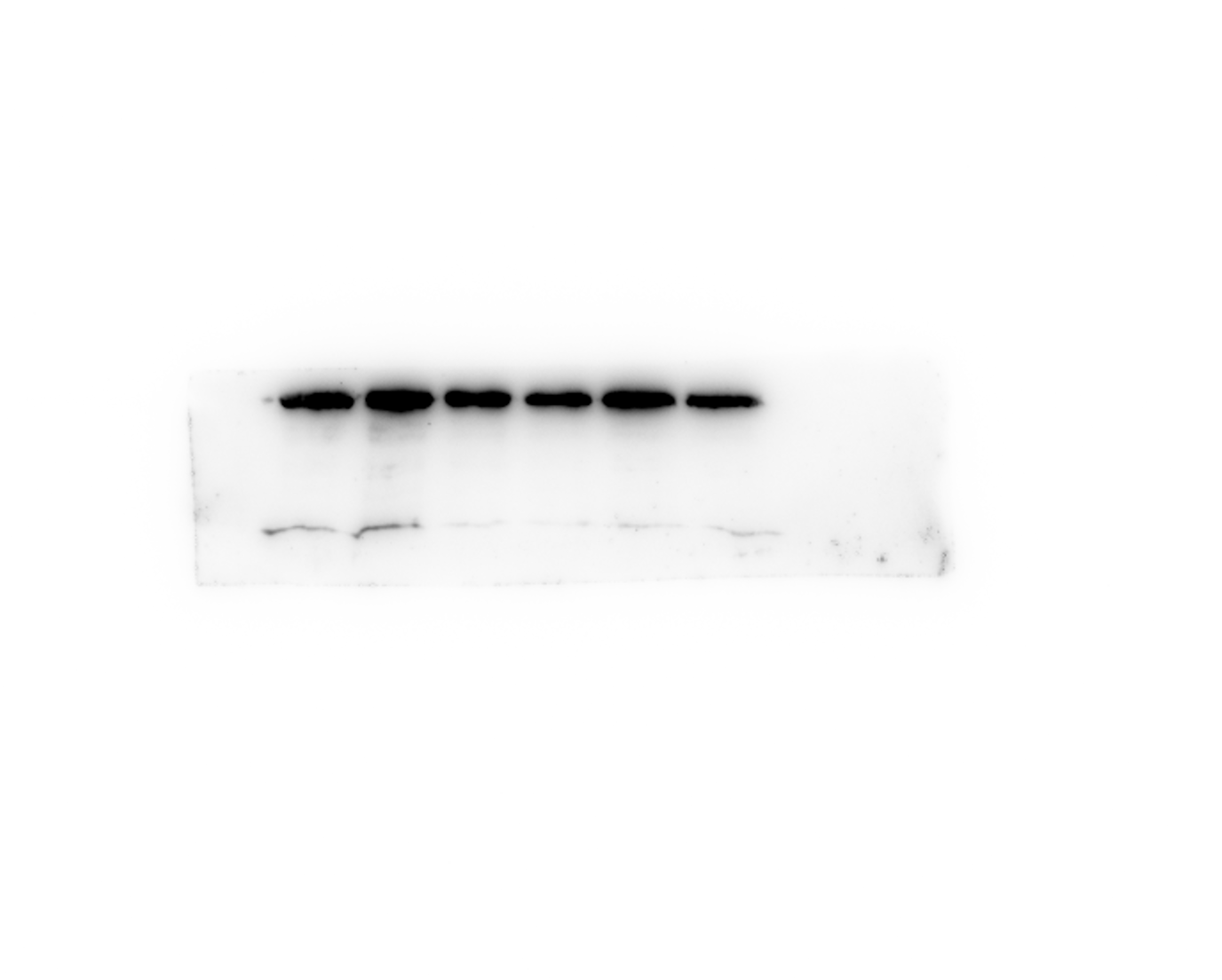

Supplement: Supplementary file 11 — Additional file 11. A compressed file that included our original uncropped gel/blot images [file 12915_2022_1423_MOESM11_ESM.zip › Additonal file 11/Figure 4D/Gapdh.tif]

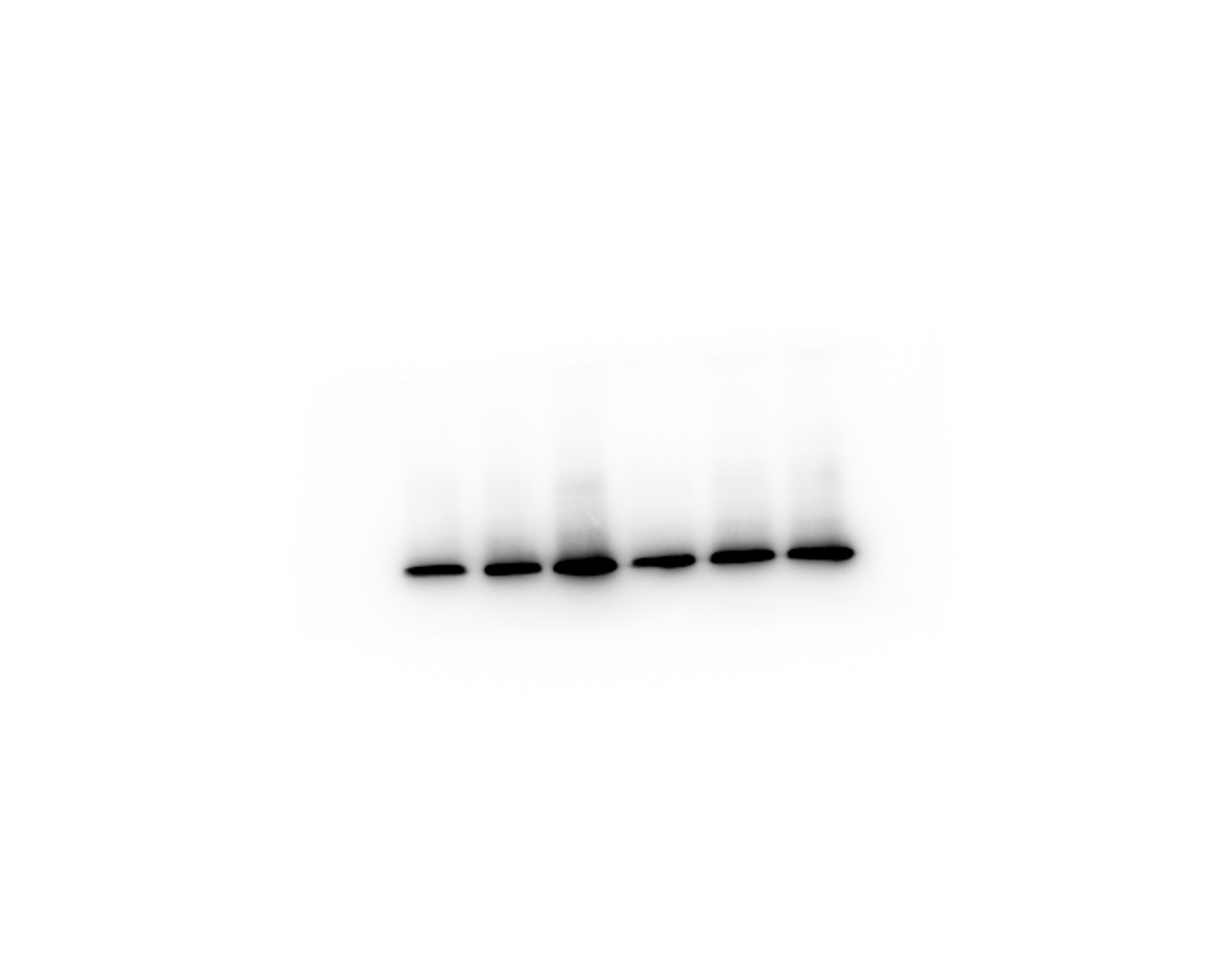

Supplement: Supplementary file 11 — Additional file 11. A compressed file that included our original uncropped gel/blot images [file 12915_2022_1423_MOESM11_ESM.zip › Additonal file 11/Figure 4D/H3.tif]

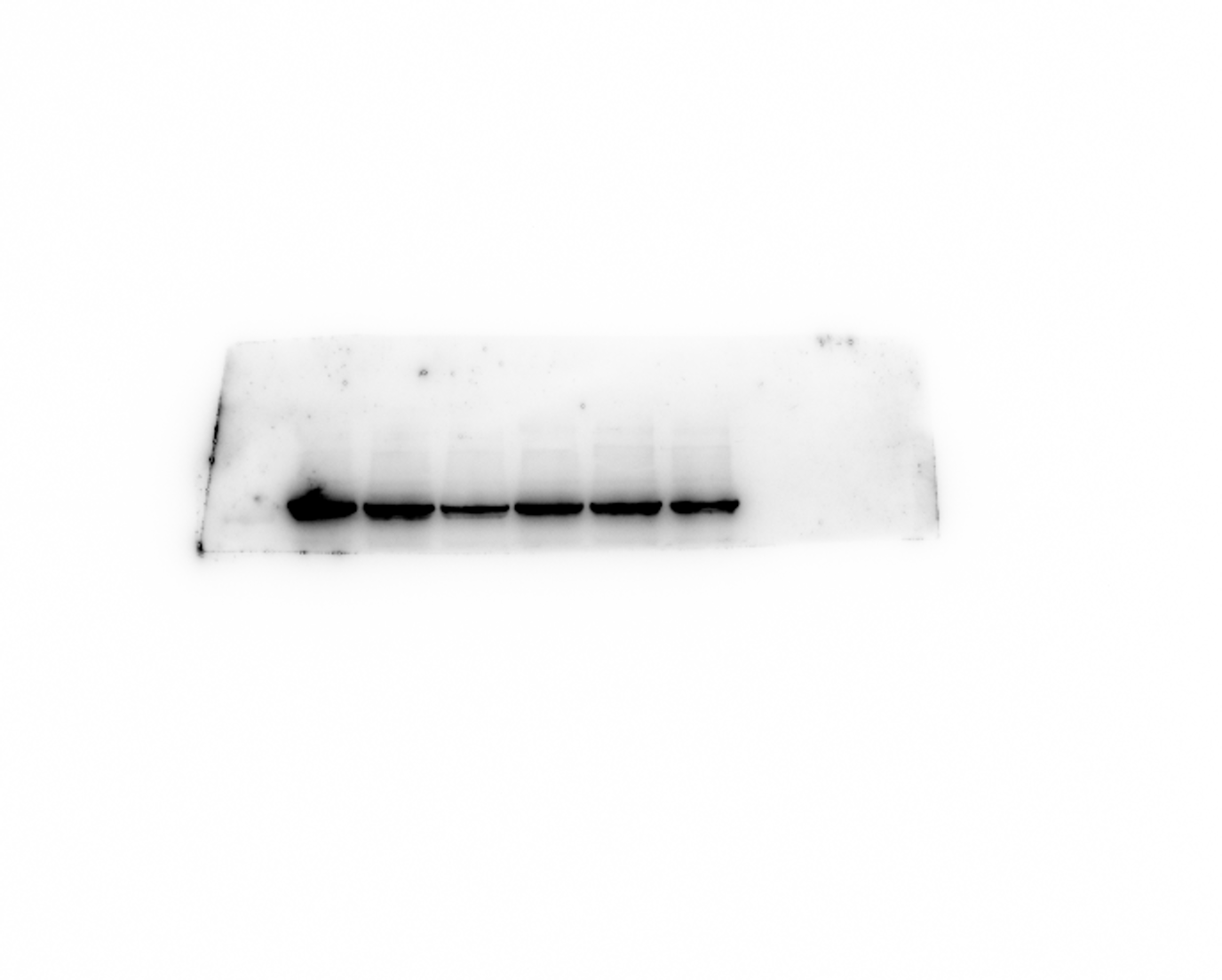

Supplement: Supplementary file 11 — Additional file 11. A compressed file that included our original uncropped gel/blot images [file 12915_2022_1423_MOESM11_ESM.zip › Additonal file 11/Figure 4D/b-Catenin (Cyto).tif]

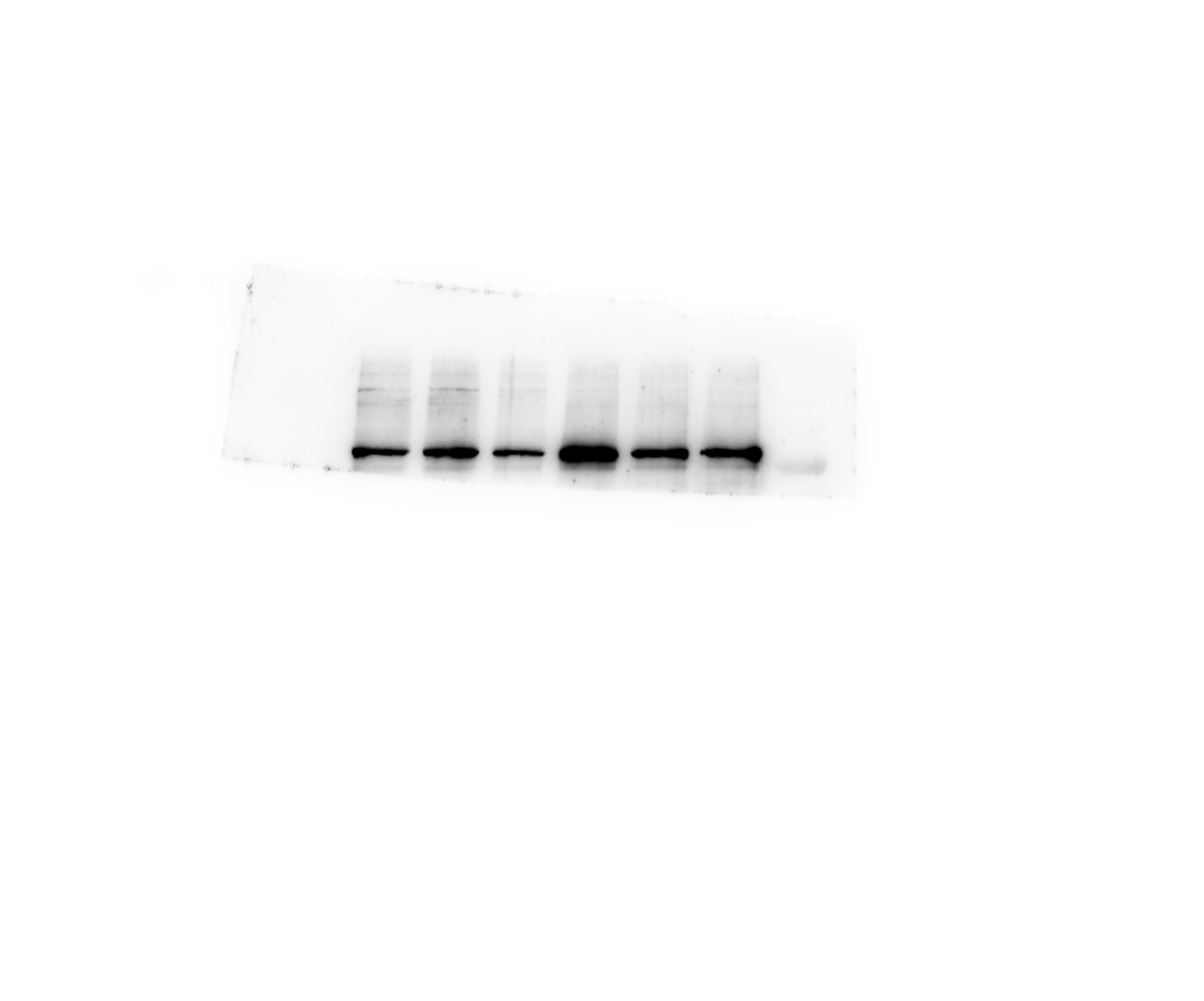

Supplement: Supplementary file 11 — Additional file 11. A compressed file that included our original uncropped gel/blot images [file 12915_2022_1423_MOESM11_ESM.zip › Additonal file 11/Figure 4D/b-Catenin (Nuclear).tif]

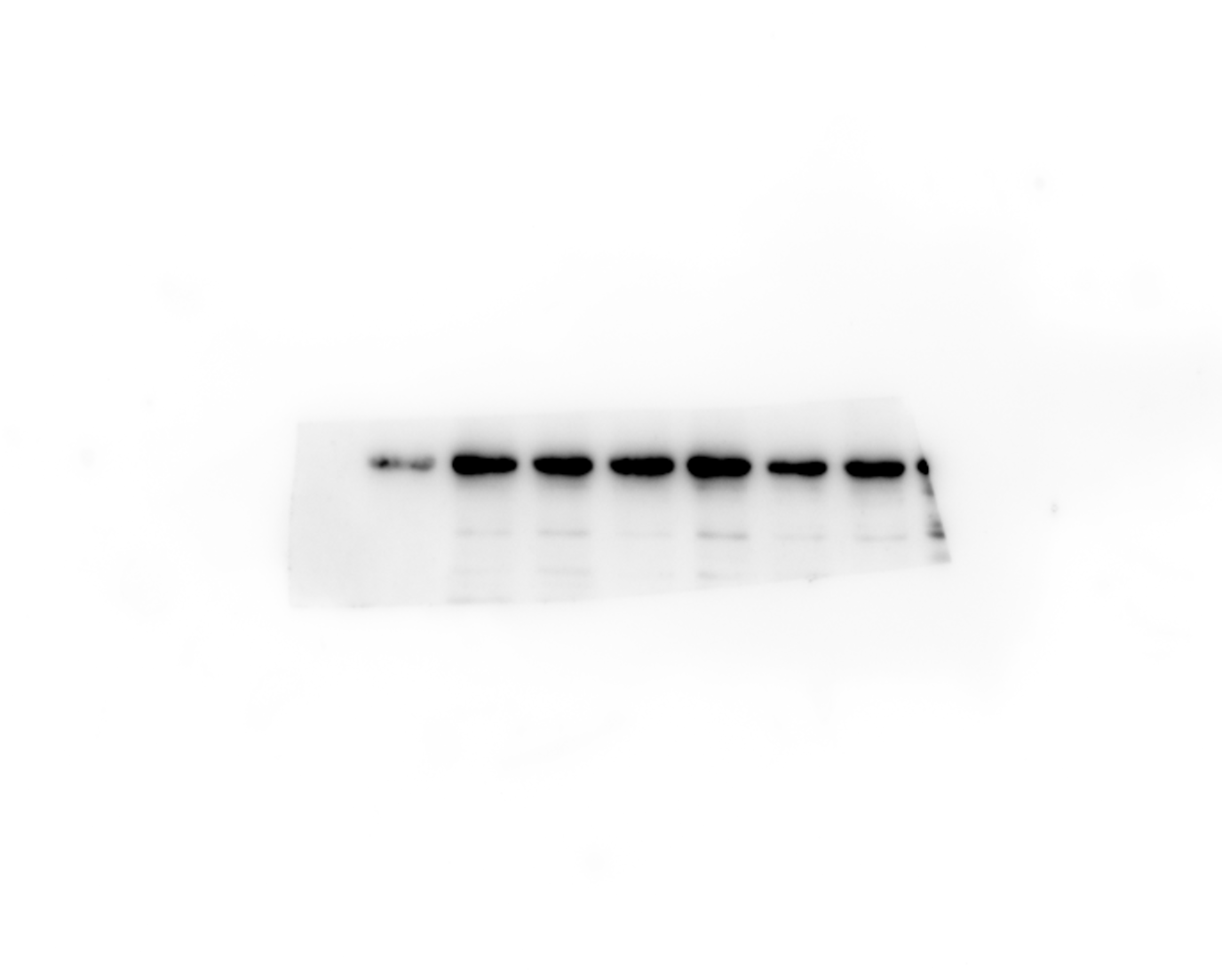

Supplement: Supplementary file 11 — Additional file 11. A compressed file that included our original uncropped gel/blot images [file 12915_2022_1423_MOESM11_ESM.zip › Additonal file 11/Figure 6A/Gapdh.tif]

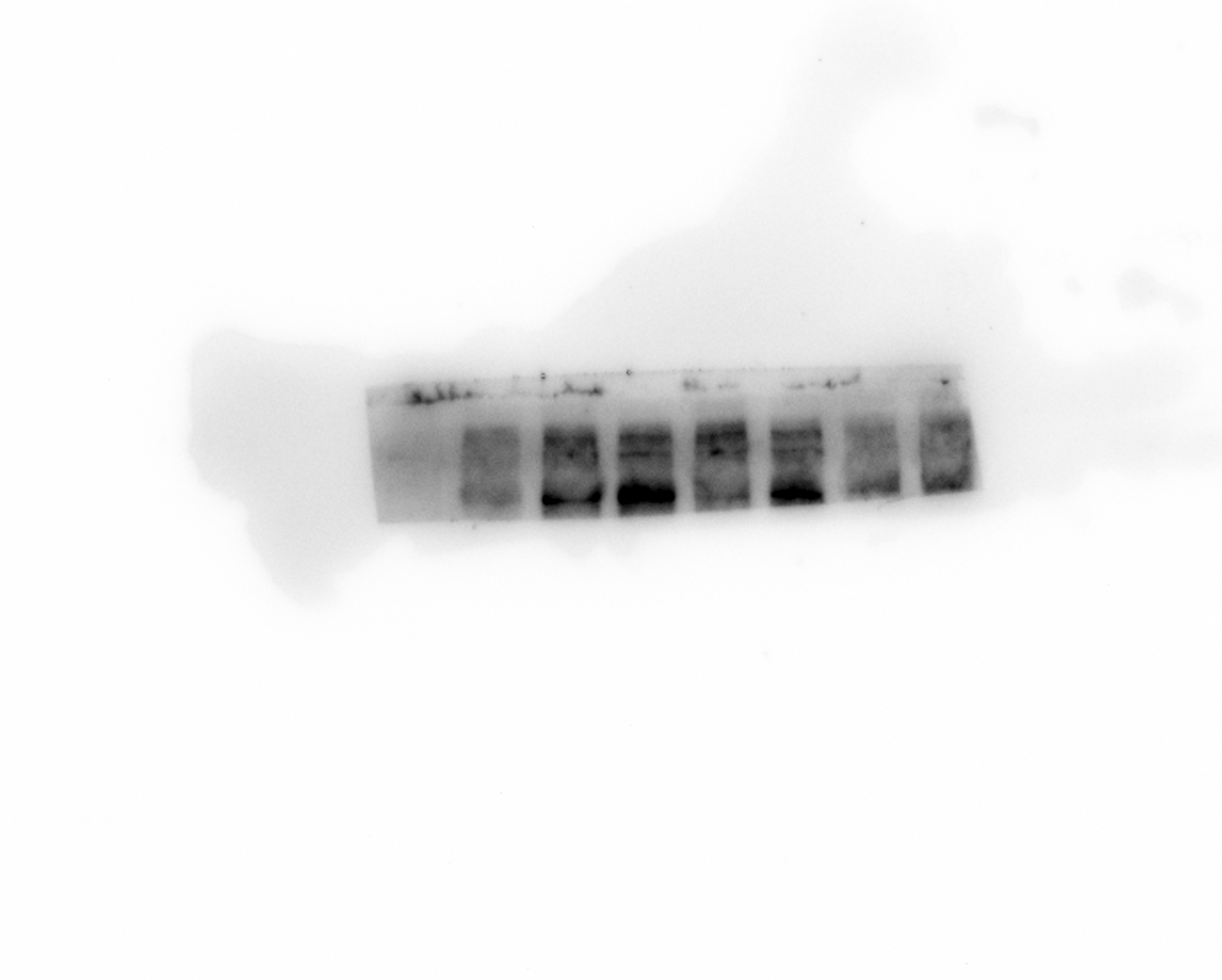

Supplement: Supplementary file 11 — Additional file 11. A compressed file that included our original uncropped gel/blot images [file 12915_2022_1423_MOESM11_ESM.zip › Additonal file 11/Figure 6A/Hif-1a.tif]

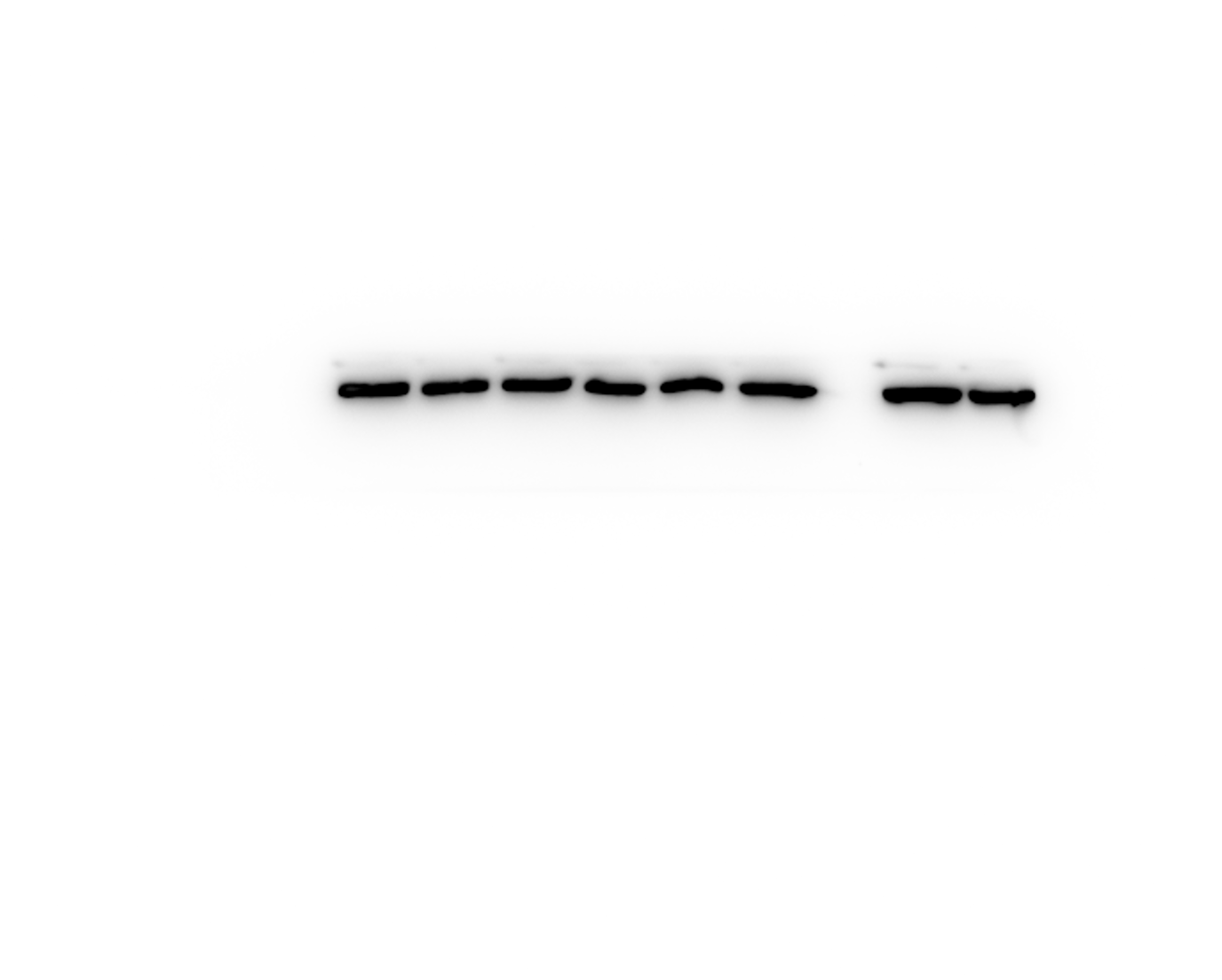

Supplement: Supplementary file 11 — Additional file 11. A compressed file that included our original uncropped gel/blot images [file 12915_2022_1423_MOESM11_ESM.zip › Additonal file 11/Figure 6E/Gapdh.tif]

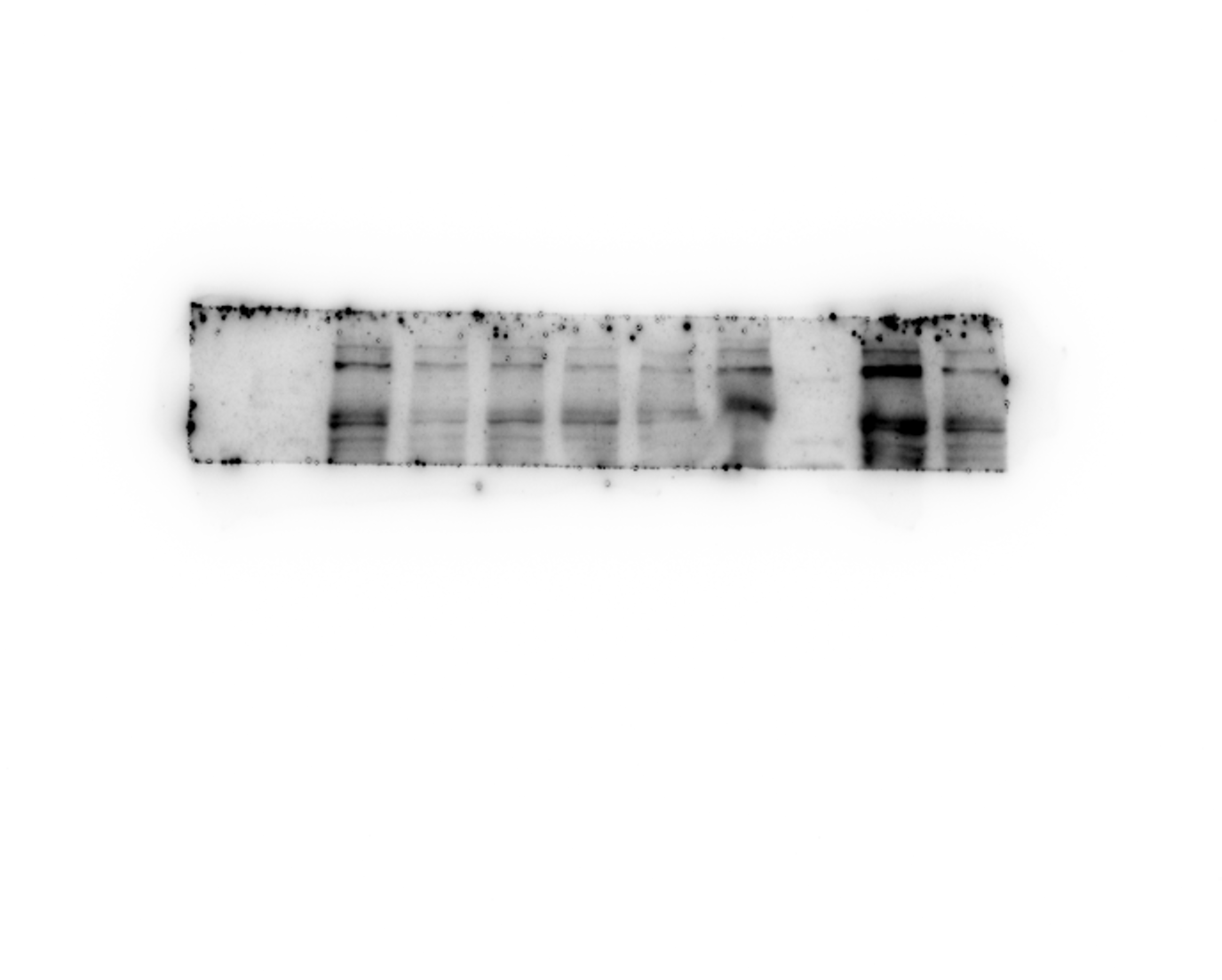

Supplement: Supplementary file 11 — Additional file 11. A compressed file that included our original uncropped gel/blot images [file 12915_2022_1423_MOESM11_ESM.zip › Additonal file 11/Figure 6E/Hif-1a.tif]

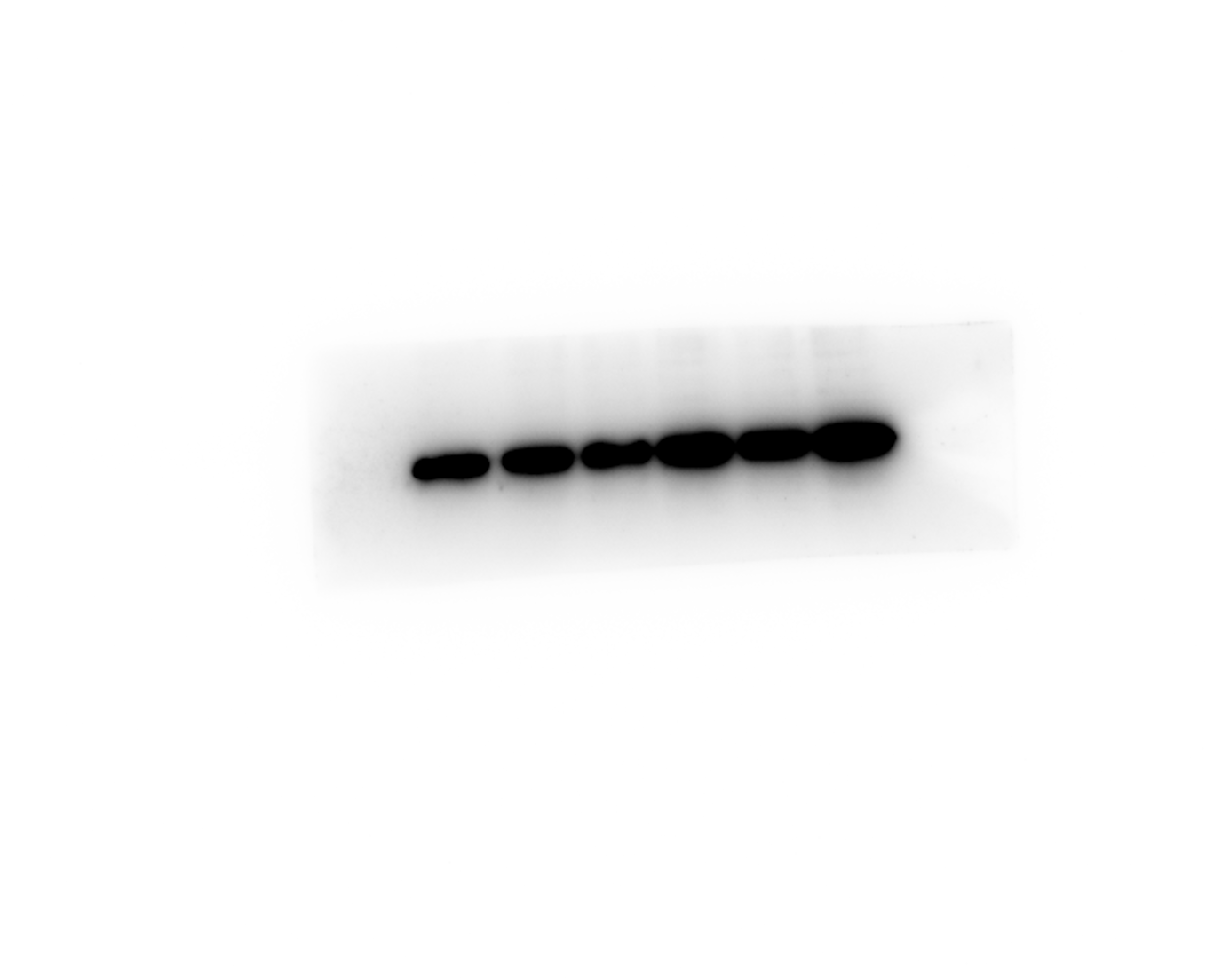

Supplement: Supplementary file 11 — Additional file 11. A compressed file that included our original uncropped gel/blot images [file 12915_2022_1423_MOESM11_ESM.zip › Additonal file 11/Figure 7E/Gapdh.tif]

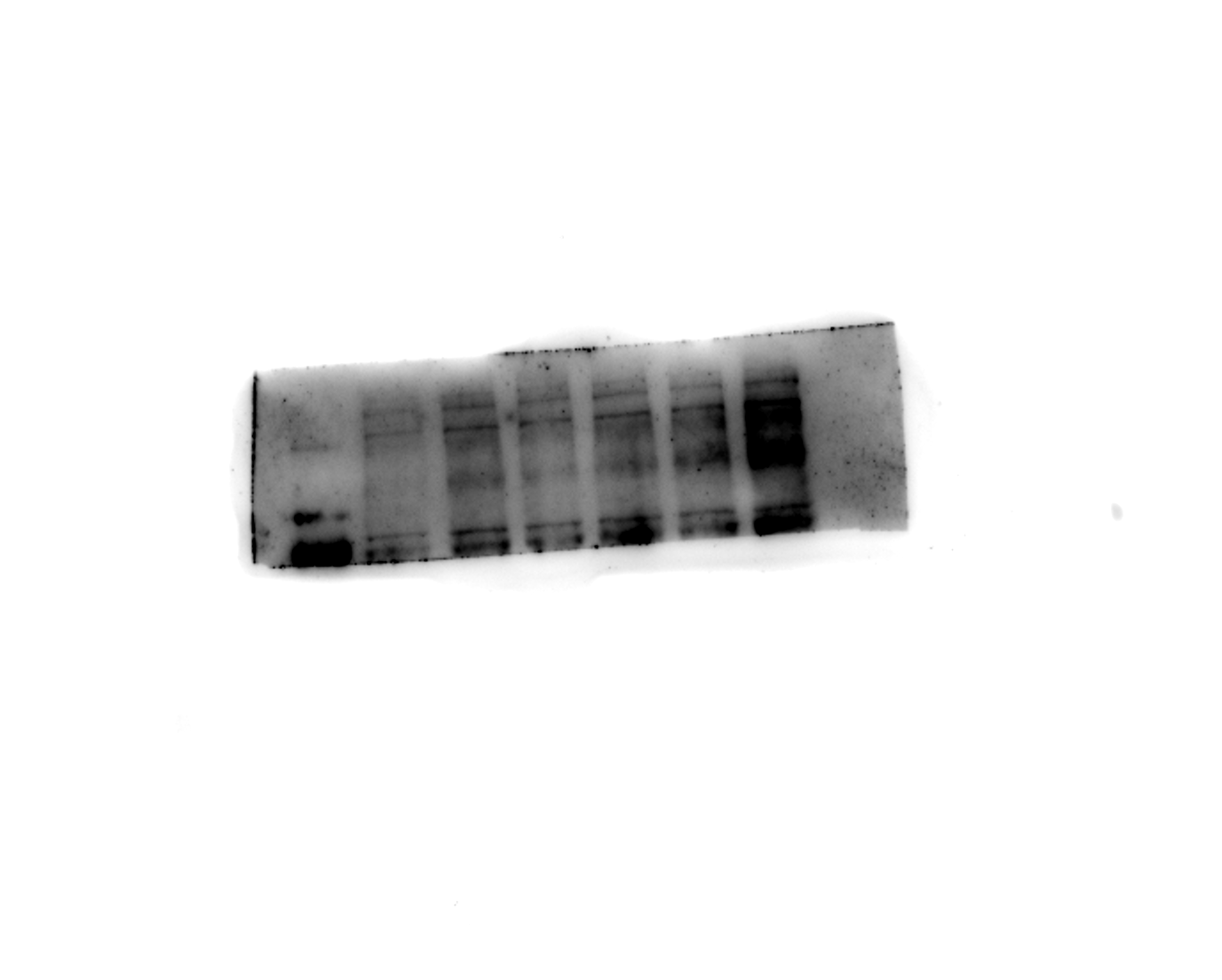

Supplement: Supplementary file 11 — Additional file 11. A compressed file that included our original uncropped gel/blot images [file 12915_2022_1423_MOESM11_ESM.zip › Additonal file 11/Figure 7E/Hif-1a.tif]

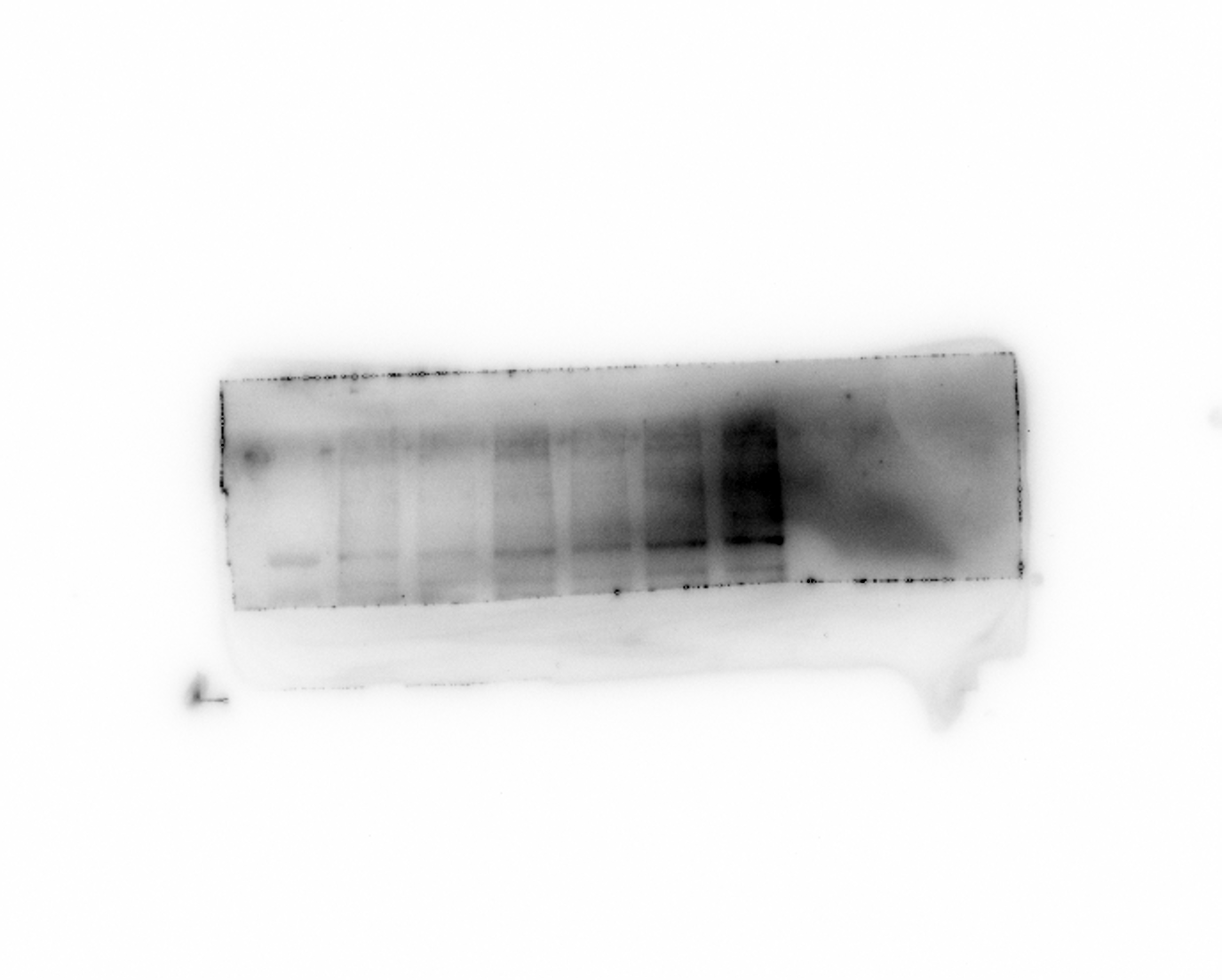

Supplement: Supplementary file 11 — Additional file 11. A compressed file that included our original uncropped gel/blot images [file 12915_2022_1423_MOESM11_ESM.zip › Additonal file 11/Figure 7E/Myc.tif]

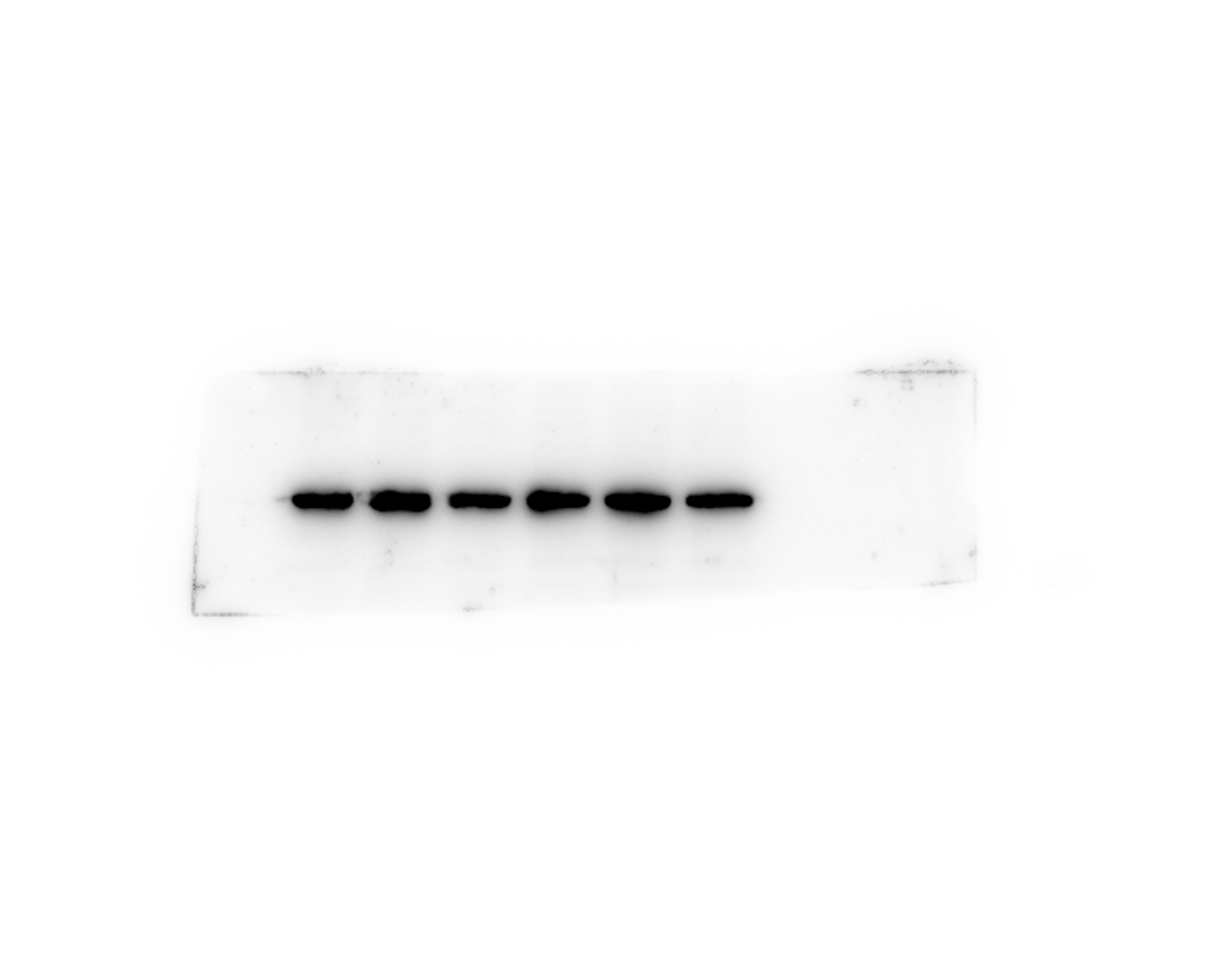

Supplement: Supplementary file 11 — Additional file 11. A compressed file that included our original uncropped gel/blot images [file 12915_2022_1423_MOESM11_ESM.zip › Additonal file 11/Figure S5E/Gapdh.tif]

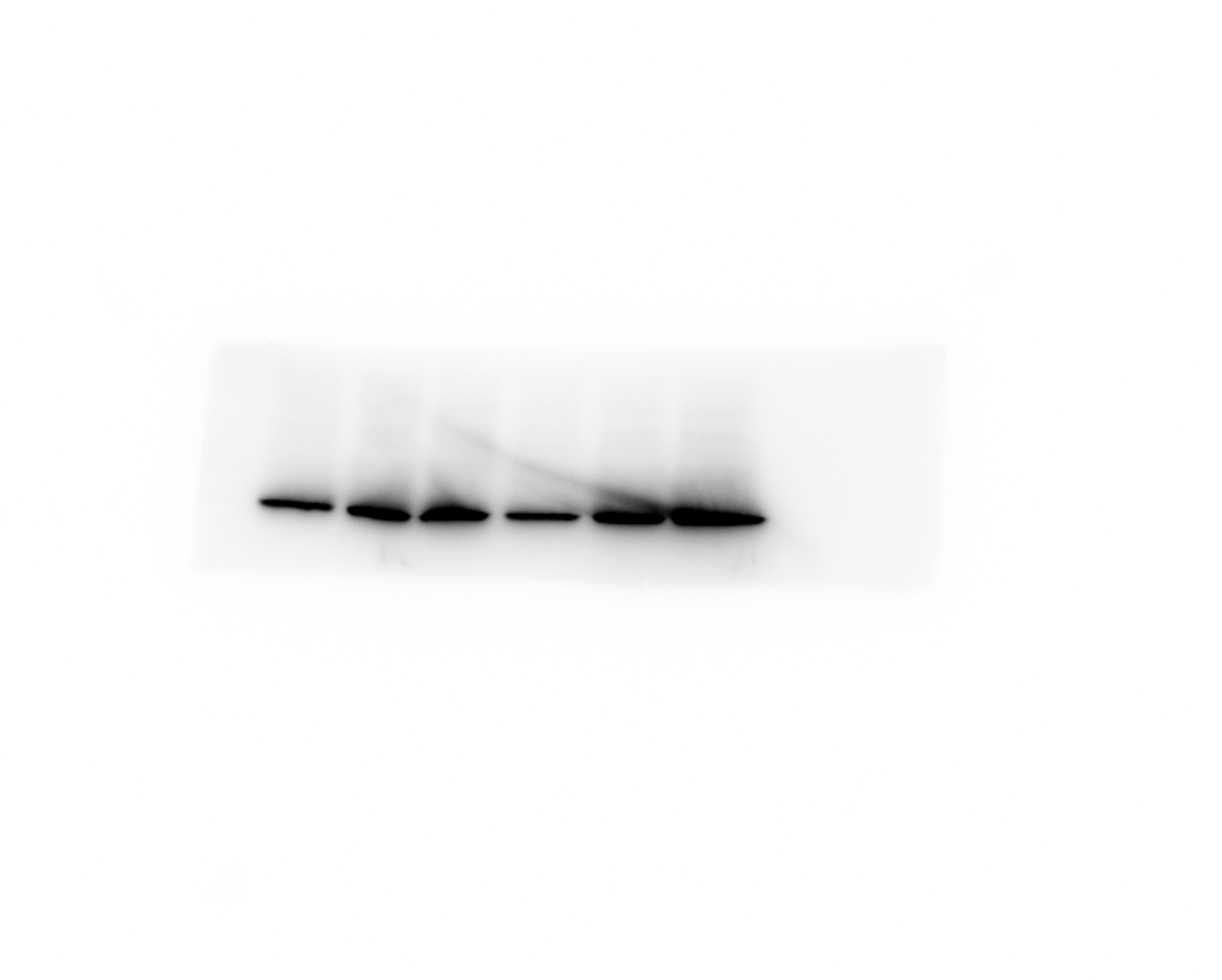

Supplement: Supplementary file 11 — Additional file 11. A compressed file that included our original uncropped gel/blot images [file 12915_2022_1423_MOESM11_ESM.zip › Additonal file 11/Figure S5E/H3.tif]

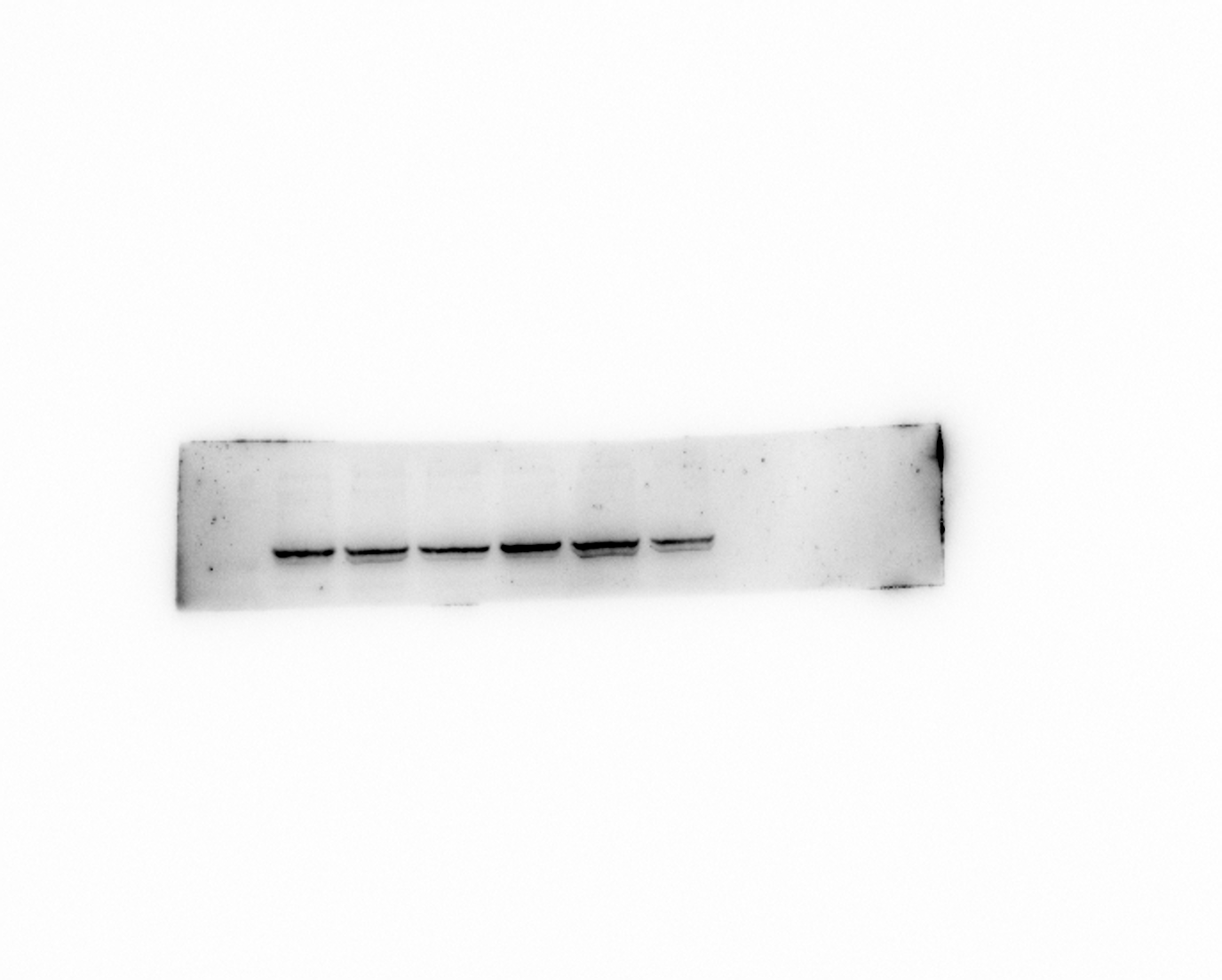

Supplement: Supplementary file 11 — Additional file 11. A compressed file that included our original uncropped gel/blot images [file 12915_2022_1423_MOESM11_ESM.zip › Additonal file 11/Figure S5E/b-Catenin (Cyto).tif]

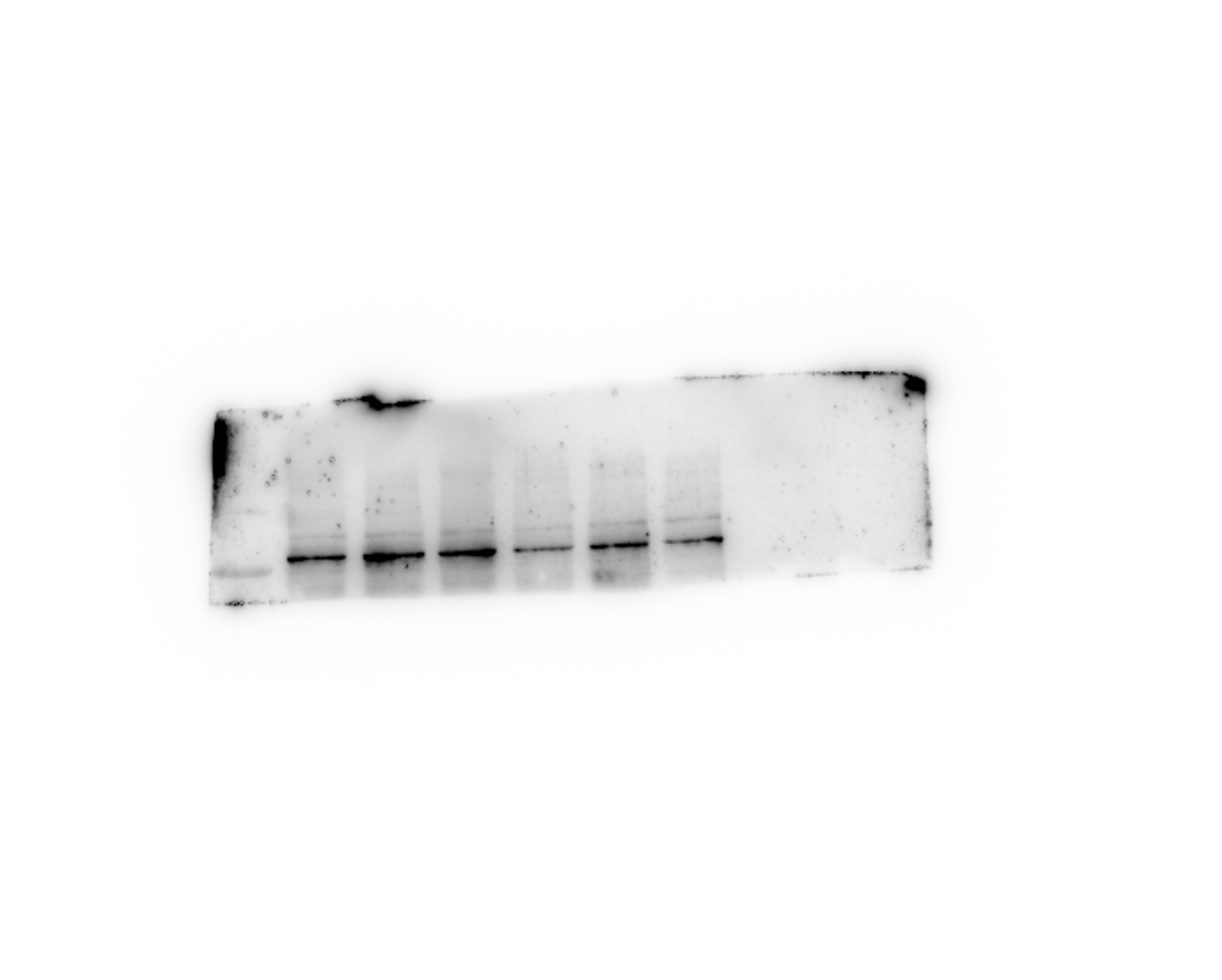

Supplement: Supplementary file 11 — Additional file 11. A compressed file that included our original uncropped gel/blot images [file 12915_2022_1423_MOESM11_ESM.zip › Additonal file 11/Figure S5E/b-Catenin (Nuclear).tif]

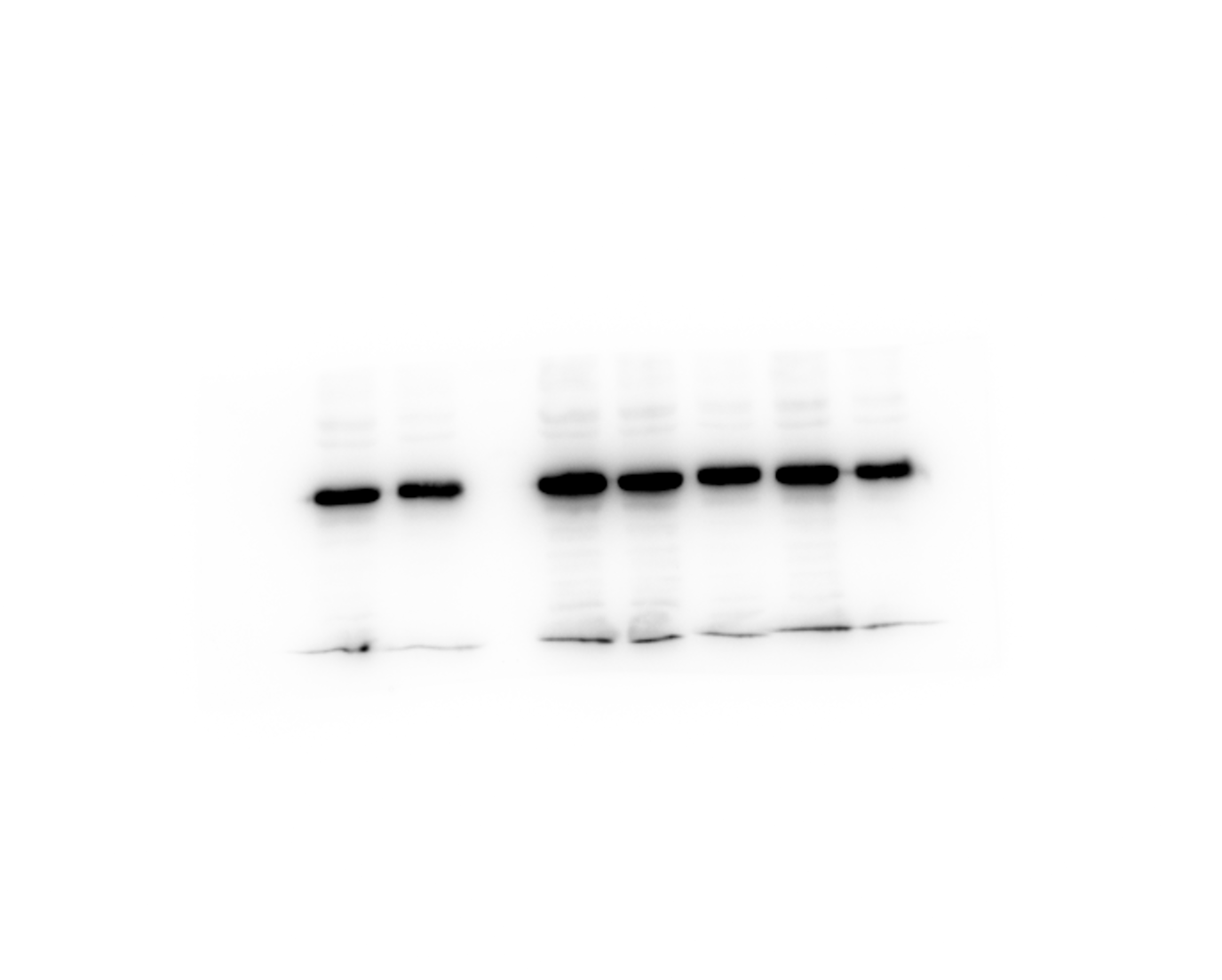

Supplement: Supplementary file 11 — Additional file 11. A compressed file that included our original uncropped gel/blot images [file 12915_2022_1423_MOESM11_ESM.zip › Additonal file 11/Figure S7B/Gapdh.tif]

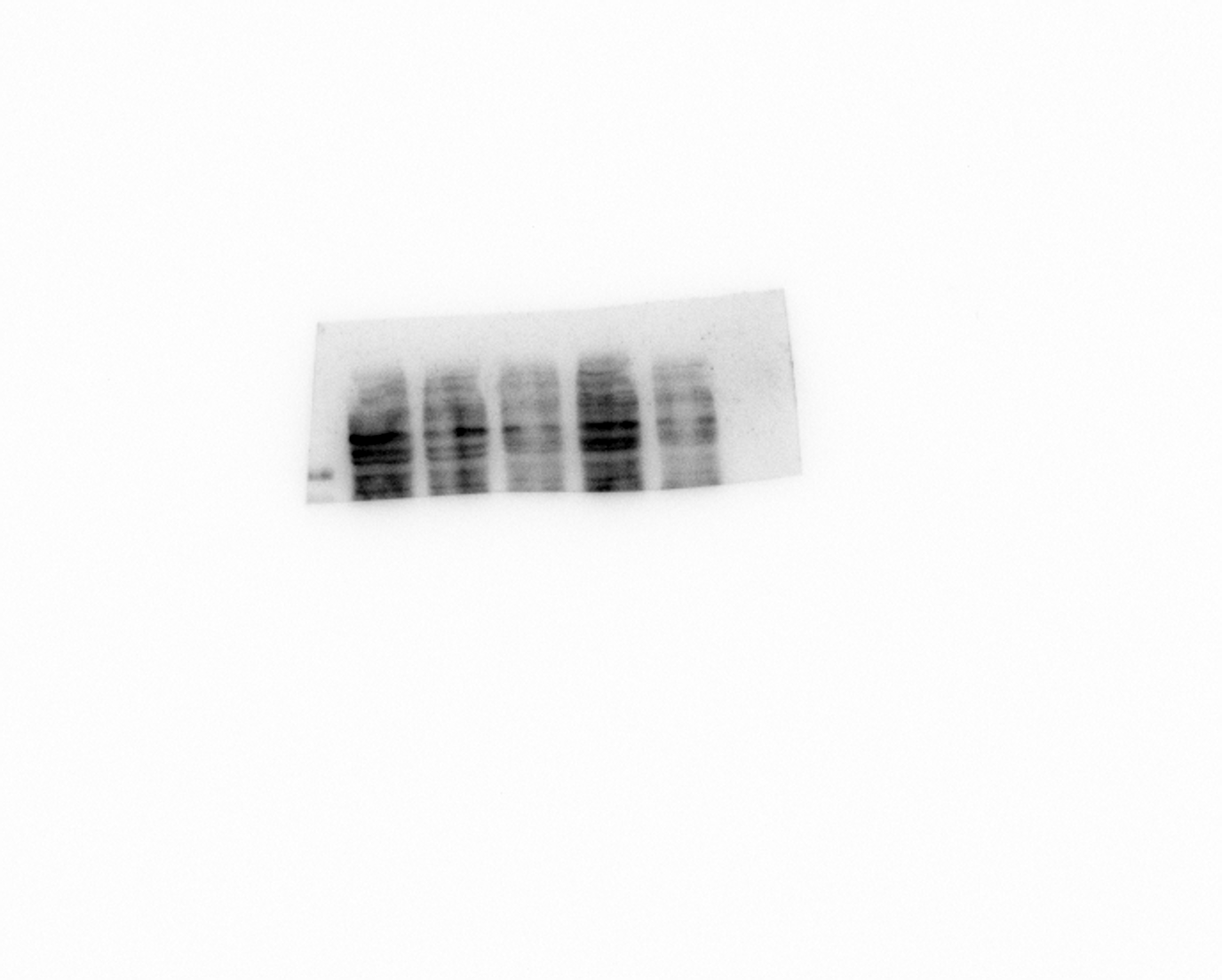

Supplement: Supplementary file 11 — Additional file 11. A compressed file that included our original uncropped gel/blot images [file 12915_2022_1423_MOESM11_ESM.zip › Additonal file 11/Figure S7B/Hif-1a.tif]

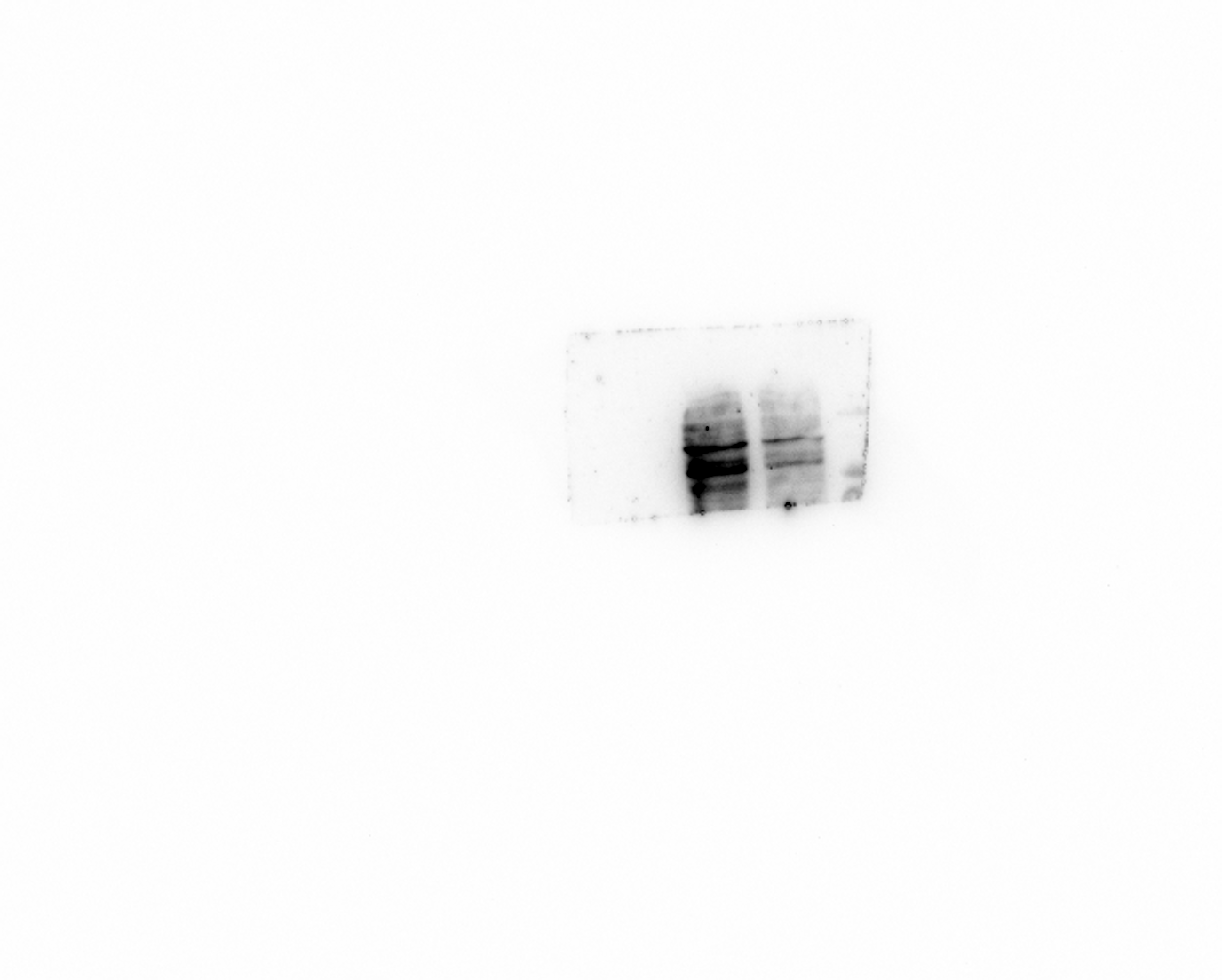

Supplement: Supplementary file 11 — Additional file 11. A compressed file that included our original uncropped gel/blot images [file 12915_2022_1423_MOESM11_ESM.zip › Additonal file 11/Figure S7B/Myc.tif]
