# Supplementary material for: Hypoxia is fine-tuned by Hif-1α and regulates mesendoderm differentiation through the Wnt/β-Catenin pathway
Source: BMC Biol. 2022 Oct 5;20:219. doi: 10.1186/s12915-022-01423-y (PMC9536055; doi:10.1186/s12915-022-01423-y)
Supplement: Supplementary file 9 — Additional file 9: Table S1. List of primers used for RT-qPCR. [file 12915_2022_1423_MOESM9_ESM.docx]

**Table S1. List of primers used for RT-qPCR**

| **Primers** | **Sequences(5’-3’)** |
| --- | --- |
| T-brachyury-F | TGATCACCAGCCACTGCTTT |
| T-brachyury-R | AGCCACCCCCATTGGGAATA |
| Eomes-F | GGAAGTGACAGAGGACGGTG |
| Eomes-R | AGCCGTGTACATGGAATCGT |
| Mesp1-F | GAGTCGCCGCAGAATCGT |
| Mesp1-R | TGAAGAGCGGAGATGAGGGAC |
| Gsc-F | GACGAAGTACCCAGACGTGG |
| Gsc-R | CGGTTCTTAAACCAGACCTCCA |
| Pax6-F | TGGCAAACAACCTGCCTATG |
| Pax6-R | TGCACGAGTATGAGGAGGTCT |
| Nestin-F | CCTCAACCCTCACCACTCTATTTT |
| Nestin-R | GCTTTTTACTGTCCCCGAGTTCTC |
| Sox17-F | GAGGGCCAGAAGCAGTGTTACAC |
| Sox17-R | CTGGCTAAAACTGGACAGTGATTGTG |
| Cxcr4-F | GTCTCCACGGAGTCAGAATC |
| Cxcr4-R | GGAACATAAACTCCCAACAGTC |
| Nkx2.5-F | AAGTGCTCTCCTGCTTTCCC |
| Nkx2.5-R | GCGCGCACAGCTCTTTTT |
| Tbx5-F | CAAGGCAGGGAGGAGAATGTT |
| Tbx5-R | GCTCTGCTTTGCCAGTTACG |
| Mef2C-F | CGGTGTCGTCAGTTGTATGG |
| Mef2C-R | TGCAGTAGATATGCGGCTTG |
| α-MHC-F | AGGTGGATGATCTGGAGGGA |
| α-MHC-R | TCTGCTGACTGATGTCGAACT |
| Pecam1-F | AGCCTAGTGTGGAAGCCAAC |
| Pecam1-R | ATCCATGTTCTGGGGGTCTTT |
| Acta2-F | TGGAAAAGATCTGGCACCAC |
| Acta2-R | TCTGTCAGCAGTGTCGGATG |
| Wnt3-F | CTACCCAATTTGGTGGTCCCT |
| Wnt3-R | CTCGATGTAATTGCGGCAGA |
| Wnt8a-F | CTGCCTGGTCAGTGAACAACTT |
| Wnt8a-R | GATGCCAATCTGAGCTCCCAA |
| Sp5-F | TCCTCCGGAACGACTCACT |
| Sp5-R | GGCAGTCCAAGGGTGGAAAA |
| Cdx1-F | GTACATCACTATCCGGCGCA |
| Cdx1-R | ACCAGATCTTTACCTGCCGC |
| Egln1-F | AGGCTATGTCCGTCACGTTG |
| Egln1-R | TATTGCGTACCTTGGCGTCC |
| Pgk-F | TGCTGGAAAACCTCCGCTTT |
| Pgk-R | CCGGCTCAGCTTTAACCTTG |
| Ldha-F | GAGTGGTGTGAATGTTGCCG |
| Ldha-R | GATCACCTCGTAGGCACTGTC |
| Vegfa-F | GGAGAGATGAGCTTCCTACAGC |
| Vegfa-R | GCCTTGGCTTGTCACATTTTTCT |
| Gapdh-F | CAAGCTCATTTCCTGGTATGACAA |
| Gapdh-R | GGGATAGGGCCTCTCTTGCT |
